# Global burden of breast cancer and attributable risk factors in 204 countries and territories, from 1990 to 2021: results from the Global Burden of Disease Study 2021

**DOI:** 10.1186/s40364-024-00631-8

**Published:** 2024-08-26

**Authors:** Rui Sha, Xiang-meng Kong, Xin-yu Li, Ya-bing Wang

**Affiliations:** 1 Present Address: Department of Thyroid and Breast Surgery, The First Affiliated Hospital of Wannan Medical College (Yijishan Hospital of Wannan Medical College), Zheshan West Rd No. 2, Wuhu , Anhui Province, 241001 China; 2 Department of Cardiology, Shanghai Ninth People,s Hospital, Shanghai Jiao Tong University School of Medicine, No.639 Zhizaoju Road, Shanghai, Huangpu District 200011 China; 3 Department of Plastic and Reconstructive Surgery, Shanghai Ninth People’s Hospital, Shanghai Jiao Tong University School of Medicine, Shanghai, China

**Keywords:** Breast cancer, Global burden of disease, Disability-adjusted life years, Incidence

## Abstract

**Background and objective:**

Breast cancer is a leading cause of morbidity and mortality among women worldwide. This study aimed to assess the global burden of breast cancer and identify attributable risk factors across 204 countries and territories from 1990 to 2021.

**Methods:**

Using data from the Global Burden of Disease Study 2021, we analyzed the incidence, mortality, disability-adjusted life years (DALYs), and risk factors associated with breast cancer. We obtained and analyzed the age-standardized incidence rate (ASIR), age-standardized death rate (ASDR), and age-standardized DALYs rate from 1990 to 2021. We assessed geographical variations and the impact of the Socio-demographic Index (SDI) using regression analysis and stratification by SDI quintiles. Additionally, we estimated the risk factors attributable to breast cancer deaths and DALYs using the comparative risk assessment framework of the GBD study.

**Results:**

Globally, breast cancer incident cases increased from 875,657 in 1990 to 2,121,564 in 2021. The ASIR rose from 16.42 to 26.88 per 100,000 (95% CI: 1.54–1.60). High SDI regions showed the highest ASIR (66.89 per 100,000 in 2021), while Low SDI regions had the lowest (6.99 per 100,000 in 2021). The global ASDR decreased from 10.42 to 8.54 per 100,000, and the age-standardized DALYs rate decreased from 313.36 to 261.5 per 100,000 between 1990 and 2021. However, these improvements were not uniform across SDI regions. Risk factors included high body-mass index, alcohol use, tobacco, and high fasting plasma glucose, with variations across SDI regions.

**Conclusion:**

The global burden of breast cancer has increased significantly from 1990 to 2021, with disparities observed across SDI regions. While high SDI areas show improvements in mortality and DALYs, lower SDI regions face increasing burdens. Targeted interventions addressing modifiable risk factors and improving healthcare access in less developed regions are crucial for reducing the global impact of breast cancer.

**Supplementary Information:**

The online version contains supplementary material available at 10.1186/s40364-024-00631-8.

## Introduction

Breast cancer (BC) remains a significant global health concern, representing a substantial burden on healthcare systems worldwide. As the most commonly diagnosed cancer among women globally, it continues to be a leading cause of cancer-related deaths [1].

Over the past three decades, the landscape of BC has evolved considerably. Advancements in early detection methods, improved treatment modalities, and increased awareness have contributed to changes in incidence, mortality, and survival rates [2]. However, these improvements have not been uniform across all regions, with significant disparities persisting between high-income and low-to-middle-income countries [3]. The etiology of BC is multifactorial, involving a complex interplay of genetic, environmental, and lifestyle factors [4]. Identifying and quantifying the contribution of various risk factors is crucial for developing effective prevention strategies and allocating resources appropriately [5].

The Global Burden of Disease (GBD) study provides a comprehensive framework to assess the impact of breast cancer across different regions and time periods [6]. The aim of this study was to describe the influence of geographical location, social-development index (SDI), age, and gender on the global trends in the incident cases, deaths, and DALYs of BC based on data from the GBD from 1990 to 2021 in 204 countries and territories.

## Methods

### Data source and disease definition

The GBD study, accessible through the GHDx online platform (https://vizhub.healthdata.org/gbd-results/), served as the primary data source for this research on BC burden. This comprehensive initiative synthesizes epidemiological information from 204 countries and territories, offering comparative analyses of health losses attributed to 369 medical conditions and 88 risk factors. The methodological framework for data acquisition, processing, and analysis in the GBD 2021 study has been extensively documented in previous publications [7, 8]. Utilizing the GBD 2021 dataset, we extracted annual statistics on BC incidence, mortality, DALYs, and their corresponding age-standardized rates (ASRs) for the period 1990–2021. BC cases were identified using the International Classification of Diseases, 10th revision (ICD-10) codes, encompassing C50-C50.629, C50.8-C50.929, Z12.3-Z12.39, Z80.3, Z85.3, and Z86.000. For this study, estimates and their corresponding 95% uncertainty intervals (UI) for the prevalence, incidence, and DALYs associated with BC were extracted from the GBD 2021 data. The calculation of DALYs attributed to BC involved the summation of years lived with disability and years of life lost (Supplementary Methods).

For GBD studies, the Institutional Review Board of the University of Washington reviewed and approved a waiver of informed consent. This work has been reported in line with the STROCSS criteria [9].

### Sociodemographic Index

The SDI quantifies a country’s or region’s development level using fertility rate, education level, and per capita income data. Ranging from 0 to 1, a higher SDI indicates greater socioeconomic development [1, 2]. The SDI is known to correlate with disease incidence and mortality rates. In this study, we classified countries and regions into five SDI categories (low, low-medium, medium, medium–high, and high) to examine the relationship between BC burden and socioeconomic development (Supplementary Methods).

### Risk factors

Our study extends beyond the primary metrics of incidence, mortality, and DALYs to examine the impact of specific risk factors on BC burden. We focused on key factors identified in the GBD 2021 study: high body mass index, alcohol consumption, elevated fasting plasma glucose, and tobacco use. Our analysis incorporated data on BC-related DALYs and deaths attributable to these factors, stratified by region to elucidate geographical variations. To quantify the influence of these risk factors, we employed advanced methodologies, including DisMod-MR 2.1 and spatiotemporal Gaussian process regression [1]. These approaches allowed us to model exposure distributions across various demographics and locations. We established the theoretical minimum risk exposure level (TMREL) for each factor based on epidemiological evidence, representing the optimal exposure level for minimizing BC risk. By integrating exposure data, relative risk estimates, and TMRELs, we calculated population attributable fractions (PAFs) for each risk factor. These PAFs, stratified by location, age, sex, and year, quantify the potential reduction in BC burden if exposure to a given risk factor were reduced to its TMREL. To translate these fractions into meaningful health outcomes, we multiplied the PAFs by DALYs, providing estimates of the risk-attributable burden. This comprehensive approach not only highlights the direct impact of BC but also illuminates the contribution of modifiable risk factors. It offers a more nuanced understanding of the disease's burden and potential avenues for intervention across different populations. By identifying the relative importance of various risk factors in different regions, our study provides valuable insights for tailoring prevention strategies and public health initiatives to specific contexts.

### Statistical analysis

In 2021, an extensive analysis was performed to evaluate the national burden of BC, encompassing its incidence, mortality, and DALYs. Furthermore, the study explored the sociodemographic factors influencing BC's impact, examining the distribution of the disease's burden across various age cohorts and between sexes. The temporal patterns of BC incidence, DALYs, and mortality were quantified using ASRs, DALYs, and estimated annual percentage changes (EAPCs). The ASR was computed per 100,000 individuals utilizing the subsequent formula:

$$\mathrm{ASR}=\frac{\sum_{i=1}^Aa_iw_i}{\sum_{i=1}^Aw_i}\times100,00$$

($\alpha_i$: the age-specific rate in *i*^th^the age group; w: the number of people in the corresponding *i*^th^ age group among the standard population; *A*: the number of age groups)

The EAPC serves as a prevalent metric in epidemiological studies to ascertain temporal evolutions in ASRs of diseases. The coefficient, denoted as $\upbeta$, is derived from the natural logarithm of the ASRs. Herein, y represents ln(ASR) while x corresponds to the calendar years. The EAPC, accompanied by its 95% confidence interval (CI), was determined utilizing the ensuing linear regression model:

$$\text{y}=\alpha +\upbeta x+\upvarepsilon\text{EAPC}=100*(\text{exp}(\upbeta)-1)$$

An upward trend is indicated when the lower limit of the 95% CIs exceeds 0, while a downward trend is suggested when the upper limit falls below 0. If the 95% CIs encompass 0, it signifies no statistically significant variation in trend patterns.

In this study, we utilized a Bayesian age-period-cohort (BAPC) model incorporating integrated nested Laplace approximations to project future trends in BC burden. Previous research has demonstrated that BAPC offers superior coverage and precision compared to alternative prediction methods [10–13]. The computational process was implemented using the R-package BAPC, following established protocols from prior studies [10]. Additionally, risk factors for BC were assessed. All analytical procedures and graphical representations were executed using the World Health Organization's Health Equity Assessment Toolkit and the R statistical computing environment (version 4.2.1).

## Results

### Breast cancer incidence burden

**Fig. 1:**
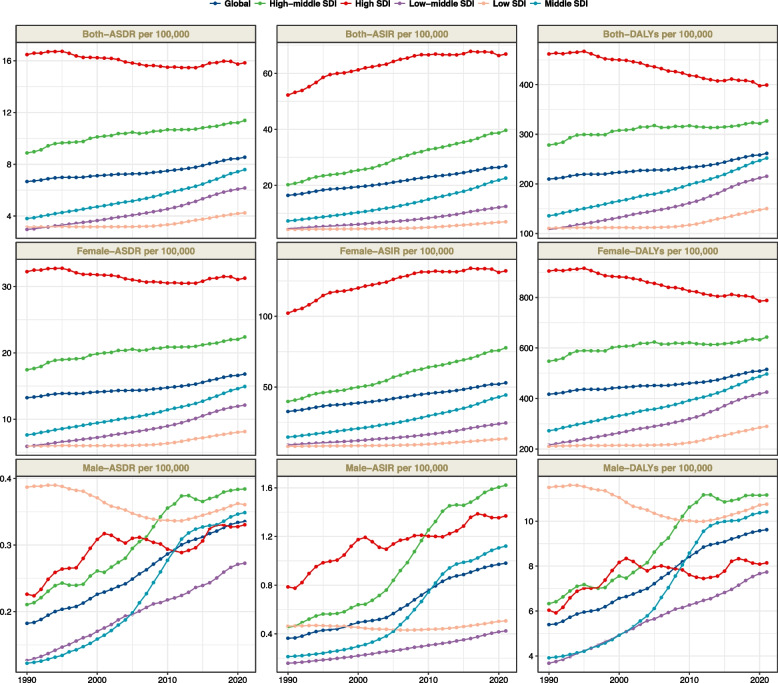
Trends in breast cancer incidence, deaths and disability-adjusted life-years from 1990 to 2021

Globally, the number of BC incident cases increased substantially from 875,657.23 in 1990 to 2,121,564.32 in 2021. The age-standardized incidence rate (ASIR) rose from 16.42 per 100,000 in 1990 to 26.88 per 100,000 in 2021. The EAPC was 1.57 (95% CI: 1.54-1.60), indicating a significant upward trend (Table 1, Fig. 1). In addition, we found that BC incidence increased in all five SDI regions, with the highest number of BC incident cases in the High SDI region at 731761.8 (95% UI: 668800.23-764115.63). In 2021, the ASIR was highest in the High SDI region (66.89 per 100,000; 95% UI, 61.13-69.84); it was lowest in the Low SDI region (6.99 per 100,000; 95% UI, 6.17-7.84) (Table 1). Low-middle SDI region has the largest increase in cases compared to 1990 (3.5-fold increase, EAPC:3.31; 95%CI: 3.23-3.39).

**Table 1:** Incidence of Breast Cancer Between 1990 and 2021 at the Global and Regional Level

**Location**	**1990**		**2021**		**EAPC_95%CI**
	**Num**ber (95%UI)	**ASR **(95%UI)	**Num**ber (95%UI)	**ASR **(95%UI)	
Global	875657.23 (834228.9-910528.91)	16.42 (15.64-17.07)	2121564.32 (1982142.64-2268722.63)	26.88 (25.12-28.75)	1.57 (1.54-1.6)
High SDI	459470.99 (438294.58-470351.64)	52.24 (49.83-53.48)	731761.8 (668800.23-764115.63)	66.89 (61.13-69.84)	0.77 (0.65-0.88)
High-middle SDI	215082.12 (204492.88-225116.75)	20.22 (19.23-21.17)	517073.04 (468473.32-576713.5)	39.65 (35.93-44.23)	2.2 (2.13-2.26)
Middle SDI	125574.83 (115589.69-137898.83)	7.29 (6.71-8)	552881.05 (499159.06-612199.33)	22.58 (20.39-25)	3.72 (3.68-3.76)
Low-middle SDI	53158.38 (47755.08-60119.41)	4.58 (4.11-5.18)	239539.85 (216754.66-261136.06)	12.47 (11.28-13.59)	3.31 (3.23-3.39)
Low SDI	21284.04 (18204.71-24812.32)	4.25 (3.63-4.95)	78125.04 (68896.18-87591.08)	6.99 (6.17-7.84)	1.55 (1.32-1.77)
Andean Latin America	2359.19 (2024.61-2733.87)	6.21 (5.33-7.2)	10899.04 (8536.63-13856.29)	16.48 (12.91-20.95)	3.11 (2.97-3.24)
Australasia	9901.3 (9367.96-10426.16)	48.83 (46.2-51.42)	19849.49 (17622.8-22084.21)	64.11 (56.92-71.33)	0.87 (0.66-1.08)
Caribbean	6073.07 (5683.34-6495.2)	17.21 (16.1-18.4)	15175.35 (12898.05-17380.17)	31.98 (27.18-36.62)	2.12 (2.03-2.22)
Central Asia	9834.11 (9253.14-10330.67)	14.19 (13.35-14.9)	15435.48 (13775.72-17276.33)	16.11 (14.38-18.03)	0.73 (0.64-0.82)
Central Europe	36396.14 (34940.06-37891.29)	29.1 (27.93-30.29)	65494.45 (60227.77-70801.69)	56.82 (52.25-61.43)	2.14 (1.98-2.3)
Central Latin America	15027.14 (14560.6-15451.98)	9.14 (8.86-9.4)	77199.72 (67262.72-87272.51)	30.51 (26.59-34.49)	3.78 (3.69-3.88)
Central Sub-Saharan Africa	2513.35 (1781.65-3388.55)	4.57 (3.24-6.17)	10096.68 (7539.77-13198.36)	7.37 (5.51-9.64)	1.54 (1.25-1.82)
East Asia	90563.29 (73963.17-109010.53)	7.44 (6.08-8.95)	418197.81 (327546.39-520456.13)	28.4 (22.24-35.34)	4.58 (4.47-4.7)
Eastern Europe	65301.21 (63263.17-67227.01)	28.83 (27.93-29.68)	101094.34 (90961.95-112243.66)	48.89 (43.99-54.29)	1.39 (1.27-1.5)
Eastern Sub-Saharan Africa	9452.65 (7918.36-11392.62)	4.95 (4.15-5.97)	34526.99 (29176.43-40787.14)	8.1 (6.85-9.57)	1.46 (1.22-1.71)
High-income Asia Pacific	31392.13 (29719.31-32940.91)	18.11 (17.14-19)	97151.24 (85263.6-105261.87)	52.39 (45.98-56.76)	3.58 (3.34-3.81)
High-income North America	221968.99 (210889.84-228569.12)	78.88 (74.94-81.22)	298017.84 (274478.89-311947.64)	80.51 (74.15-84.27)	-0.1 (-0.17--0.02)
North Africa and Middle East	16575.37 (14797.19-18934.68)	4.89 (4.36-5.58)	128349.01 (114486.75-144654.43)	20.6 (18.38-23.22)	5.2 (4.99-5.4)
Oceania	545.96 (428.4-682.09)	8.34 (6.54-10.41)	1572.5 (1284.07-1968.18)	11.29 (9.22-14.13)	0.85 (0.68-1.01)
South Asia	44568.22 (39722.15-50196.75)	4.08 (3.63-4.59)	209424.51 (183326.24-238852.89)	11.34 (9.93-12.93)	3.32 (3.12-3.52)
Southeast Asia	32674.97 (27649.4-39223.17)	7.02 (5.94-8.43)	139582.52 (116509.87-168515.66)	19.99 (16.68-24.13)	3.44 (3.38-3.5)
Southern Latin America	12152.72 (11475.92-12774.1)	24.53 (23.17-25.79)	22973.33 (21143.58-24782.87)	33.94 (31.23-36.61)	1.09 (0.95-1.22)
Southern Sub-Saharan Africa	4285.05 (3608.42-4980.58)	8.17 (6.88-9.5)	15278.16 (13703.69-16887.7)	19.03 (17.06-21.03)	3.29 (3.05-3.53)
Tropical Latin America	16308.06 (15694.6-16917.85)	10.69 (10.29-11.09)	62495.39 (58527.35-66094.95)	27.47 (25.72-29.05)	2.85 (2.76-2.94)
Western Europe	237563.23 (226213.81-244509.77)	61.8 (58.85-63.61)	335270.52 (302442.06-355611.45)	76.65 (69.15-81.3)	0.75 (0.57-0.92)
Western Sub-Saharan Africa	10201.07 (8311.13-12096.68)	5.28 (4.3-6.26)	43479.95 (32854.22-57197.9)	8.88 (6.71-11.68)	1.73 (1.52-1.95)

**Fig. 2:**
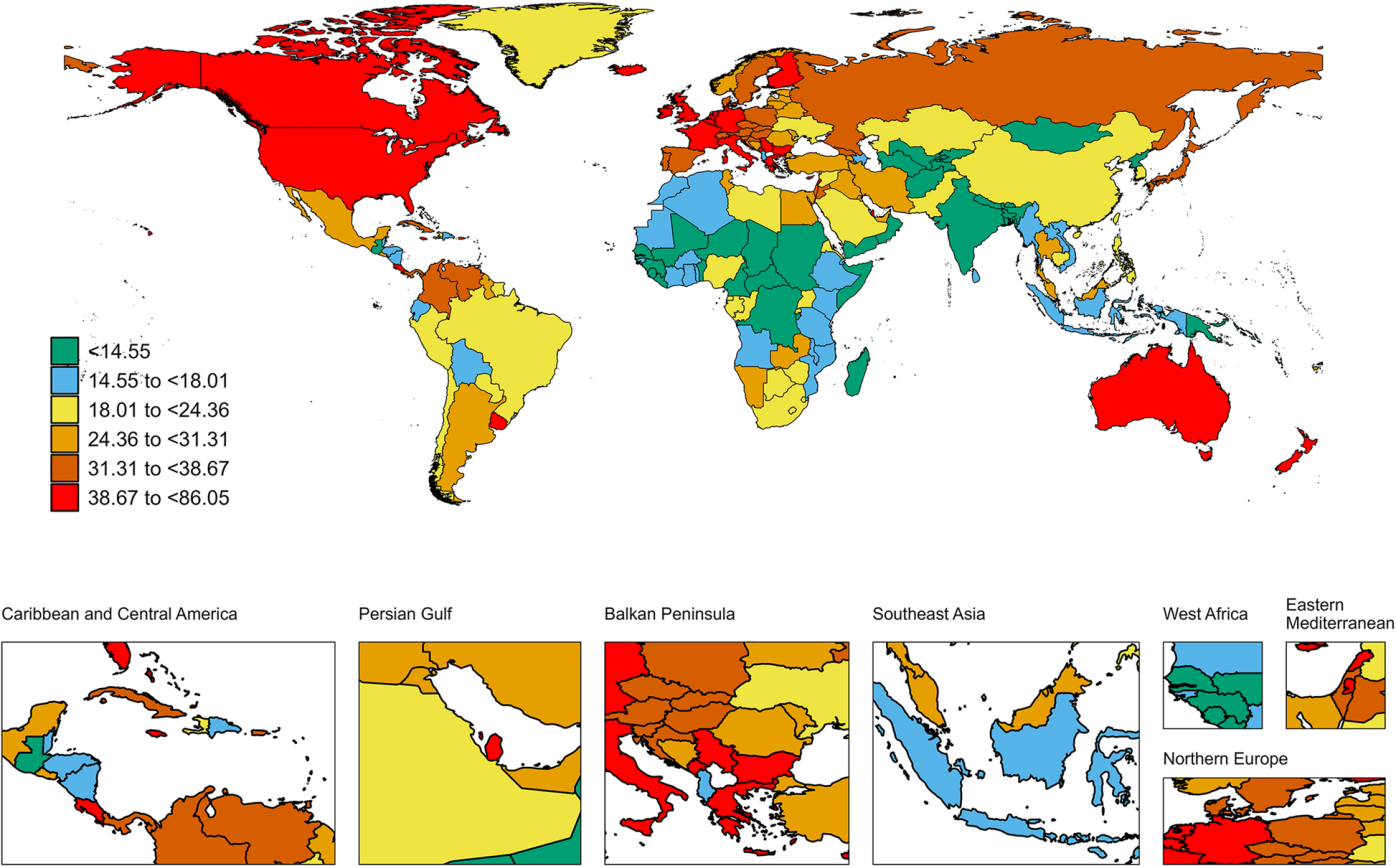
The global disease burden of breast cancer incidence rate for both sexes in 204 countries and territories

Regionally, the incidences increased in all 21 GBD regions between 1990 and 2021 with the largest increases in North Africa and Middle East (EAPC: 5.2; 95%CI: 4.99-5.4) and the lowest increases in High-income North America (EAPC: -0.1; 95%CI: -0.17 to -0.02) (Table 1). The ASIR was highest in High-income North America, at 80.51 per 100,000 persons (95% UI: 74.15-84.27), followed by Western Europe, at 76.65 per 100,000 persons (95% UI: 69.15-81.3). By contrast, low-income regions demonstrated significantly lower ASIRs, including Western Sub-Saharan Africa at 8.88 per 100,000 persons (95% UI: 6.71-11.68), Eastern Sub-Saharan Africa at 8.1 per 100,000 persons (95% UI: 6.85-9.57), Central Sub-Saharan Africa at 7.37 per 100,000 persons (95% UI: 5.51-9.64) (Table 1). China, the USA, and India were the 3 countries with the highest reported new cases of BC in 2021 while Nauru, Niue and Tokelau were the 3 countries with the least. Monaco, Bermuda, and France showed the highest ASIR while Somalia,Chad and Niger showed the lowest ASIR in 2021 (Fig. 2, TableS1).

A significant positive relationship was found between the SDI and the ASIR (R: 0.71, *p *< 0.001), suggesting that BC incidence is higher in more economically developed countries (Fig. S1). The relationship between ASIR and SDI for each of the 21 Global GBD regions is shown in Fig. S2. The ASIR tends to be numerically higher in regions with a higher SDI compared to those with a lower SDI.

### Breast cancer deaths and DALY burden

In 2021, the worldwide number of deaths cases was 674199.41 (95% UI: 623371.55-720822.55) (TableS2), with an age-standardized deaths rates (ASDR) of 8.54 per 100,000 persons (95% UI: 7.9-9.13), showing a decrease of 1.88 per 100,000 persons from 1990 to 2021. The global DALYs for BC in 2021 was 20635718.18 (95% UI: 19358110.66-21993502.55) (Fig. 1; Table S3), with an age-standardized DALYs rates of 261.5 per 100,000 persons (95% UI: 245.31-278.7), which decreased by 51.86 between 1990 and 2021 (Fig. 1; Table S3). Among the five SDI regions, the High SDI region had the highest ASDR at 15.84 (95% UI: 13.99-16.81) and the highest age-standardized DALY rate at 399.21 (95% UI: 367.83-425.21). Notably, the High SDI region was the only one among the five SDI regions where both the ASDR and age-standardized DALY rate decreased over time, with an EAPC of -0.07 (95% CI: -0.14 to -0.01) for ASDR and -0.56 (95% CI: -0.59 to -0.52) for the DALY rate.

Among the five SDI regions, the Low-middle SDI region experienced the greatest increases in both ASDR and age-standardized DALY rate, with an EAPC of 2.24 (95% CI: 2.16 to 2.31) for ASDR and 2.27 (95% CI: 2.19 to 2.34) for the DALY rate.

**Fig. 3:**
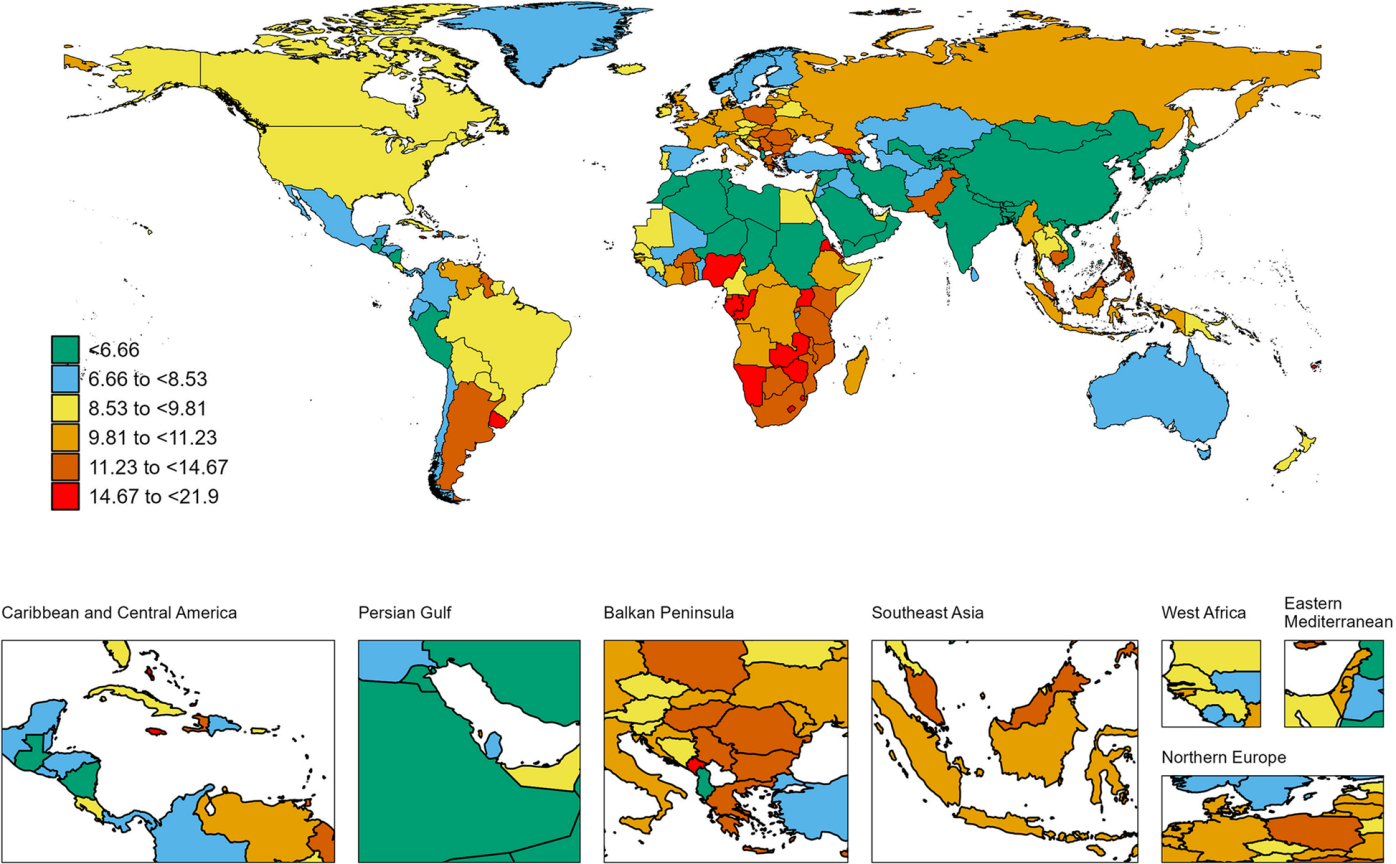
The global disease burden of breast cancer death rate for both sexes in 204 countries and territories

Regionally, South Asia had the highest number of BC deaths throughout the study period, reaching 108,084.94 (95% UI: 94,490.36-123,378.98) cases in 2021. Meanwhile, High-income Asia Pacific showed the largest increase in BC deaths between 1990 and 2021, with an EAPC of 2.98 (95% CI: 2.91-3.06). Geographically, the ASDR was highest in Central Europe at 21.88 per 100,000 persons (95% UI: 19.93-23.72), followed by Western Europe at 21.46 per 100,000 persons (95% UI: 18.45-23.13). Among the 21 GBD regions, Central Asia, Western Europe, Australasia, and High-income North America showed a declining trend in ASDR. In 2021,China had the highest number of BC-associated deaths (91483.8; 95% UI, 71738.6-113710.5) (Table S2, Fig. 3). Monaco (46.2; 95% UI, 35.2-60.7) had the highest ASDR; Oman (1; 95% CI, 0.8-1.3) had the lowest ASDR (Table S2, Fig. 3). Turkey (EAPC, 4.74; 95% CI, 4.18-5.29) had the greatest increases in the mortality rate; the Afghanistan (EAPC, -0.61; 95% CI, -0.92–0.29) had the greatest decreases.

**Fig. 4:**
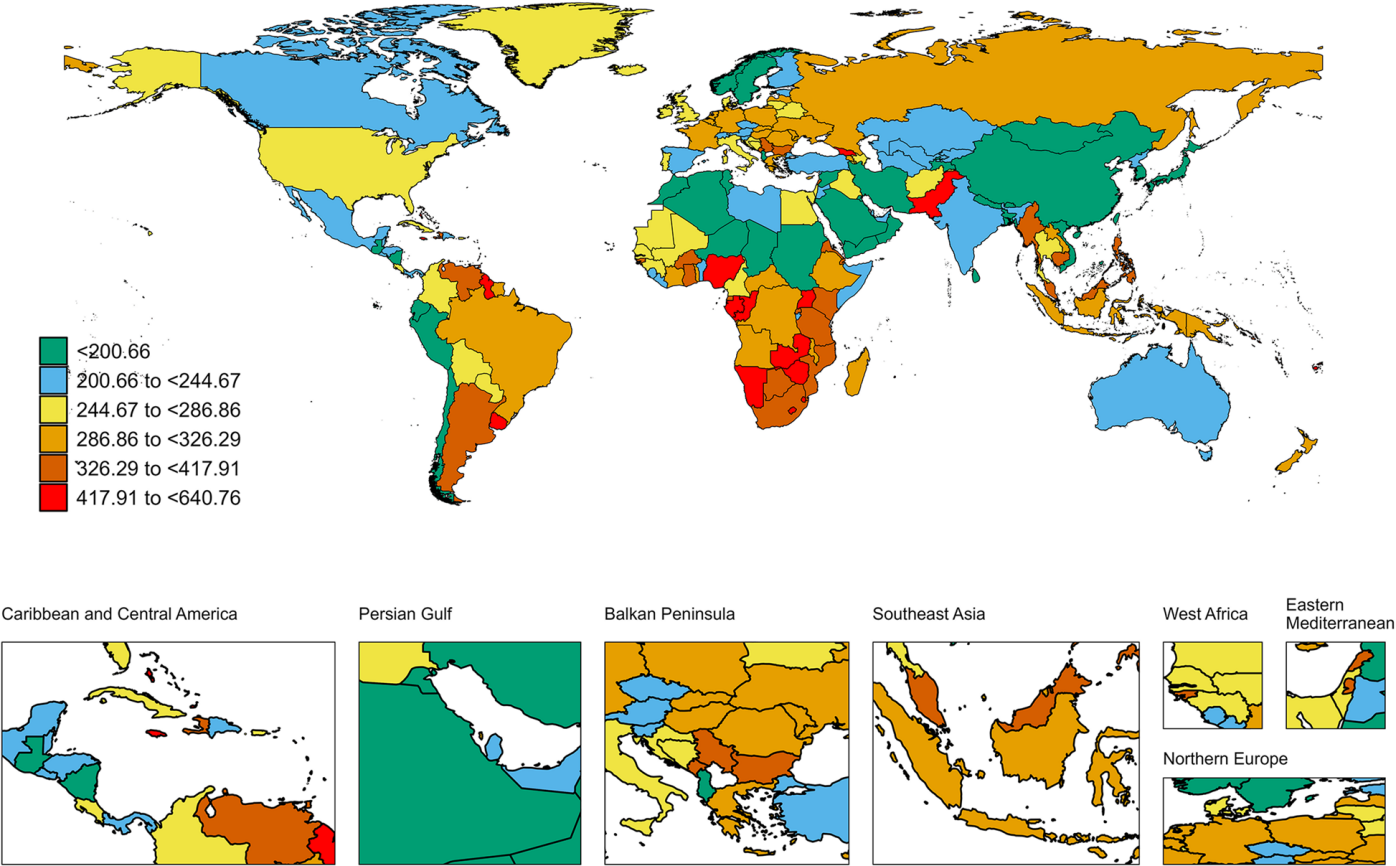
The global disease burden of breast cancer DALYs rate for both sexes in 204 countries and territories

The age-standardized DALY rate increased in 15 regions, while it decreased in 6 GBD regions. The highest age-standardized DALY rates were observed in Central Europe (529.03; 95% UI: 488.00-572.35), Western Europe (491.05; 95% UI: 443.23-528.69), and Eastern Europe (479.03; 95% UI: 427.68-543.23) (Fig. 4). South Asia had the highest number of DALY cases (3,781,141.03; 95% UI: 3,308,591.50-4,332,368.78). North Africa and the Middle East showed the largest increase in the age-standardized DALY rate for breast cancer between 1990 and 2021, with an EAPC of 3.08 (95% CI: 2.90-3.26). In 2021, China had the highest number of DALYs (3029404.7; 95% UI, 2360641.2-3844035.9). (TableS3). Nauru had the highest rate of DALYs (640.8; 95% UI, 381.9-994.5) (TableS3, Fig. 4). Turkey (EAPC, 3.81; 95% CI, 3.09-4.54) had the greatest increase in DALYs rate; Denmark (EAPC, -2.76; 95% CI,-2.88–2.65) had the greatest decreases (TableS3).

### Age and sex patterns

**Fig. 5:**
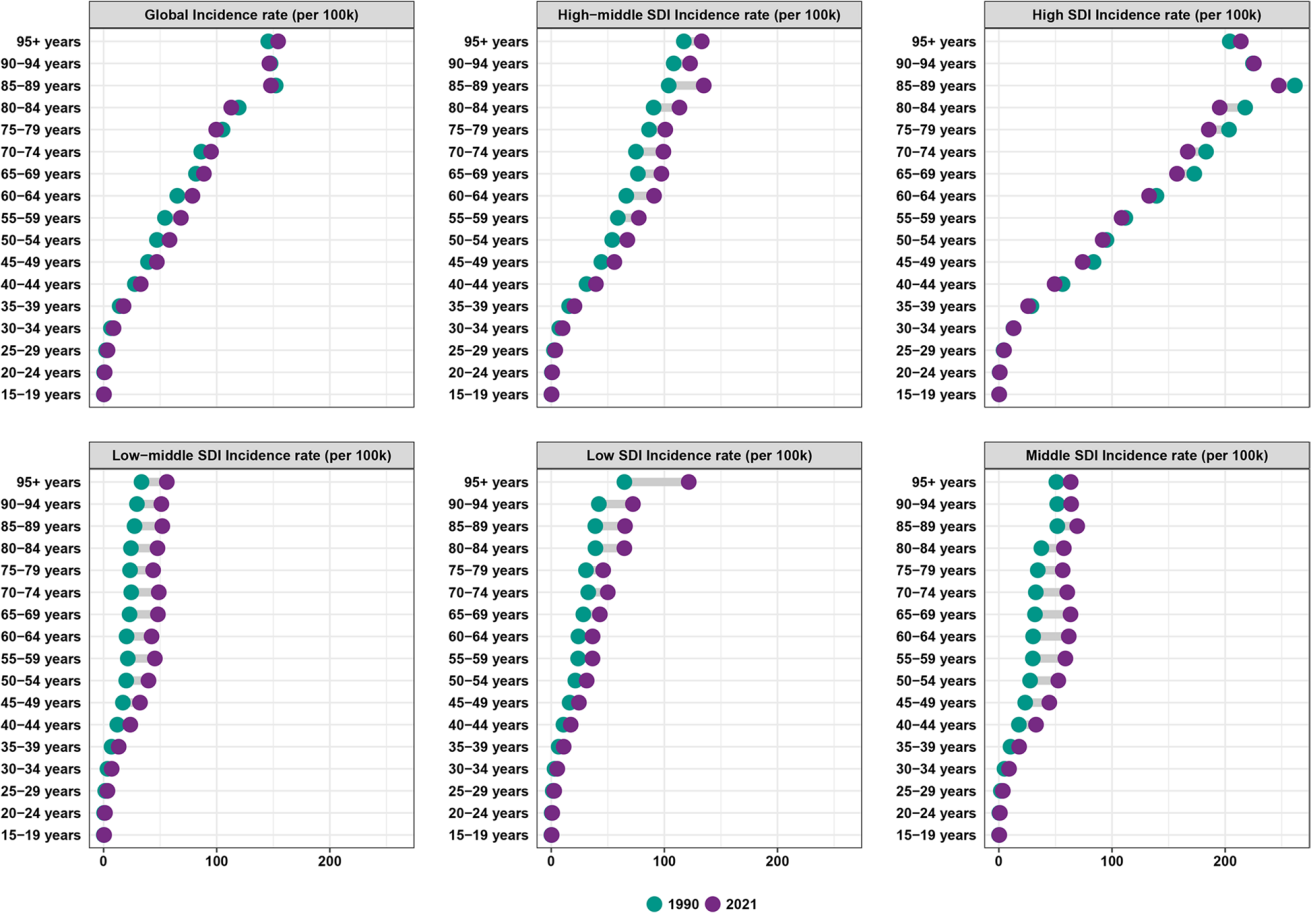
Breast cancer incidence by age group, global and 5 SDI regions

In 2021, the highest global incidence rates of BC were observed in individuals aged 95 and older, with incidence rates increasing with advancing age.Globally, we found that compared to 1990, BC incidence rate in 2021 increased in the 40-74 year age group, remained relatively unchanged in the 15-39 year age group, and decreased in the 80-89 year age group (Fig. 5). In most of the five SDI regions, BC incidence increased across all age groups compared to 1990. However, in the High SDI region, incidence rates were lower in 2021 than in 1990 for the 15-89 year age group. BC incidence rates in High SDI regions are generally higher than in other regions, particularly for age groups older than 40 years. Starting from the age of 40, the incidence in High SDI regions is significantly higher compared to other regions and increases rapidly with advancing age. Overall, BC incidence was significantly higher in women than in men. However, we also found a significant acceleration in BC incidence among men in regions with High SDI, High-middle SDI, and Middle SDI (Fig. 1). In terms of mortality, we found a decrease in BC mortality rates worldwide compared to 1990 (Fig. S3). The highest mortality rate was observed in the over-95 age group, with an overall increase in mortality rates with advancing age. Over the 32-year period, mortality rates declined the most in High SDI regions. In contrast, mortality rates in less developed regions increased compared to 1990 (Fig. S3). The same trend was observed in DALYs (Fig. S4). The highest DALY rates were found in the over-95 age group, with an overall increase in DALY rates as age advanced. High SDI regions experienced the greatest reduction in DALY rates over the 32-year period. However, less developed regions showed an increase in DALY rates compared to 1990. This suggests that while the overall global burden of breast cancer has decreased since 1990, disparities exist between regions with different levels of socioeconomic development.

### Risk factors

**Fig. 6:**
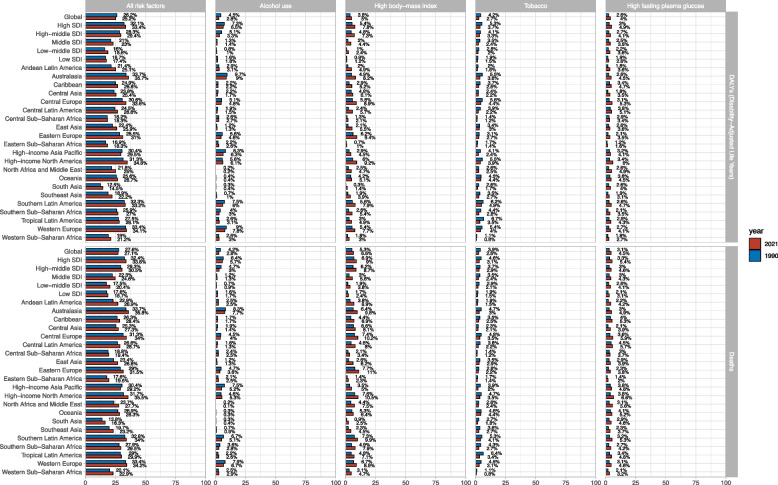
The breast cancer DALYs and deaths attributable to risk factors compared in 1990 and 2021, globally and by region

Globally, high body-mass index was the greatest contributor to DALYs. Other significant contributors included alcohol use, tobacco, and high fasting plasma glucose (Fig. 6). However, in Australasia, Western Europe, and High-income Asia Pacific, alcohol use was the greatest contributor to DALYs. BC DALYs attributable to high body-mass index varied by SDI quintiles in 2021. Globally, 5% of BC DALYs were attributable to high body-mass index. In the High SDI quintile, 7.8% of BC DALYs were attributable to high body-mass index, while in the Low SDI quintile, only 1.2% of BC DALYs were attributable to high body-mass index. In 2021, 2.7% of BC DALYs were attributable to tobacco, showing a decrease from previous years. In the High SDI quintile, 3.7% of BC DALYs were attributable to tobacco. Across the other 21 GBD regions, the proportion of BC DALYs attributable to smoking also decreased compared to 1990 (Fig. 6). In 2021, 4% of BC DALYs were attributable to high fasting plasma glucose, showing a slight increase from previous years. In the High SDI quintile, 4.9% of BC DALYs were attributable to high fasting plasma glucose, also demonstrating a slight increase. Across the other 21 GBD regions, the proportion of BC DALYs attributable to high fasting plasma glucose also increased compared to 1990 (Fig. 6). Globally and in the High SDI, High-middle SDI, and Middle SDI quintiles, high body-mass index was the greatest contributor to BC deaths. However, in the Low SDI quintile, high fasting plasma glucose was the greatest contributor to BC deaths. Overall, BC deaths from smoking and alcohol use decreased compared to 1990, while High body-mass index and High fasting plasma glucose increased compared to 1990 (Fig. 6).

### Future forecasts of global burden of breast cancer

Fig.S5 shows the future forecasts of the GBD study for BC incidence. As illustrated in Fig. S5, the ASIR of BC worldwide is expected to remain relatively stable, with a projected ASIR of 24.53 per 100,000 in 2030. The ASIR for females is projected to reach 46.30 per 100,000. However, on a global scale, the absolute number of new BC cases is expected to continue increasing. Over time, the ASDR of BC in females decreased slightly and is projected to reach 19.59 per 100,000 in 2030 (Fig. S6). It is estimated that by 2030, the ASR of female BC DALYs will be 450.20/100,000; the ASR of BC DALYs in male will be 8.97 cases per 100,000 (Fig. S7).

## Discussion

Our study provides a comprehensive analysis of the global burden of BC from 1990 to 2021, revealing significant disparities across regions with varying SDI. The findings demonstrate a substantial increase in BC incidence worldwide, with the number of cases more than doubling over the study period. This trend aligns with previous studies that have reported rising breast cancer incidence globally [14, 15].

The global ASIR increased from 16.42 per 100,000 in 1990 to 26.88 per 100,000 in 2021, with an EAPC of 1.57. This significant upward trend was observed across all SDI regions, with the highest ASIR in 2021 found in the High SDI region (66.89 per 100,000) and the lowest in the Low SDI region (6.99 per 100,000). These findings underscore the substantial disparities in BC incidence between regions of different socioeconomic development. The observed increase in BC incidence can be attributed to several factors. Improved screening programs and diagnostic techniques in many countries have led to earlier and more frequent detection of BC [16]. For instance, the widespread adoption of mammography screening in high-income countries has contributed to increased detection rates [17]. Similarly, the gradual implementation of screening programs in middle-income countries has contributed to rising incidence rates in these regions. Shifts in reproductive behaviors, particularly in high SDI regions, have been associated with increased BC risk. These changes include delayed childbearing, with the trend towards having first children at later ages linked to increased breast cancer risk [18]. Reduced parity, with women having fewer children or no children at all, is associated with a higher risk of BC. Decreased duration of breastfeeding, especially in high-income countries, may also contribute to increased risk. The globalization of Western lifestyles, especially in developing countries, has been implicated in the rising incidence of BC. Key aspects of this lifestyle shift include dietary changes, with increased consumption of processed foods, animal products, and foods high in saturated fats associated with higher BC risk. Reduced physical activity due to urbanization and technological advancements has led to more sedentary lifestyles, which is a known risk factor for BC [19, 20]. Rising alcohol intake, particularly in women, has been linked to increased BC risk. The global increase in obesity rates, especially in middle- and high-income countries, is a significant contributor to rising BC incidence [20]. Obesity, particularly post-menopausal obesity, is a well-established risk factor for BC due to its effects on estrogen levels and inflammation. Exposure to environmental pollutants and endocrine-disrupting chemicals may also play a role in the increasing incidence of BC, although more research is needed to fully understand these associations. As global life expectancy has increased, more women are living to ages where BC risk is highest, contributing to the overall increase in incidence rates. The stark disparity in ASIR between High and Low SDI regions (66.89 vs 6.99 per 100,000 in 2021) reflects not only differences in risk factor profiles but also inequalities in healthcare access and quality. In low SDI regions, lower incidence rates may partially reflect underdiagnosis due to limited screening programs, inadequate healthcare infrastructure, and lower awareness. As these regions develop economically and adopt more Westernized lifestyles, they may face a "cancer transition," with increasing BC incidence rates. Understanding these multifaceted contributors to the global increase in BC incidence is crucial for developing targeted prevention strategies and allocating resources effectively. It highlights the need for comprehensive approaches that address modifiable risk factors, improve screening and early detection programs, and ensure equitable access to quality healthcare across all SDI regions. Furthermore, it underscores the importance of tailoring interventions to the specific needs and circumstances of different populations and regions to effectively combat the rising global burden of BC.

Regionally, we observed significant variations in BC incidence. North Africa and the Middle East showed the largest increase (EAPC: 5.2), while High-income North America experienced a slight decrease (EAPC: -0.1). These regional differences highlight the complex interplay of factors influencing BC incidence, including genetics, environmental exposures, and healthcare access. Our analysis of age patterns revealed that the highest global incidence rates of BC were observed in individuals aged 95 and older, with incidence rates generally increasing with advancing age. Compared to 1990, we found increased incidence rates in the 40–74 year age group in 2021, while rates remained relatively unchanged in the 15–39 year age group and decreased in the 80–89 year age group. These age-specific trends provide valuable insights for targeted screening and prevention strategies.

The study also highlighted significant gender disparities, with BC incidence being significantly higher in women than in men. This finding aligns with the well-established understanding that BC predominantly affects women, primarily due to biological factors such as hormonal influences and breast tissue composition. The female breast is more susceptible to carcinogenic changes due to its complex structure and cyclical hormonal fluctuations throughout a woman's life. Estrogen and progesterone, hormones that play crucial roles in female reproductive physiology, are also known to influence the development and progression of BC. These hormonal factors, combined with genetic predispositions and environmental influences, contribute to the higher incidence of BC in women. However, we observed a notable acceleration in BC incidence among men in regions with High SDI, High-middle SDI, and Middle SDI. This trend is particularly intriguing and raises several important questions about the changing landscape of male BC. While male BC remains relatively rare, accounting for less than 1% of all BC cases globally, the observed acceleration in these specific SDI regions suggests potential shifts in risk factors or detection practices. Several factors could contribute to this trend. Improved awareness and screening practices in higher SDI regions may lead to increased detection of male BC cases that might have gone undiagnosed in the past. Additionally, changes in lifestyle factors such as increased obesity rates, which is a known risk factor for male BC, could play a role in the rising incidence. Environmental exposures to endocrine-disrupting chemicals, which are more prevalent in industrialized regions, might also contribute to this trend. Furthermore, the aging population in higher SDI regions could be a factor, as the risk of BC in men, like in women, increases with age. This trend warrants further investigation to elucidate the specific factors driving the acceleration of male BC in these regions. Such research could provide valuable insights into the etiology of male BC and potentially reveal new risk factors or mechanisms of disease development.

Our analysis of risk factors reveals that high body-mass index is the greatest contributor to BC DALYs globally, followed by alcohol use, tobacco, and high fasting plasma glucose. In 2021, 5% of breast cancer DALYs were attributable to high body-mass index globally, with this proportion rising to 7.8% in the High SDI quintile. These findings underscore the importance of addressing modifiable risk factors in BC prevention strategies. The variation in risk factor contributions across SDI regions highlights the need for tailored interventions that consider local contexts and resources. The observed decrease in BC mortality rates in high SDI regions is encouraging and likely reflects advancements in treatment and early detection. The global ASDR decreased from 10.42 per 100,000 in 1990 to 8.54 per 100,000 in 2021. However, the persistent and increasing mortality rates in lower SDI regions emphasize the urgent need for improved access to quality healthcare and cancer control programs in these areas. Similarly, the global age-standardized DALY rate decreased from 313.36 per 100,000 in 1990 to 261.5 per 100,000 in 2021. The High SDI region was the only one among the five SDI regions where both the ASDR and age-standardized DALY rate decreased over time. This trend highlights the impact of advanced healthcare systems and effective cancer control strategies in more developed regions.

Our future forecasts suggest that while the global ASIR of breast cancer is expected to remain relatively stable (projected ASIR of 24.53 per 100,000 in 2030), the absolute number of new cases is likely to continue increasing. This projection underscores the ongoing challenge of breast cancer and the need for sustained efforts in prevention, early detection, and treatment.

### Strengths and limitations

To the best of our knowledge, this GBD-based study represents the most comprehensive effort to date to analyze the global burden of BC, including incidence, mortality, and DALYs, as well as to assess the contribution of various risk factors and project future trends. However, our study has several limitations that should be considered when interpreting the results. Firstly, the quality and availability of data vary significantly across different countries and regions. Many low- and middle-income countries lack robust population-based cancer registries, which may lead to an underestimation or inaccurate representation of the true BC burden in these areas. This data gap is particularly pronounced in rural regions and areas with limited healthcare infrastructure, potentially skewing our global estimates. Secondly, while our study includes a range of risk factors, it is not exhaustive. The GBD 2021 study provides data on several behavioral and metabolic risks, but there are other potentially important factors that we were unable to assess. For instance, genetic predisposition, environmental exposures, and certain reproductive factors are not fully captured in our analysis. This limitation may result in an incomplete understanding of the full spectrum of BC risk factors and their relative contributions to the disease burden. Thirdly, our projections of the future BC burden are based on current trends and patterns. These forecasts do not account for potential future changes in risk factors, advancements in screening and treatment technologies, or shifts in healthcare policies. As such, they should be interpreted with caution and regularly updated as new data become available. Fourthly, while DALYs provide a comprehensive measure of disease burden, incorporating both mortality and morbidity, they may not fully capture the psychosocial impact of BC on patients and their families. The long-term effects of BC survivorship, including quality of life issues and economic burden, are not completely reflected in our DALY calculations. Lastly, our study relies on the methodological framework of the GBD study, which, although robust, has its own limitations. These include potential biases in data collection and modeling approaches, as well as assumptions made in the estimation process. Furthermore, the use of disability weights in DALY calculations is based on survey data and may not perfectly reflect the lived experiences of BC patients across different cultural contexts.

Despite these limitations, our study provides valuable insights into the global landscape of BC burden and offers a foundation for future research and policy development in BC prevention and control. We acknowledge these constraints to encourage cautious interpretation of our findings and to highlight areas for improvement in future studies.

## Conclusion

In conclusion, our findings highlight the growing global burden of BC and the significant disparities between regions of different socio-economic development. The increasing incidence in lower SDI regions, coupled with persistent high rates in high SDI areas, calls for a multi-faceted approach to breast cancer control. This approach should include improving access to screening and treatment in less developed regions, implementing targeted prevention strategies, and addressing modifiable risk factors. Future research should focus on developing cost-effective interventions tailored to local contexts and resources to reduce the global burden of BC. Moreover, efforts should be made to improve data collection and quality in low-resource settings to enable more accurate monitoring of BC trends and the effectiveness of interventions.

## Supplementary Information

Supplementary Material 1: Fig. S1. The correlation between age-standardized incidence rate and SDI in 2021 across 204 countries.

Supplementary Material 2: Fig. S2. The correlation between age-standardized incidence rate and SDI in 2021 globally and by region.

Supplementary Material 3: Fig. S3. Breast cancer deaths by age group, global and 5 SDI regions.

Supplementary Material 4: Fig. S4. Breast cancer DALYs by age group, global and 5 SDI regions.

Supplementary Material 5: Fig. S5. Future Forecasts of GBD in Breast cancer incidence.

Supplementary Material 6: Fig. S6. Future Forecasts of GBD in Breast cancer deaths.

Supplementary Material 7: Fig. S7. Future Forecasts of GBD in Breast cancer DALYs.

Supplementary Material 8: Table S1. Incidence of Breast Cancer Between 1990 and 2021 at the 204 Countries Level.

Supplementary Material 9: Table S2. Deaths from Breast Cancerin 204 Countries, Globally and Regionally.

Supplementary Material 10: Table S3. Disabdeathsility-adjusted life years from Breast Cancerin 204 Countries, Globally and Regionally.

Supplementary Material 11.

## Data Availability

GBD study 2021 data resources were available online from the Global Health Data Exchange (GHDx) query tool (http://ghdx.healthdata.org/gbd-results-tool).
