# Correction: Global burden of breast cancer and attributable risk factors in 204 countries and territories, from 1990 to 2021: results from the global burden of Disease Study 2021

**DOI:** 10.1186/s40364-025-00729-7

**Published:** 2025-01-11

**Authors:** Rui Sha, Xiang‑meng Kong, Xin‑yu Li, Ya‑bing Wang

**Affiliations:** 1 Department of Thyroid and Breast Surgery, The First Affiliated Hospital of Wannan Medical College, (Yijishan Hospital of Wannan Medical College), Zheshan West Rd No. 2, Wuhu, 241001 Anhui Province China; 2 Department of Cardiology, Shanghai Ninth People’s Hospital, Shanghai Jiao Tong University School of Medicine, No.639 Zhizaoju Road, Huangpu District, Shanghai, 200011 China; 3 Department of Plastic and Reconstructive Surgery, Shanghai Ninth People’s Hospital, Shanghai Jiao Tong University School of Medicine, Shanghai, China

**Biomarker Research (2024) 12:87**

10.1186/s40364-024-00631-8

The authors wish to note the following two amendments to the original article affecting two errors:

For Error 5: The 1990 global ASDR should indeed be 6.66, not 10.42 as mistakenly written in the text. The figure in the table is correct.

For Error 7: The 1990 age-standardized DALYs rate should be 209.64 per 100,000, not 313.36 as erroneously stated in the text. Again, the figure in the table is correct.

