# Supplementary material for: Global burden of breast cancer and attributable risk factors in 204 countries and territories, from 1990 to 2021: results from the Global Burden of Disease Study 2021
Source: Biomark Res. 2024 Aug 26;12:87. doi: 10.1186/s40364-024-00631-8 (PMC11346191; doi:10.1186/s40364-024-00631-8)
Supplement: Supplementary file 1 — Supplementary Material 1: Fig. S1. The correlation between age-standardized incidence rate and SDI in 2021 across 204 countries. [file 40364_2024_631_MOESM1_ESM.pdf]

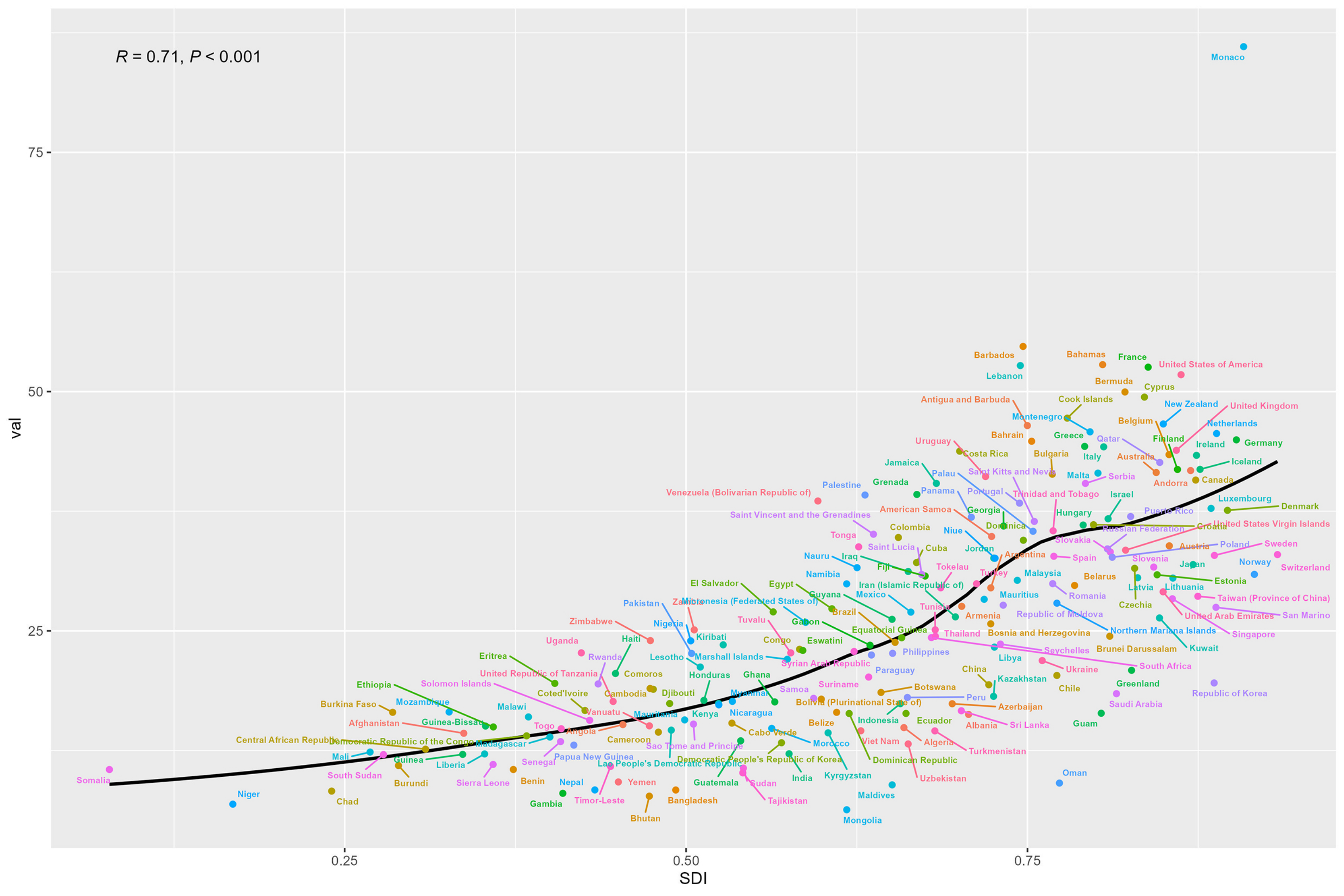

$R = 0.71$, $P < 0.001$
val
SDI
75
50
25
0.25
0.50
0.75
Monaco
Barbados
Lebanon
Bahamas
France
United States of America
Bermuda
Cyprus
Antigua and Barbuda
Cook Islands
New Zealand
United Kingdom
Montenegro
Belgium
Netherlands
Bahrain
Greece
Qatar
Finland
Ireland
Germany
Uruguay
Costa Rica
Bulgaria
Italy
Australia
Iceland
Jamaica
Palau
Saint Kitts and Nevis
Malta
Serbia
Andorra
Canada
Grenada
Palestine
Panama
Portugal
Trinidad and Tobago
Israel
Venezuela (Bolivarian Republic of)
Luxembourg
American Samoa
Georgia
Hungary
Puerto Rico
Denmark
Saint Vincent and the Grenadines
Dominica
Russian Federation
Croatia
United States Virgin Islands
Colombia
Niue
Tonga
Slovakia
Austria
Poland
Sweden
Saint Lucia
Cuba
Jordan
Argentina
Spain
Slovenia
Nauru
Iraq
Tokelau
Japan
Norway
Switzerland
Fiji
Malaysia
Belarus
Namibia
Turkey
Latvia
Estonia
Iran (Islamic Republic of)
Lithuania
El Salvador
Egypt
Guyana
Mexico
Mauritius
Romania
Taiwan (Province of China)
Pakistan
Zambia
Micronesia (Federated States of)
Tunisia
Armenia
Czechia
Brazil
Republic of Moldova
United Arab Emirates
San Marino
Zimbabwe
Tuvalu
Gabon
Nigeria
Equatorial Guinea
Thailand
Bosnia and Herzegovina
Northern Mariana Islands
Singapore
Kiribati
Congo
Eswatini
Uganda
Haiti
Brunei Darussalam
Kuwait
Rwanda
Lesotho
Marshall Islands
Philippines
Seychelles
Eritrea
Syrian Arab Republic
Libya
South Africa
United Republic of Tanzania
Comoros
Honduras
Paraguay
Ukraine
Ethiopia
Solomon Islands
Ghana
China
Chile
Kazakhstan
Greenland
Cambodia
Djibouti
Samoa
Suriname
Botswana
Republic of Korea
Coted'Ivoire
Myanmar
Peru
Saudi Arabia
Burkina Faso
Mozambique
Bolivia (Plurinational State of)
Malawi
Vanuatu
Kenya
Nicaragua
Azerbaijan
Afghanistan
Guinea-Bissau
Mauritania
Indonesia
Ecuador
Albania
Sri Lanka
Guam
Togo
Angola
Belize
Cabo Verde
Central African Republic
Democratic Republic of the Congo
Madagascar
Cameroon
Morocco
Viet Nam
Algeria
Sao Tome and Principe
Mali
Turkmenistan
Guinea
Senegal
Papua New Guinea
Democratic People's Republic of Korea
Dominican Republic
Liberia
Lao People's Democratic Republic
India
Kyrgyzstan
South Sudan
Oman
Uzbekistan
Somalia
Burundi
Sierra Leone
Benin
Nepal
Yemen
Guatemala
Sudan
Maldives
Niger
Chad
Gambia
Timor-Leste
Bangladesh
Tajikistan
Mongolia
Bhutan
