# Supplementary figures and images for: Global burden of breast cancer and attributable risk factors in 204 countries and territories, from 1990 to 2021: results from the Global Burden of Disease Study 2021
Source: Biomark Res. 2024 Aug 26;12:87. doi: 10.1186/s40364-024-00631-8 (PMC11346191; doi:10.1186/s40364-024-00631-8)

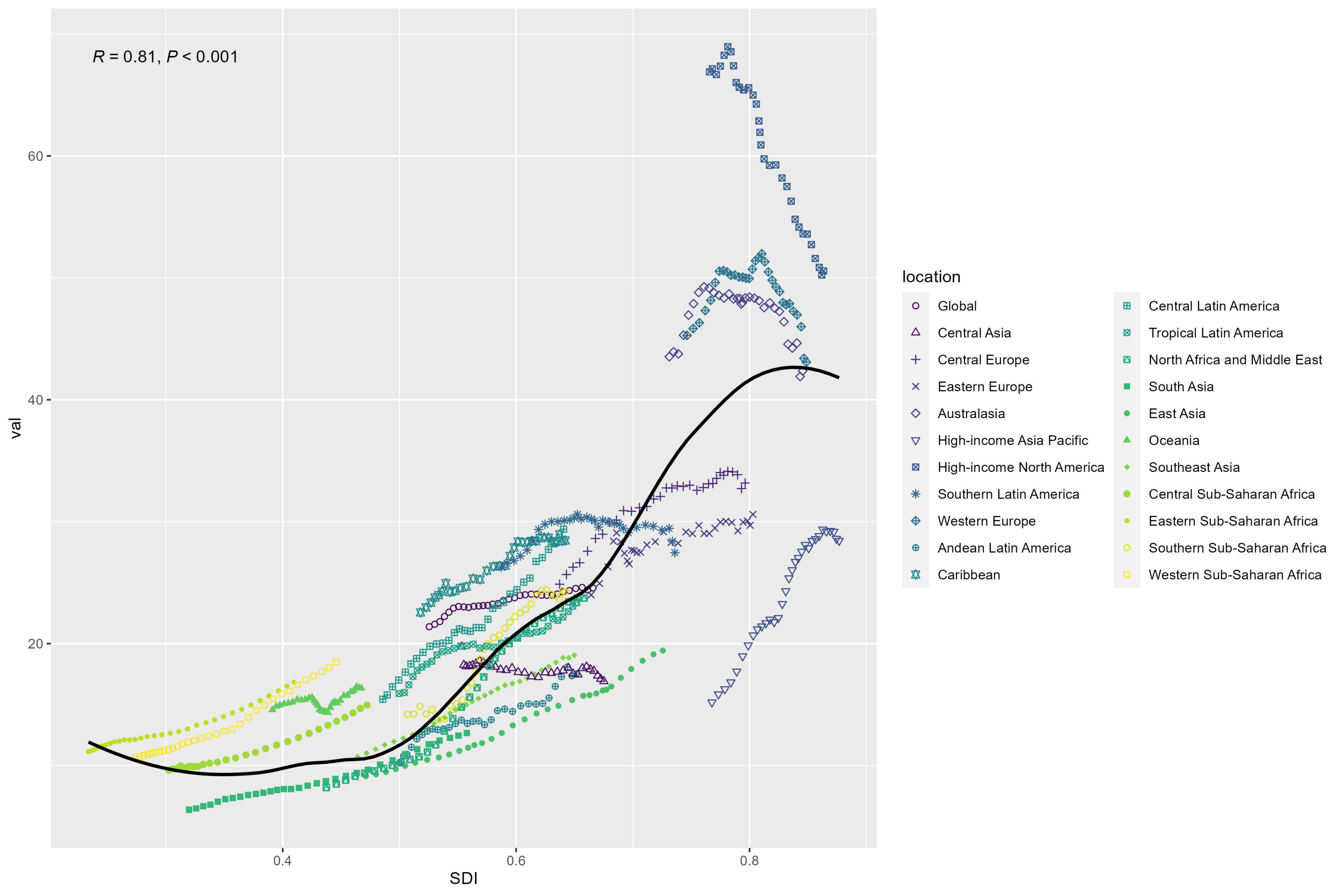

Supplement: Supplementary file 2 — Supplementary Material 2: Fig. S2. The correlation between age-standardized incidence rate and SDI in 2021 globally and by region. [file 40364_2024_631_MOESM2_ESM.pdf]

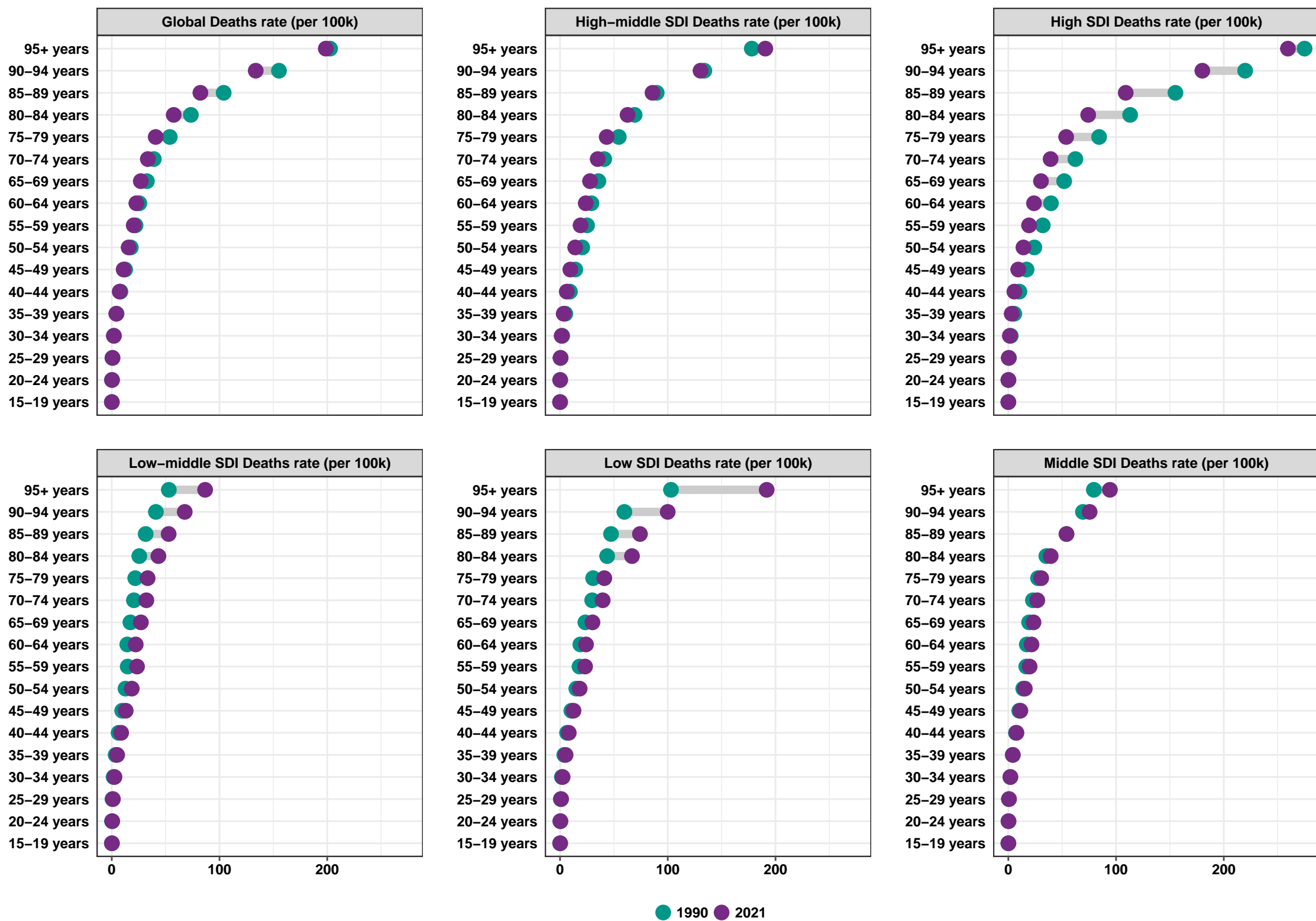

Supplement: Supplementary file 3 — Supplementary Material 3: Fig. S3. Breast cancer deaths by age group, global and 5 SDI regions. [file 40364_2024_631_MOESM3_ESM.pdf]

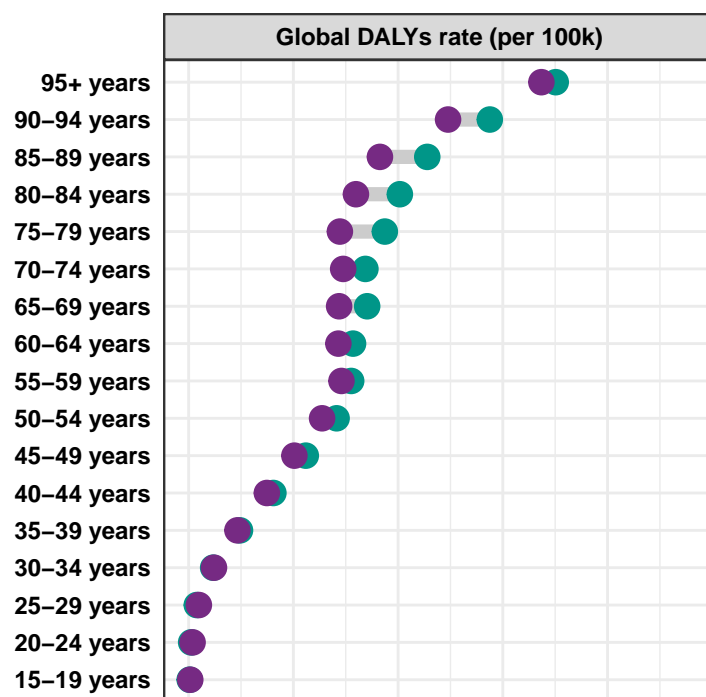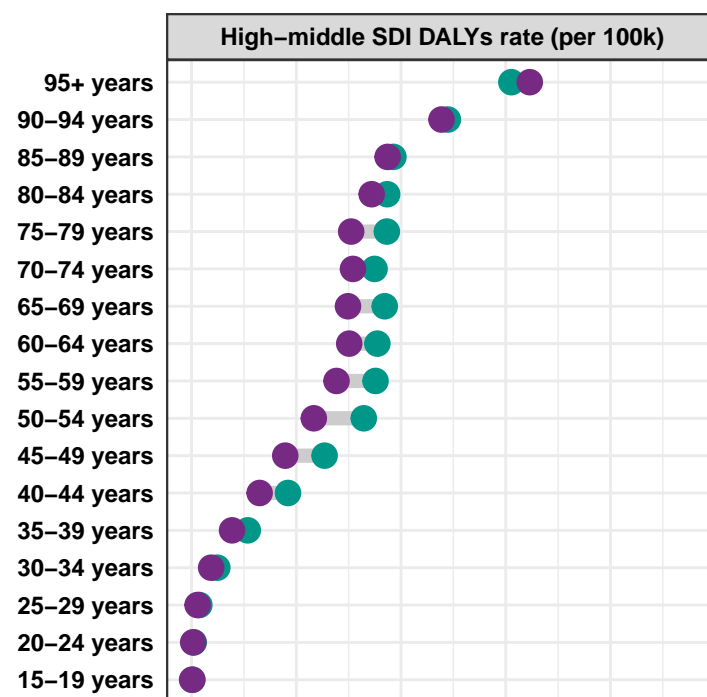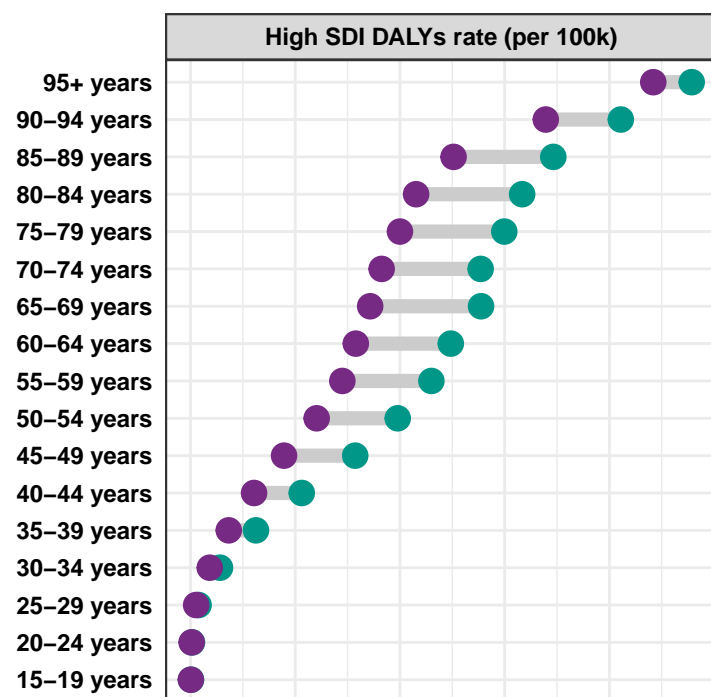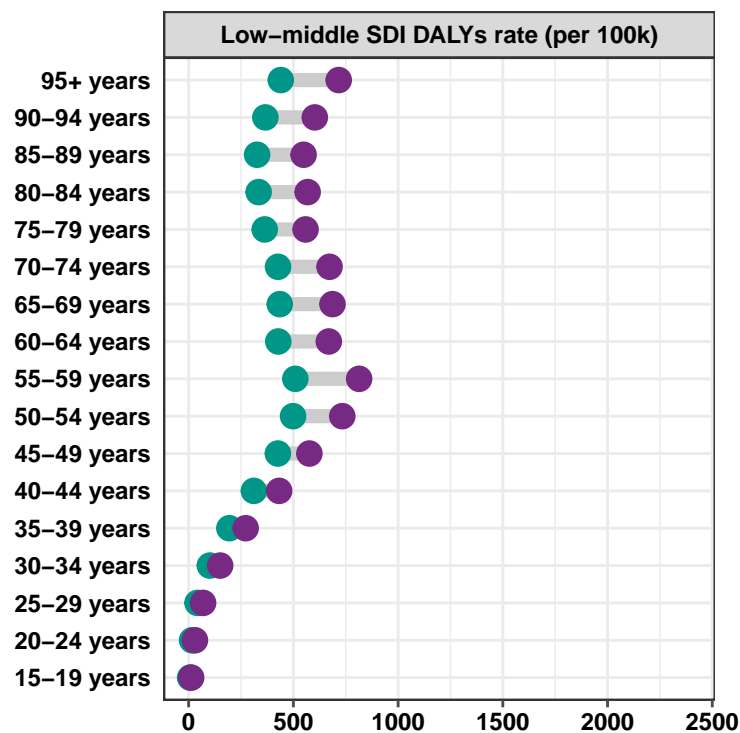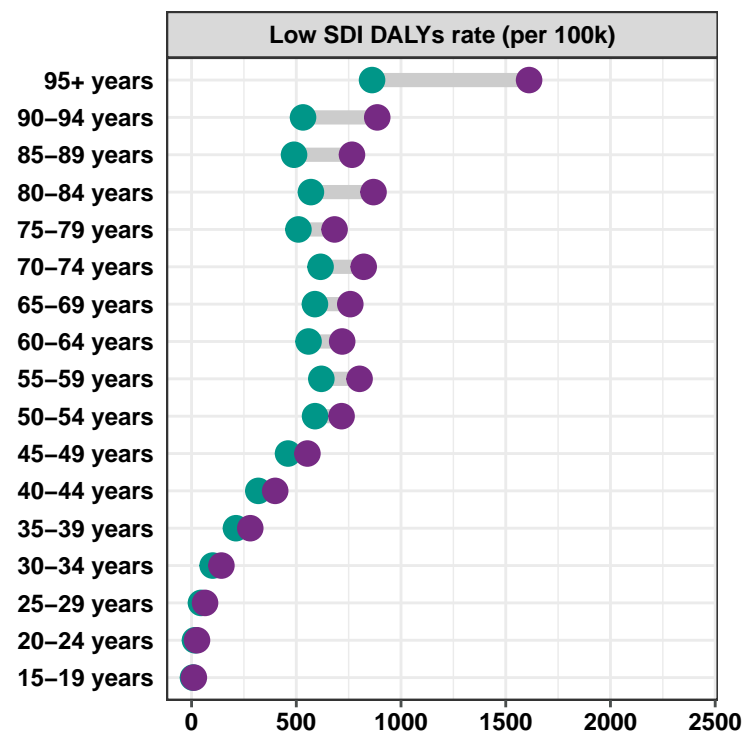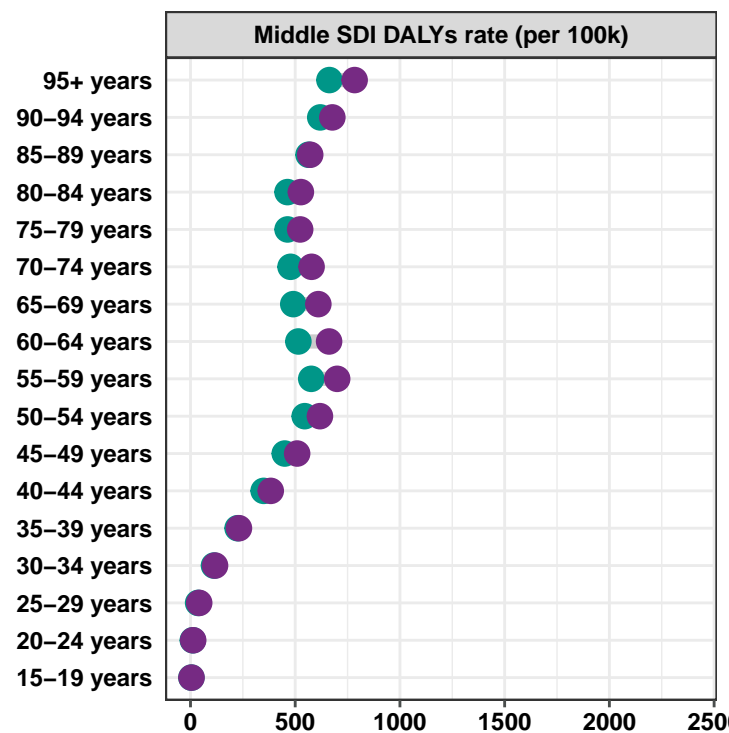

1990 2021

Supplement: Supplementary file 4 — Supplementary Material 4: Fig. S4. Breast cancer DALYs by age group, global and 5 SDI regions. [file 40364_2024_631_MOESM4_ESM.pdf]

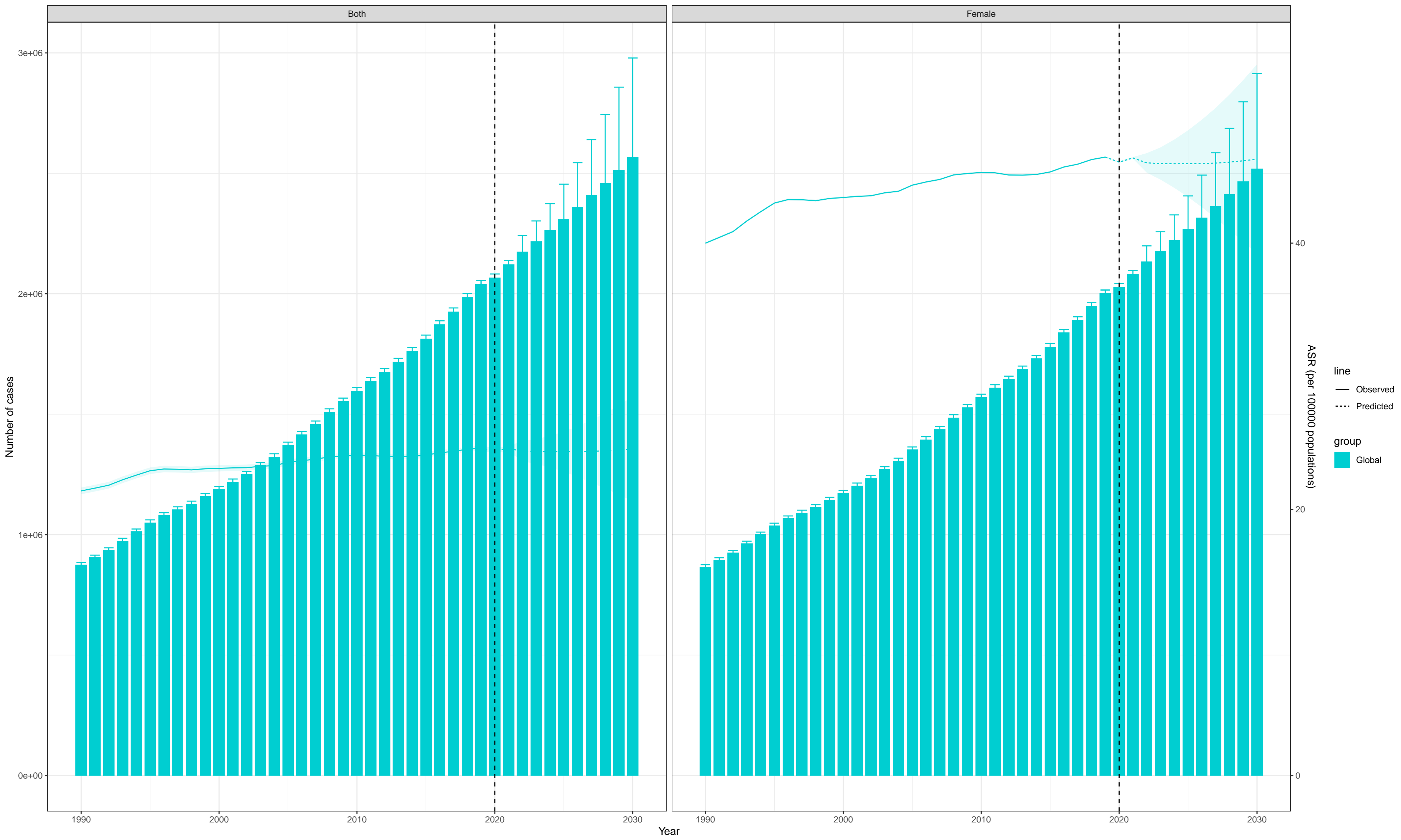

Supplement: Supplementary file 5 — Supplementary Material 5: Fig. S5. Future Forecasts of GBD in Breast cancer incidence. [file 40364_2024_631_MOESM5_ESM.pdf]

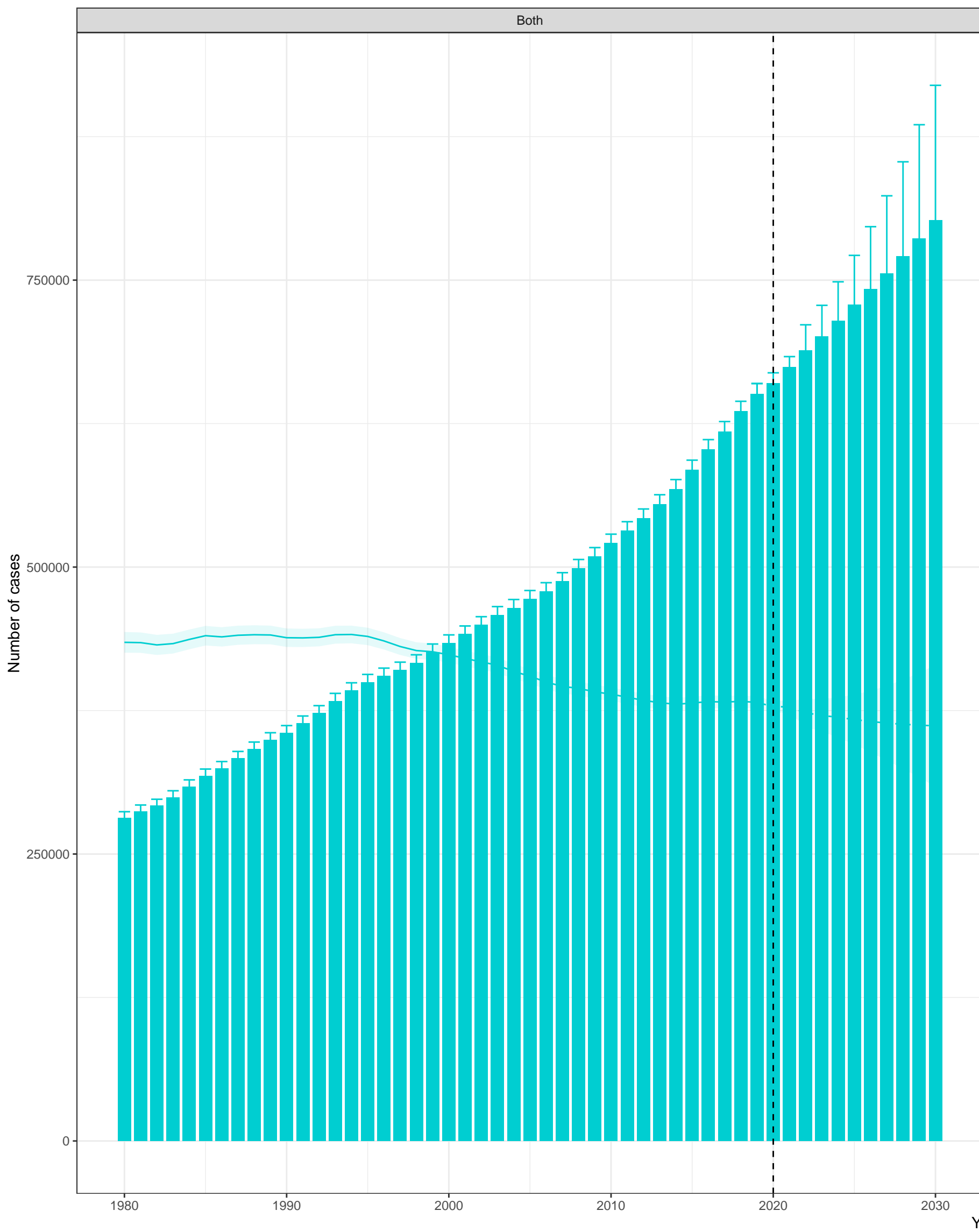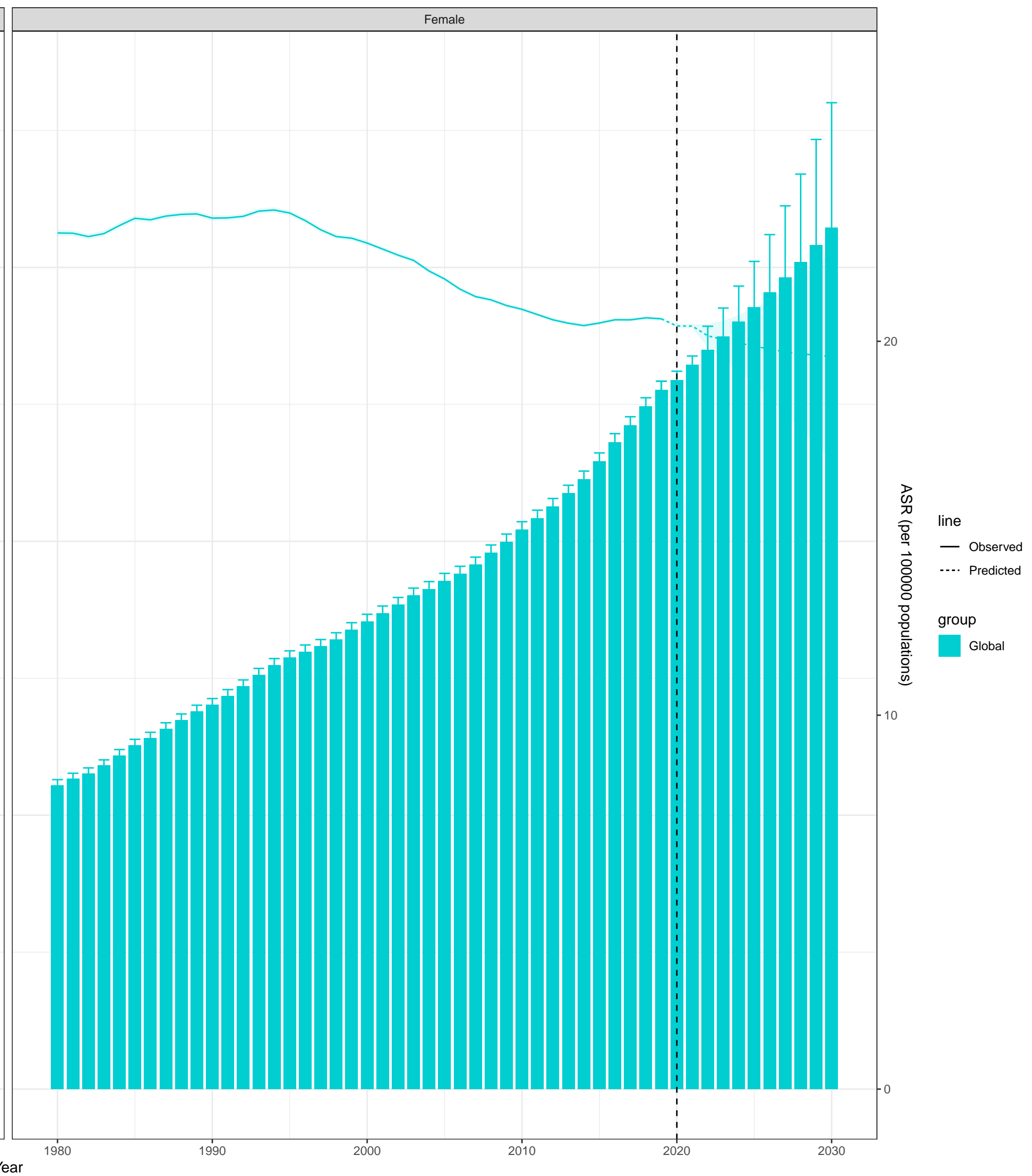

Supplement: Supplementary file 6 — Supplementary Material 6: Fig. S6. Future Forecasts of GBD in Breast cancer deaths. [file 40364_2024_631_MOESM6_ESM.pdf]

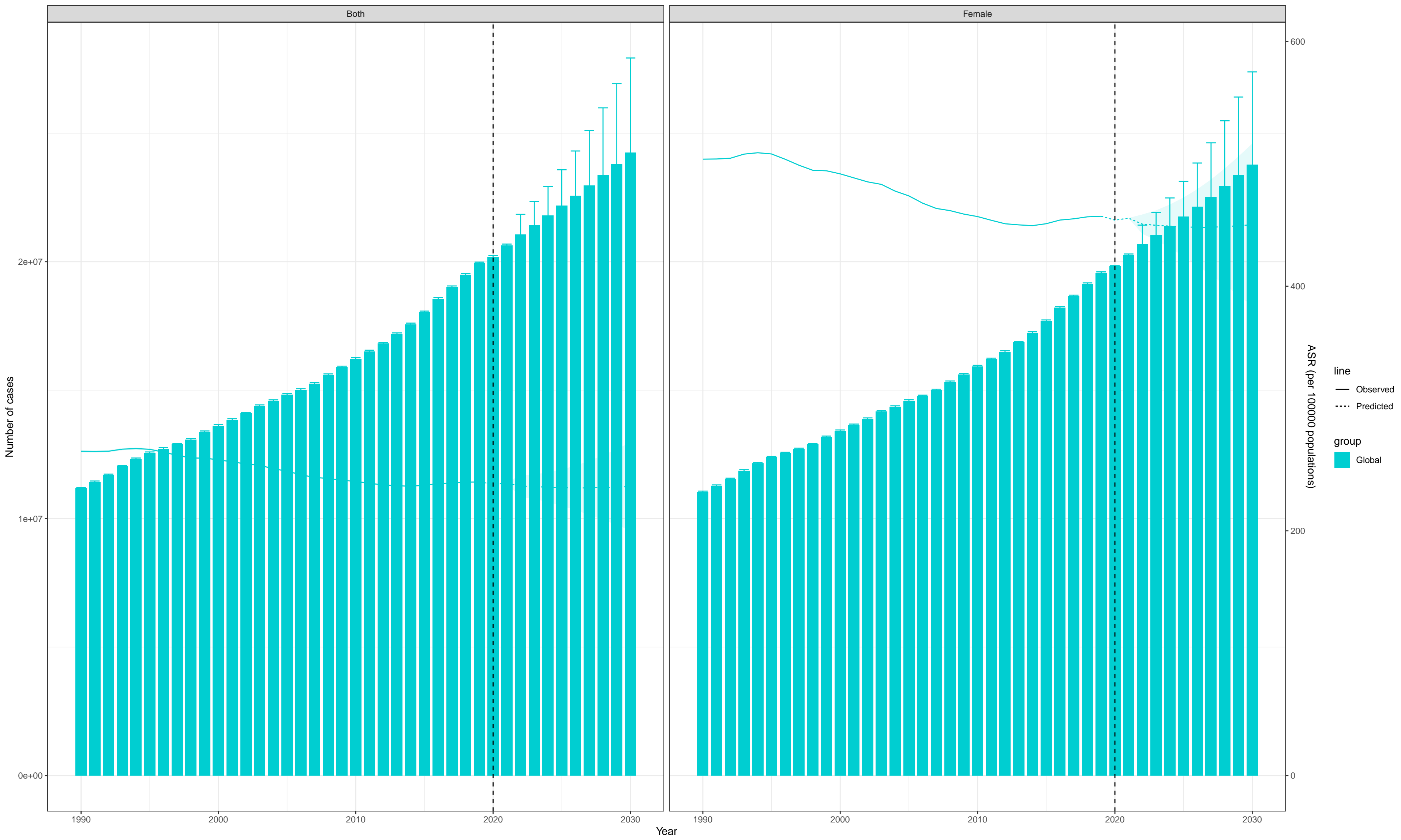

Supplement: Supplementary file 7 — Supplementary Material 7: Fig. S7. Future Forecasts of GBD in Breast cancer DALYs. [file 40364_2024_631_MOESM7_ESM.pdf]
