# Supplementary material for: Global burden of breast cancer and attributable risk factors in 204 countries and territories, from 1990 to 2021: results from the Global Burden of Disease Study 2021
Source: Biomark Res. 2024 Aug 26;12:87. doi: 10.1186/s40364-024-00631-8 (PMC11346191; doi:10.1186/s40364-024-00631-8)
Supplement: Supplementary file 8 — Supplementary Material 8: Table S1. Incidence of Breast Cancer Between 1990 and 2021 at the 204 Countries Level. [file 40364_2024_631_MOESM8_ESM.docx]

| **TableS1 Incidence of Breast Cancer Between 1990 and 2021 at the 204 Countries Level** | | | | | |
| --- | --- | --- | --- | --- | --- |
| **Location** | **1990** | | **2021** | | **EAPC_95%CI** |
|  | **Num**ber(95%UI) | **ASR**(95%UI) | **Num**ber(95%UI) | **ASR**(95%UI) |  |
| Bermuda | 33.7 (30.2-37.4) | 56.8 (50.9-63) | 59.3 (48.4-76.1) | 93.3 (76.1-119.7) | 1.23 (1.06-1.4) |
| Vanuatu | 7.2 (4.9-10.4) | 4.8 (3.2-6.8) | 31 (22.5-40.7) | 9.9 (7.2-13) | 1.99 (1.83-2.15) |
| Sao Tome and Principe | 5.2 (4.2-6.4) | 4.3 (3.5-5.2) | 20.8 (15.4-27.3) | 9.6 (7.1-12.6) | 2.56 (2.37-2.75) |
| Kenya | 846.2 (608.7-1151.5) | 3.7 (2.6-5) | 4823.8 (3386.7-6664.8) | 9.6 (6.8-13.3) | 3.2 (3.02-3.39) |
| Uganda | 884.9 (642.6-1183.2) | 5.1 (3.7-6.8) | 4063.8 (2963.8-5449.6) | 9.4 (6.8-12.6) | 1.4 (1.16-1.64) |
| United Republic of Tanzania | 1454.4 (1168.2-1765.2) | 5.6 (4.5-6.8) | 5378.9 (3935.6-7111.7) | 9.2 (6.7-12.2) | 1.51 (1.35-1.66) |
| Papua New Guinea | 281.4 (192.2-399.3) | 6.9 (4.7-9.7) | 954.9 (691.5-1296.1) | 9.1 (6.6-12.4) | 0.72 (0.49-0.95) |
| France | 32634 (30355.6-34760) | 56.5 (52.5-60.2) | 59205.4 (52391.7-65570.5) | 89.2 (78.9-98.8) | 0 (0-0) |
| Barbados | 106.2 (96.9-115.6) | 41.9 (38.2-45.6) | 259.6 (205.5-326.6) | 86.8 (68.7-109.2) | 2.71 (2.51-2.92) |
| Greece | 6055.9 (5633.7-6436.8) | 58.3 (54.2-62) | 8771.2 (7767.5-9580.2) | 86.2 (76.3-94.2) | 0 (0-0) |
| Italy | 38620.2 (36257.7-40745.2) | 68 (63.8-71.7) | 51530.2 (44988.1-55879.8) | 86.2 (75.2-93.4) | 0.62 (0.43-0.82) |
| Germany | 49694.7 (46450.3-52861.1) | 62.2 (58.1-66.1) | 73076.6 (65325.5-79851.3) | 85.6 (76.5-93.5) | 1.18 (0.96-1.39) |
| United States of America | 204920.2 (194241.6-211264.9) | 80.6 (76.4-83.1) | 272387.5 (251345.1-285257.7) | 81.9 (75.6-85.8) | -0.13 (-0.2--0.06) |
| Mauritania | 103.7 (74.2-141) | 5 (3.6-6.9) | 383 (285.3-507.1) | 8.7 (6.5-11.5) | 1.6 (1.42-1.79) |
| Coted'Ivoire | 518.3 (401.8-676.2) | 4.2 (3.3-5.5) | 2319 (1640.4-3146.2) | 8.3 (5.9-11.3) | 2.3 (2.21-2.38) |
| Togo | 141.4 (112.2-177.8) | 3.9 (3.1-4.9) | 694.7 (487.8-951.1) | 8.3 (5.8-11.4) | 2.45 (2.28-2.62) |
| Bangladesh | 2482.7 (1856.8-3397.5) | 2.3 (1.7-3.1) | 13210.9 (9919.1-16953) | 8 (6-10.3) | 4.21 (3.99-4.43) |
| Netherlands | 9286.1 (8650.6-9857.2) | 62.2 (58-66.1) | 13450.1 (12102.8-14773.3) | 78.2 (70.3-85.8) | 0 (0-0) |
| Finland | 2700.9 (2526.3-2864.2) | 53.9 (50.4-57.2) | 4276.4 (3748.8-4750.8) | 77.2 (67.7-85.8) | 0 (0-0) |
| Belgium | 7681.9 (7086-8203.3) | 77 (71-82.2) | 8666.4 (7605-9577.3) | 75.6 (66.3-83.5) | 0 (0-0) |
| Malta | 193.9 (178.8-211.9) | 52.3 (48.2-57.2) | 331.2 (287.9-377.5) | 74.9 (65.1-85.4) | 0.94 (0.73-1.16) |
| Bulgaria | 3359.9 (2966.6-3834.6) | 38.7 (34.2-44.2) | 5071.1 (4090.8-6087.5) | 74.7 (60.3-89.7) | 2.35 (2.22-2.47) |
| United Kingdom | 44648 (42772.1-45691.7) | 77.9 (74.6-79.7) | 49440.2 (45756-51651.3) | 72.9 (67.4-76.1) | -0.19 (-0.31--0.07) |
| Andorra | 22.6 (15.7-32.5) | 41.6 (28.8-59.8) | 61.1 (41-84.9) | 71.4 (47.9-99.2) | 0 (0-0) |
| Cyprus | 261.8 (216.8-317.7) | 33.6 (27.9-40.8) | 957.1 (781.7-1158.1) | 70.5 (57.6-85.3) | 2.86 (2.51-3.22) |
| Burkina Faso | 626.6 (477-801.1) | 6.6 (5-8.4) | 1794.5 (1260-2409.6) | 7.9 (5.5-10.6) | 0.63 (0.49-0.77) |
| Maldives | 6.7 (3.4-11.6) | 3 (1.5-5.2) | 39.6 (29.9-50.6) | 7.7 (5.8-9.8) | 3.09 (2.69-3.49) |
| Senegal | 279 (218.5-350.1) | 3.7 (2.9-4.6) | 1189.6 (886.7-1604.5) | 7.5 (5.6-10.1) | 2.35 (2.22-2.48) |
| Tajikistan | 369 (300.3-444.3) | 6.9 (5.6-8.3) | 766.2 (467.5-1139.2) | 7.5 (4.6-11.2) | 0.28 (0-0.57) |
| Malawi | 397.8 (310.3-493.8) | 4.1 (3.2-5) | 1421.2 (1014.3-1868.1) | 7.3 (5.2-9.6) | 1.78 (1.54-2.02) |
| Ethiopia | 2619.7 (1711.1-3912.3) | 5.2 (3.4-7.7) | 7869.3 (6358.3-9705.9) | 7.2 (5.8-8.9) | 0.92 (0.57-1.27) |
| Madagascar | 642.1 (507.1-806.8) | 5.4 (4.3-6.8) | 2048.1 (1456.8-2805.5) | 7.2 (5.1-9.8) | 0.82 (0.5-1.15) |
| Timor-Leste | 23 (14.1-34.6) | 2.9 (1.8-4.4) | 99.4 (67.4-135.4) | 7.1 (4.8-9.7) | 3.19 (2.99-3.38) |
| Cameroon | 518 (412-641.3) | 5 (3.9-6.1) | 2235.4 (1582.5-3125.6) | 7 (5-9.8) | 0.98 (0.79-1.18) |
| Guinea-Bissau | 46.1 (31.4-67.3) | 4.6 (3.1-6.7) | 143.9 (101.2-196.9) | 7 (4.9-9.5) | 1.37 (1.28-1.47) |
| Angola | 399.2 (281.1-563.8) | 3.9 (2.7-5.5) | 2291.1 (1563.4-3134.6) | 7 (4.8-9.6) | 2.04 (1.86-2.22) |
| Portugal | 4421.9 (4136.2-4747.5) | 43.6 (40.8-46.8) | 7310.2 (6495.6-7997.3) | 68.9 (61.2-75.4) | 1.5 (1.3-1.7) |
| Canada | 17032.5 (15610.8-18434.1) | 62.5 (57.3-67.6) | 25610.5 (22765.2-28545.2) | 68.4 (60.8-76.2) | 0 (0-0) |
| Montenegro | 226.3 (176.7-296.1) | 36.1 (28.2-47.3) | 418.5 (322.3-533.1) | 67.7 (52.2-86.3) | 0 (0-0) |
| New Zealand | 2145.3 (1981.9-2305.8) | 62.8 (58-67.5) | 3483 (3086.4-3813) | 67.4 (59.7-73.8) | 0 (0-0) |
| Croatia | 2004.5 (1754.2-2270.5) | 41.2 (36.1-46.7) | 2798.6 (2360-3222.5) | 66.5 (56.1-76.6) | 1.67 (1.46-1.89) |
| Serbia | 3272.3 (2471.5-4177.2) | 34 (25.7-43.4) | 5924.7 (4494.8-7582.4) | 66.4 (50.4-85) | 0 (0-0) |
| Denmark | 3746.8 (3531.1-3950.3) | 72.8 (68.6-76.8) | 3861 (3447-4269.7) | 66 (58.9-73) | 0 (0-0) |
| Hungary | 4458.2 (3967.4-5011.5) | 42.9 (38.2-48.2) | 6160.6 (5209.9-7202.1) | 64.2 (54.3-75) | 0 (0-0) |
| Puerto Rico | 1016.1 (944.2-1092.1) | 28.1 (26.1-30.2) | 2106.6 (1742.1-2512.3) | 63.9 (52.9-76.3) | 2.7 (2.55-2.85) |
| Australia | 7756 (7335.2-8205.5) | 46 (43.5-48.7) | 16366.5 (14402.8-18461.4) | 63.5 (55.8-71.6) | 0 (0-0) |
| Ireland | 1860.7 (1748.3-1989.8) | 51.7 (48.5-55.2) | 3126.4 (2787.2-3456.3) | 63.3 (56.4-69.9) | 0.93 (0.79-1.07) |
| Cook Islands | 4.7 (3.6-6) | 24.7 (18.8-31.9) | 11.1 (8.3-14.4) | 62.4 (46.8-81.3) | 3.14 (2.95-3.32) |
| Iceland | 127.2 (114.6-139.1) | 50.1 (45.1-54.8) | 216 (189.4-242) | 61.7 (54.1-69.1) | 0 (0-0) |
| Japan | 28197.8 (26549.3-29664.6) | 22.4 (21.1-23.6) | 78401 (68142.2-85214.5) | 61.4 (53.4-66.7) | 0 (0-0) |
| Nepal | 556.4 (407.3-729) | 2.9 (2.1-3.7) | 2141.3 (1543.7-2938.5) | 6.9 (5-9.4) | 3.06 (2.72-3.41) |
| Democratic Republic of the Congo | 1680.7 (1166.3-2311.6) | 4.4 (3.1-6.1) | 6205.9 (4588.2-8484.6) | 6.9 (5.1-9.4) | 1.4 (1.05-1.76) |
| Bhutan | 14.8 (10.2-19.7) | 2.4 (1.6-3.1) | 52.1 (36.2-72.8) | 6.9 (4.8-9.6) | 3.29 (3.14-3.45) |
| Mozambique | 636.8 (525.2-783.7) | 4.8 (3.9-5.9) | 2143.3 (1488.1-2848.3) | 6.9 (4.8-9.2) | 1.38 (1.22-1.54) |
| Sudan | 522.6 (320.2-813.4) | 2.6 (1.6-4.1) | 2853.4 (1677.9-4511.1) | 6.6 (3.9-10.4) | 3.07 (2.79-3.35) |
| Afghanistan | 523 (265.6-902.5) | 5.3 (2.7-9.1) | 2043.4 (1006-3724.7) | 6.5 (3.2-11.9) | 1.03 (0.48-1.59) |
| Liberia | 86.7 (67.9-108.7) | 3.5 (2.8-4.4) | 342.7 (233.5-496.9) | 6.3 (4.3-9.1) | 1.28 (0.95-1.6) |
| Central African Republic | 138.3 (97.9-189) | 5.1 (3.6-6.9) | 346.7 (232.4-490.1) | 6.3 (4.2-8.9) | 0.69 (0.49-0.88) |
| South Sudan | 237.6 (167.3-333.2) | 4 (2.8-5.7) | 578.8 (406.1-837.5) | 6 (4.2-8.7) | 1.13 (0.63-1.63) |
| Guinea | 284.7 (214.1-358.6) | 4.8 (3.6-6) | 799.8 (563.3-1121.1) | 6 (4.2-8.3) | 0.62 (0.54-0.69) |
| Uruguay | 1353.8 (1266.6-1434.1) | 43.1 (40.3-45.7) | 2036.1 (1829.8-2213.6) | 59.8 (53.7-65) | 0 (0-0) |
| Slovenia | 780.8 (709.4-856.8) | 39.6 (35.9-43.4) | 1233.6 (1011.6-1478.7) | 59.6 (48.9-71.4) | 0 (0-0) |
| Bahamas | 73.4 (66.8-80.4) | 28.6 (26-31.3) | 230.8 (185.9-285.2) | 59.5 (47.9-73.5) | 2.68 (2.5-2.87) |
| Switzerland | 3819.9 (3554.3-4071.7) | 55.6 (51.8-59.3) | 5294.9 (4682.7-5853.2) | 59.3 (52.5-65.6) | 0 (0-0) |
| Austria | 4607.5 (4256.4-4942.1) | 59.3 (54.8-63.6) | 5296.5 (4717.9-5834.6) | 59 (52.5-65) | 0.15 (0-0.29) |
| Spain | 17607.4 (16339.7-18751.9) | 45.4 (42.1-48.4) | 26779.7 (23479.7-29682.1) | 58.8 (51.5-65.2) | 0 (0-0) |
| Luxembourg | 244.1 (227.6-260.2) | 64 (59.7-68.3) | 377.5 (333.1-419.1) | 58.6 (51.7-65) | 0.17 (-0.07-0.41) |
| Sweden | 5150.6 (4779.8-5543.5) | 60 (55.7-64.6) | 5996.8 (4991.6-6924) | 57.8 (48.1-66.7) | 0.24 (-0.13-0.62) |
| Antigua and Barbuda | 16.4 (14.7-18.2) | 27.3 (24.4-30.2) | 51.4 (47.6-56) | 57.5 (53.3-62.6) | 2.76 (2.48-3.04) |
| Latvia | 930.8 (826.8-1057.8) | 35 (31.1-39.8) | 1074.7 (878.6-1268.1) | 57.5 (47-67.8) | 1.63 (1.45-1.82) |
| United States Virgin Islands | 34.8 (28.6-42) | 32.8 (26.9-39.6) | 48.5 (33.4-68.4) | 56.5 (38.9-79.6) | 1.96 (1.78-2.13) |
| Lebanon | 555.9 (390-757.3) | 18.6 (13-25.3) | 3123.4 (2480.8-3848.6) | 56.4 (44.8-69.5) | 3.97 (3.53-4.41) |
| Estonia | 585.6 (533.9-641.6) | 37.3 (34-40.9) | 734.9 (597.2-870.9) | 56.1 (45.6-66.4) | 0 (0-0) |
| Czechia | 4226.6 (3789.4-4682.3) | 41.1 (36.8-45.5) | 5949.5 (4945-7034.9) | 56 (46.5-66.2) | 0.63 (0.32-0.94) |
| Poland | 9632 (9203-10059.3) | 25.2 (24.1-26.4) | 21058.7 (18540.7-23510.4) | 55.1 (48.5-61.5) | 0 (0-0) |
| Slovakia | 1472.5 (1279.4-1712.8) | 27.9 (24.2-32.4) | 2990 (2282-3710.3) | 55.1 (42-68.3) | 2.25 (2.11-2.38) |
| Lithuania | 1186 (1090.1-1289.1) | 32.3 (29.7-35.1) | 1486.7 (1238.9-1756.5) | 54.5 (45.4-64.4) | 0 (0-0) |
| Georgia | 2033.6 (1813.8-2244.4) | 36.8 (32.8-40.6) | 1963.4 (1683.7-2273.8) | 54.4 (46.7-63) | 1.48 (1.2-1.76) |
| Russian Federation | 39422.7 (38456-40330.3) | 26.1 (25.5-26.7) | 76078.6 (67845.3-83982.1) | 52.5 (46.8-58) | 2 (1.85-2.15) |
| Cuba | 2699.6 (2507.9-2887) | 24.9 (23.1-26.6) | 5885.3 (4988.9-7002.9) | 52.2 (44.3-62.1) | 2.5 (2.38-2.63) |
| Romania | 5009.3 (4650-5417.5) | 21.4 (19.9-23.2) | 9728.1 (8488.2-11065.2) | 51.4 (44.8-58.4) | 2.87 (2.74-3.01) |
| Côte d'Ivoire | 490.1 (403.2-581.2) | 24.6 (20.2-29.2) | 1119.1 (857-1446.7) | 51.4 (39.4-66.5) | 0 (0-0) |
| Costa Rica | 435.1 (399.9-474.3) | 14.3 (13.1-15.6) | 2402.1 (2081.6-2745.3) | 50.6 (43.8-57.8) | 4.29 (4.12-4.46) |
| Sierra Leone | 143.7 (101.4-191.8) | 3.5 (2.4-4.6) | 504.9 (353.3-667.6) | 5.7 (4-7.5) | 1.55 (1.44-1.66) |
| Mongolia | 48.9 (38.4-61.9) | 2.3 (1.8-2.9) | 181.9 (141.8-225.2) | 5.5 (4.3-6.7) | 3.15 (3.01-3.29) |
| Mali | 429.5 (349.5-518.7) | 5 (4-6) | 1310.4 (935.4-1824.5) | 5.4 (3.9-7.6) | 0.19 (0.09-0.29) |
| Oman | 35.4 (25.2-49.1) | 1.8 (1.3-2.5) | 243.8 (181.5-319.6) | 5.2 (3.9-6.8) | 3.56 (3.11-4.01) |
| Yemen | 241 (147.6-345.4) | 1.8 (1.1-2.5) | 1722.2 (1169.7-2437.8) | 5.1 (3.5-7.2) | 3.7 (3.53-3.88) |
| San Marino | 11.5 (9-14.6) | 48.3 (38.1-61.7) | 16.3 (9.6-24) | 49.9 (29.2-73.2) | 0.84 (0.51-1.17) |
| Norway | 2045.7 (1922.8-2147.4) | 48.2 (45.3-50.6) | 2699.5 (2448.1-2938.3) | 49.8 (45.2-54.2) | 0.02 (-0.29-0.33) |
| Belarus | 2942 (2662.7-3265.5) | 28.2 (25.5-31.3) | 4445.6 (3472.8-5569.8) | 47.7 (37.2-59.7) | 0 (0-0) |
| Trinidad and Tobago | 240 (221-261.6) | 19.9 (18.3-21.7) | 663.7 (498.2-852.3) | 47.6 (35.8-61.2) | 3.01 (2.84-3.18) |
| Taiwan (Province of China) | 2136.2 (1998.9-2284.3) | 10.5 (9.8-11.2) | 10867.3 (9842.4-11888.9) | 46 (41.6-50.3) | 5 (4.57-5.44) |
| Venezuela (Bolivarian Republic of) | 2164.4 (2027.5-2307.7) | 11.5 (10.8-12.3) | 12010.3 (9029.8-15577.2) | 45.1 (33.9-58.5) | 4.19 (4-4.39) |
| Saint Kitts and Nevis | 13.4 (12.2-14.6) | 32.2 (29.4-35.3) | 26.4 (21.6-32) | 45 (36.8-54.6) | 1.62 (1.34-1.89) |
| Bosnia and Herzegovina | 680.9 (583.6-776) | 15.1 (13-17.3) | 1468.7 (1143.2-1825.3) | 44.5 (34.6-55.3) | 4.03 (3.7-4.37) |
| Jamaica | 407.2 (373.1-439.6) | 17.2 (15.8-18.6) | 1243.6 (931.5-1623.2) | 44.4 (33.3-58) | 0 (0-0) |
| Republic of Moldova | 1145.7 (1013.5-1282.3) | 25.8 (22.8-28.8) | 1586.3 (1323.3-1893.1) | 44.1 (36.8-52.7) | 2.06 (1.81-2.3) |
| Grenada | 18.3 (16.5-20.3) | 21.1 (19-23.4) | 45.1 (38.8-51.7) | 43.9 (37.8-50.3) | 2.7 (2.38-3.02) |
| Israel | 1890.7 (1750.3-2030.1) | 38.1 (35.3-40.9) | 4171.6 (3675.5-4644.9) | 43.5 (38.3-48.4) | 0 (0-0) |
| Palau | 3.8 (2.9-5) | 24.9 (18.8-32.9) | 7.8 (5.9-10.1) | 43 (32.7-55.6) | 1.55 (1.41-1.7) |
| Saint Vincent and the Grenadines | 21.8 (19.5-23.9) | 19.9 (17.8-21.9) | 49 (42.4-56.9) | 42.9 (37.2-49.8) | 2.58 (2.35-2.82) |
| Singapore | 581.3 (539.4-628.1) | 19.1 (17.7-20.6) | 2452.4 (2243.2-2666.3) | 42.8 (39.2-46.6) | 0 (0-0) |
| Dominica | 17.1 (14.5-19.8) | 23.6 (20.1-27.3) | 28.2 (21.5-35.7) | 42 (32-53.2) | 1.93 (1.86-2) |
| Saint Lucia | 27.2 (25.2-29.4) | 19.9 (18.4-21.5) | 73.6 (60.9-88.8) | 41.5 (34.3-50) | 0 (0-0) |
| Burundi | 302.7 (213.8-427.9) | 5.5 (3.8-7.7) | 650.2 (475.2-877.2) | 4.9 (3.6-6.6) | -0.8 (-1.07--0.53) |
| Benin | 166.3 (133.1-199.1) | 3.4 (2.7-4.1) | 644.9 (459-868.7) | 4.8 (3.4-6.4) | 0.98 (0.85-1.1) |
| Mauritius | 89.2 (82.5-95.9) | 8.1 (7.5-8.8) | 503.9 (455.3-542.4) | 39.6 (35.8-42.6) | 4.51 (4.12-4.9) |
| Niue | 0.5 (0.4-0.7) | 22.2 (16.9-28.9) | 0.7 (0.5-0.9) | 39.5 (29.7-51.3) | 1.59 (1.48-1.7) |
| Armenia | 943.5 (881.1-1012) | 27.6 (25.8-29.6) | 1170.4 (1004.2-1344.4) | 39.1 (33.5-44.9) | 1.16 (0.8-1.52) |
| Colombia | 3829.7 (3545.6-4111.5) | 11.8 (10.9-12.7) | 19094.8 (15798.5-22988.1) | 38.9 (32.2-46.9) | 4.02 (3.84-4.19) |
| Panama | 292 (267.1-316.5) | 12.2 (11.2-13.2) | 1627.7 (1307.2-1994) | 37.9 (30.5-46.5) | 3.81 (3.68-3.95) |
| Thailand | 4594 (3714.6-5476.9) | 8.1 (6.5-9.6) | 24635.6 (18506.3-31808.3) | 36.9 (27.8-47.7) | 5.15 (4.85-5.46) |
| Ukraine | 19088.5 (17613.5-20548.6) | 36.2 (33.4-39) | 15687.5 (10102.2-22758.7) | 36.4 (23.5-52.8) | -0.47 (-0.65--0.29) |
| Argentina | 9039.8 (8463.5-9605.9) | 27.3 (25.6-29) | 15948.8 (14609.4-17342.2) | 35.1 (32.1-38.1) | 0.78 (0.61-0.96) |
| American Samoa | 5.9 (4.8-7.2) | 12.2 (10-14.9) | 17.5 (13.7-21.8) | 35.1 (27.5-43.8) | 3.58 (3.45-3.7) |
| Bahrain | 61.1 (51.8-71.6) | 12.1 (10.2-14.1) | 531 (412.7-681.9) | 34.7 (27-44.6) | 3.09 (2.83-3.35) |
| Turkey | 2440.8 (1960-2983.9) | 4.2 (3.4-5.2) | 28899.7 (22612-35853.3) | 34.6 (27-42.9) | 8.63 (7.73-9.53) |
| Northern Mariana Islands | 6.4 (4.5-8.7) | 14.1 (9.9-19.3) | 15.5 (12.5-18.3) | 31.9 (25.8-37.6) | 2.66 (2.5-2.82) |
| Republic of Korea | 2589.7 (2244.8-3057.8) | 5.9 (5.1-6.9) | 16186.4 (13078.8-19147.5) | 31.4 (25.4-37.1) | 5.96 (5.52-6.4) |
| Tokelau | 0.3 (0.2-0.4) | 16.9 (11-23.9) | 0.4 (0.3-0.6) | 30.7 (22.6-41.2) | 1.88 (1.77-1.98) |
| Gambia | 16.6 (12.2-22.3) | 1.7 (1.2-2.3) | 92.7 (66.6-125) | 3.9 (2.8-5.2) | 2.49 (2.26-2.72) |
| Somalia | 284.9 (193.7-395.3) | 3.6 (2.4-5) | 803.6 (527.4-1122.7) | 3.7 (2.4-5.2) | -0.21 (-0.33--0.09) |
| Chad | 184.1 (131.7-241) | 3.1 (2.2-4) | 584.6 (410-780.1) | 3.3 (2.3-4.4) | 0.29 (0.21-0.38) |
| Malaysia | 1859.5 (1571.4-2172.6) | 10.5 (8.9-12.3) | 9434.9 (7905.3-11197.9) | 29.7 (24.8-35.2) | 3.49 (3.37-3.62) |
| Tunisia | 636.5 (520.6-780.3) | 7.6 (6.2-9.3) | 3487.1 (2483.7-4860.3) | 29.4 (21-41) | 4.41 (4.27-4.55) |
| Iran (Islamic Republic of) | 3201 (2681.2-3803.5) | 5.6 (4.7-6.7) | 24764.1 (22197.5-27715.3) | 29 (26-32.5) | 5.9 (5.58-6.23) |
| Mexico | 7427.9 (7215.7-7634.9) | 8.7 (8.5-8.9) | 36561.7 (30342.9-43119.3) | 28.3 (23.5-33.4) | 0 (0-0) |
| China | 86708.7 (70225.3-105273.3) | 7.4 (6-8.9) | 402794.2 (312117.3-505644.3) | 28.3 (21.9-35.5) | 4.6 (4.49-4.72) |
| Seychelles | 7.8 (6.7-9) | 10.7 (9.2-12.4) | 29.5 (25-34.6) | 28 (23.7-32.8) | 3.24 (2.9-3.59) |
| Brazil | 16011.1 (15388.6-16617) | 10.8 (10.4-11.2) | 61092.7 (57179.7-64461.1) | 27.7 (25.9-29.3) | 2.85 (2.76-2.94) |
| Fiji | 120.1 (94.4-152.7) | 15.8 (12.4-20.1) | 250.5 (181.7-328.7) | 27.1 (19.7-35.6) | 1.78 (1.63-1.94) |
| Greenland | 11.2 (8.8-14.2) | 20.1 (15.9-25.5) | 15.2 (11.1-20) | 27 (19.7-35.6) | 0.94 (0.84-1.03) |
| Chile | 1758.5 (1641.2-1882) | 13.2 (12.4-14.2) | 4987.2 (4480.4-5514.2) | 26.5 (23.8-29.3) | 2.66 (2.52-2.8) |
| Tonga | 17.2 (13.6-21.9) | 17.4 (13.7-22.2) | 28.2 (20.6-38) | 26.5 (19.3-35.7) | 1.06 (0.94-1.17) |
| Kuwait | 143.7 (128.5-160.4) | 8.4 (7.5-9.3) | 1204.4 (1019.7-1428.7) | 25.9 (21.9-30.7) | 2.66 (2.1-3.22) |
| El Salvador | 308.6 (265.8-354.1) | 5.8 (5-6.7) | 1636.7 (1276.2-2072.4) | 25.4 (19.8-32.1) | 4.98 (4.7-5.26) |
| Brunei Darussalam | 23.2 (17-30.5) | 9 (6.6-11.8) | 111.5 (86.2-138.3) | 24.7 (19.1-30.7) | 0 (0-0) |
| Jordan | 296.5 (224-392.5) | 7.9 (6-10.5) | 3030.9 (2143.9-4111.8) | 24.6 (17.4-33.4) | 3.95 (3.61-4.3) |
| Guyana | 74.4 (64.5-85.2) | 9.5 (8.3-10.9) | 184.9 (137.6-243.7) | 24.2 (18-31.9) | 3.57 (3.28-3.86) |
| Syrian Arab Republic | 627.3 (464.6-797.9) | 4.9 (3.7-6.3) | 3371.2 (2459.8-4534.6) | 24 (17.5-32.3) | 5.16 (4.76-5.55) |
| Palestine | 209.1 (146.8-290.3) | 10.2 (7.2-14.2) | 1202.3 (971.9-1470.7) | 23.4 (18.9-28.6) | 2.87 (2.69-3.05) |
| Albania | 200.6 (157.6-255.4) | 6.1 (4.8-7.7) | 620 (438.4-829.9) | 23.2 (16.4-31.1) | 5.15 (4.73-5.58) |
| Iraq | 1256.6 (929.4-1649.6) | 6.8 (5-9) | 9554.2 (6666.1-12992.4) | 23.2 (16.2-31.5) | 4.16 (3.99-4.32) |
| Libya | 211.2 (165.5-273.2) | 5 (3.9-6.5) | 1563.4 (1121-2190.1) | 22.8 (16.3-31.9) | 5.28 (5.06-5.5) |
| Suriname | 42.8 (35.7-50.2) | 11.1 (9.2-13) | 130.5 (99.9-166.8) | 22.5 (17.2-28.8) | 2.55 (2.41-2.7) |
| South Africa | 3454.4 (2814.4-4100.4) | 9.3 (7.6-11.1) | 12094.2 (10835.3-13506.2) | 21.3 (19.1-23.8) | 3.22 (2.97-3.46) |
| Micronesia (Federated States of) | 9.8 (6.6-13.5) | 9.4 (6.4-13) | 21.4 (15.2-28.9) | 20.9 (14.8-28.2) | 2.62 (2.55-2.7) |
| Qatar | 42.5 (33.2-52.5) | 9.6 (7.5-11.8) | 603.8 (432.3-833.6) | 20.3 (14.5-28) | 1.81 (1.48-2.14) |
| Nauru | 1.3 (0.7-2) | 12.3 (7.3-19.1) | 2.2 (1.3-3.5) | 20.2 (11.7-31.4) | 1.42 (1.32-1.53) |
| Sri Lanka | 1105.9 (921-1325.6) | 6.5 (5.4-7.7) | 4474.8 (2874-6152.3) | 20.1 (12.9-27.6) | 4.1 (3.9-4.31) |
| Guam | 14.9 (12.8-17.5) | 10.9 (9.3-12.8) | 31.8 (26.5-38.5) | 20 (16.6-24.2) | 2.35 (2.09-2.62) |
| Niger | 175 (129.6-234.8) | 2.2 (1.6-2.9) | 661.3 (449.4-923.3) | 2.6 (1.8-3.7) | 0.6 (0.55-0.66) |
| Azerbaijan | 907.6 (754-1040) | 12.4 (10.3-14.2) | 2063.9 (1506.8-2680.3) | 19.7 (14.3-25.5) | 1.75 (1.49-2) |
| Egypt | 3006.2 (2572.3-3564.9) | 5.4 (4.6-6.4) | 20680 (16203.6-25725.2) | 19.6 (15.3-24.4) | 0 (0-0) |
| Paraguay | 296.9 (239.2-359.8) | 7.3 (5.9-8.9) | 1402.7 (1028.3-1854.3) | 19.6 (14.3-25.9) | 3.16 (3.01-3.3) |
| Tuvalu | 1.2 (0.8-1.8) | 13 (8.5-19) | 2.4 (1.7-3.4) | 19.6 (13.9-27.2) | 1.04 (0.86-1.22) |
| Namibia | 96.2 (79.4-117.3) | 6.9 (5.7-8.4) | 477.6 (308.2-684.5) | 19.6 (12.7-28.2) | 3.81 (3.67-3.94) |
| Philippines | 5321.8 (4744.8-5933.6) | 8.4 (7.5-9.4) | 21561.5 (16967.2-26892.2) | 19 (15-23.7) | 2.62 (2.51-2.73) |
| Kazakhstan | 3077.9 (2729.3-3443.5) | 18.8 (16.6-21) | 3539.7 (2966.5-4149.3) | 18.7 (15.6-21.9) | 0.63 (0.42-0.85) |
| Indonesia | 12228.9 (8475.3-17319.2) | 6.6 (4.6-9.4) | 49532.2 (33319.2-70939.5) | 17.8 (11.9-25.4) | 3.1 (3.02-3.17) |
| Peru | 1467.4 (1195.2-1750.3) | 6.8 (5.5-8.1) | 6360.7 (4542.1-8382.1) | 17.5 (12.5-23.1) | 0 (0-0) |
| Democratic People's Republic of Korea | 1718.3 (1130.5-2515.7) | 8.3 (5.5-12.2) | 4536.4 (3073.8-6257.9) | 17.2 (11.6-23.7) | 2.67 (2.53-2.82) |
| Marshall Islands | 2.9 (2.1-4.1) | 6.4 (4.5-9.1) | 9.6 (5.6-15.1) | 17.1 (10-26.8) | 3.11 (3.08-3.15) |
| Myanmar | 3435.4 (2308.6-4943.4) | 8.5 (5.7-12.2) | 9610.1 (7313.6-12682.9) | 17 (13-22.5) | 2.13 (2.05-2.22) |
| Monaco | 34 (24.8-44.7) | 111.8 (81.4-146.9) | 63 (47.8-83) | 166.5 (126.3-219.3) | 1.43 (1.27-1.59) |
| Saudi Arabia | 491.5 (353-673.7) | 3.1 (2.2-4.2) | 6190.1 (4373.2-8826.2) | 16.4 (11.6-23.4) | 5.54 (5.36-5.71) |
| Viet Nam | 3221.1 (2476.8-4203.2) | 4.7 (3.6-6.2) | 15969.8 (12014.7-21410.1) | 15.9 (12-21.4) | 4.23 (4.16-4.29) |
| Kiribati | 6.9 (5.4-8.8) | 9.3 (7.2-11.8) | 19.2 (14.4-26) | 15.9 (11.9-21.5) | 1.76 (1.71-1.81) |
| Cambodia | 520.5 (328.7-796.7) | 5.1 (3.2-7.8) | 2673 (1917.3-3591.6) | 15.7 (11.2-21.1) | 3.93 (3.86-3.99) |
| Dominican Republic | 448.8 (378.8-527.2) | 6.3 (5.3-7.4) | 1714.9 (1292.8-2222.1) | 15.6 (11.7-20.2) | 3.17 (2.89-3.46) |
| Gabon | 88.9 (63.4-118.6) | 9 (6.5-12.1) | 282.6 (190.9-392.9) | 15.6 (10.5-21.6) | 1.58 (1.42-1.74) |
| United Arab Emirates | 113.3 (81.4-152.2) | 6.1 (4.4-8.1) | 1475.8 (1059.3-1978) | 15.3 (11-20.5) | 2.62 (2.15-3.09) |
| Ecuador | 468.8 (434.9-500.1) | 4.7 (4.4-5) | 2765.8 (2084.9-3498.6) | 15.3 (11.5-19.4) | 4.01 (3.72-4.29) |
| Morocco | 921.4 (707.9-1210.3) | 3.6 (2.8-4.8) | 5619.5 (3790.7-8227.5) | 15.1 (10.2-22.1) | 4.91 (4.8-5.01) |
| Bolivia (Plurinational State of) | 422.9 (274.6-612.3) | 6.6 (4.3-9.6) | 1772.6 (1162.2-2585.5) | 15 (9.9-21.9) | 2.66 (2.62-2.7) |
| Congo | 184.4 (108.3-294.3) | 7.7 (4.5-12.3) | 807.4 (474.3-1290.8) | 15 (8.8-23.9) | 2.11 (1.86-2.36) |
| Pakistan | 7881.7 (6230-9769.9) | 7.1 (5.6-8.8) | 34748.8 (24741.6-46366.9) | 14.8 (10.5-19.7) | 1.98 (1.84-2.12) |
| Nicaragua | 148.1 (125.3-174.2) | 3.8 (3.2-4.5) | 955.1 (747.3-1213.1) | 14.3 (11.2-18.2) | 4.7 (4.51-4.89) |
| Haiti | 566.7 (339.4-889) | 8.9 (5.3-13.9) | 1803.3 (1099.1-2835.4) | 14 (8.5-22) | 1.59 (1.54-1.65) |
| Comoros | 26.8 (19.3-36.4) | 5.8 (4.2-7.9) | 103.7 (77.5-138) | 13.9 (10.4-18.5) | 2.69 (2.55-2.83) |
| Algeria | 1029.7 (783.5-1343.1) | 4.1 (3.1-5.3) | 6065.4 (4460.1-8053.7) | 13.7 (10.1-18.2) | 0 (0-0) |
| Turkmenistan | 277.1 (241.7-315.6) | 7.5 (6.5-8.5) | 694.9 (509.2-939.5) | 13.5 (9.9-18.2) | 2.63 (2.13-3.13) |
| Belize | 9.4 (8.5-10.2) | 5 (4.5-5.5) | 57.2 (49.8-65.3) | 13.3 (11.6-15.2) | 0 (0-0) |
| Cabo Verde | 26.6 (21.4-32.7) | 7.5 (6.1-9.3) | 74.1 (56.6-96.4) | 13.3 (10.1-17.2) | 2.29 (1.84-2.74) |
| Botswana | 77.2 (52.7-109) | 5.9 (4-8.3) | 313.2 (215.3-451.2) | 13.1 (9-18.9) | 3.14 (2.72-3.55) |
| Samoa | 12.3 (9.3-16) | 7.3 (5.5-9.5) | 28 (20.2-37.3) | 13.1 (9.5-17.5) | 1.81 (1.72-1.89) |
| Zimbabwe | 527.3 (404.3-674.5) | 5.1 (3.9-6.5) | 2004.1 (1439.5-2761.8) | 12.9 (9.2-17.7) | 3.64 (2.94-4.35) |
| Lesotho | 88.9 (62.3-124.8) | 5.8 (4.1-8.1) | 242.6 (154.6-357.2) | 12.9 (8.2-19.1) | 3.2 (2.78-3.62) |
| Eswatini | 41 (31.4-52.5) | 5.1 (3.9-6.5) | 146.4 (81.9-231.1) | 12.7 (7.1-20) | 3.19 (2.84-3.54) |
| Honduras | 193.2 (142.4-248.8) | 4.1 (3-5.3) | 1269 (893.8-1745.8) | 12.6 (8.8-17.3) | 3.64 (3.51-3.77) |
| Uzbekistan | 1637.1 (1469.4-1813.6) | 7.8 (7-8.7) | 4243.6 (3460.2-5085.5) | 12.4 (10.1-14.9) | 1.81 (1.55-2.07) |
| Zambia | 391.9 (281.4-539.4) | 4.9 (3.5-6.8) | 2390.5 (1331.8-3771.4) | 12.2 (6.8-19.3) | 3.09 (2.53-3.64) |
| Kyrgyzstan | 539.3 (480.7-601) | 12.1 (10.8-13.5) | 811.4 (660.6-980.9) | 11.8 (9.6-14.3) | -0.15 (-0.45-0.16) |
| India | 33632.6 (28975.8-39129.9) | 3.9 (3.4-4.6) | 159271.5 (135111.6-187901.5) | 11.3 (9.6-13.3) | 0 (0-0) |
| Nigeria | 5525.7 (4099.7-7247.9) | 6.1 (4.6-8) | 26098.7 (17281.8-37952) | 11.3 (7.5-16.4) | 2.16 (1.83-2.49) |
| Lao People's Democratic Republic | 214 (124.7-350.1) | 5.1 (3-8.4) | 823.6 (578.6-1129.8) | 11.2 (7.8-15.3) | 2.75 (2.6-2.9) |
| Djibouti | 21.4 (15.4-28.8) | 5.2 (3.7-7) | 138.8 (92.3-206.8) | 11 (7.3-16.4) | 2.46 (2.34-2.57) |
| Equatorial Guinea | 21.8 (14.5-31.7) | 5.2 (3.4-7.5) | 163 (93.5-262.3) | 10.8 (6.2-17.3) | 2.64 (2.45-2.82) |
| Rwanda | 514.8 (361.3-725.9) | 7.2 (5-10.1) | 1424.4 (989.5-1966) | 10.7 (7.5-14.8) | 0.96 (0.54-1.38) |
| Ghana | 923.4 (706.9-1174.8) | 6.2 (4.7-7.8) | 3584.5 (2641.9-4794.5) | 10.5 (7.7-14) | 1.4 (1.27-1.53) |
| Guatemala | 228.2 (212.1-244) | 2.7 (2.5-2.9) | 1642.4 (1375.6-1950.5) | 10.4 (8.7-12.4) | 4.49 (4.23-4.75) |
| Solomon Islands | 14.3 (8.5-20.8) | 4.2 (2.5-6.1) | 69.7 (48.7-96.6) | 10.2 (7.1-14.1) | 2.87 (2.67-3.06) |
| Eritrea | 183.8 (134.2-251) | 5.4 (3.9-7.4) | 658.5 (457.6-908.7) | 10 (6.9-13.8) | 1.98 (1.92-2.03) |
