# Supplementary material for: Global burden of breast cancer and attributable risk factors in 204 countries and territories, from 1990 to 2021: results from the Global Burden of Disease Study 2021
Source: Biomark Res. 2024 Aug 26;12:87. doi: 10.1186/s40364-024-00631-8 (PMC11346191; doi:10.1186/s40364-024-00631-8)
Supplement: Supplementary file 9 — Supplementary Material 9: Table S2. Deaths from Breast Cancerin 204 Countries, Globally and Regionally. [file 40364_2024_631_MOESM9_ESM.docx]

| **Table S2: Deaths from Breast Cancer (1990-2021) in 204 Countries, Globally and Regionally** | | | | | |
| --- | --- | --- | --- | --- | --- |
| **location** | **1990** | | **2021** | | **EAPC_95%CI** |
|  | **Num**ber(95%UI) | **ASR**(95%UI) | **Num**ber(95%UI) | **ASR**(95%UI) |  |
| Global | 355476.52 (335469.79-373476.11) | 6.66 (6.29-7) | 674199.41 (623371.55-720822.55) | 8.54 (7.9-9.13) | 0.69 (0.65-0.73) |
| High SDI | 144870.89 (136609.51-149554.95) | 16.47 (15.53-17) | 173263.69 (153063.01-183925.49) | 15.84 (13.99-16.81) | -0.07 (-0.14--0.01) |
| High-middle SDI | 94372.43 (89266.23-98922.85) | 8.87 (8.39-9.3) | 148441.93 (134917.54-161650.93) | 11.38 (10.35-12.4) | 0.87 (0.8-0.94) |
| Middle SDI | 65595.02 (60400.63-72165.34) | 3.81 (3.51-4.19) | 185770.72 (168485.96-206080.68) | 7.59 (6.88-8.42) | 2.04 (1.97-2.1) |
| Low-middle SDI | 34418.29 (30700.01-39033.02) | 2.96 (2.64-3.36) | 118455.95 (106614.65-129671.34) | 6.17 (5.55-6.75) | 2.24 (2.16-2.31) |
| Low SDI | 15712.01 (13462.1-18268.97) | 3.13 (2.69-3.64) | 47441.08 (41886.68-53334.82) | 4.25 (3.75-4.77) | 0.65 (0.52-0.79) |
| Andean Latin America | 1381.08 (1178.7-1602.62) | 3.64 (3.1-4.22) | 4117.68 (3256.73-5195.01) | 6.23 (4.92-7.86) | 1.73 (1.66-1.79) |
| Australasia | 3286.77 (3079.81-3478.5) | 16.21 (15.19-17.16) | 4367.82 (3749.66-4929.23) | 14.11 (12.11-15.92) | -0.2 (-0.3--0.09) |
| Caribbean | 2637.81 (2430.77-2879.78) | 7.47 (6.89-8.16) | 5687.07 (4808.05-6683.08) | 11.98 (10.13-14.08) | 1.67 (1.62-1.71) |
| Central Asia | 5169.81 (4869.64-5439.09) | 7.46 (7.03-7.85) | 6646.25 (5947.13-7417.23) | 6.94 (6.21-7.74) | -0.02 (-0.1-0.06) |
| Central Europe | 17648.17 (16921.4-18409.03) | 14.11 (13.53-14.72) | 25215.74 (22972.39-27335.49) | 21.88 (19.93-23.72) | 1.48 (1.42-1.54) |
| Central Latin America | 5647.22 (5477.04-5789.05) | 3.43 (3.33-3.52) | 19490.88 (17034.54-21941.86) | 7.7 (6.73-8.67) | 2.28 (2.18-2.39) |
| Central Sub-Saharan Africa | 1883.15 (1350.36-2520.62) | 3.43 (2.46-4.59) | 6361.46 (4732.88-8363.78) | 4.65 (3.46-6.11) | 0.7 (0.55-0.85) |
| East Asia | 42991.4 (35349.4-52009.47) | 3.53 (2.9-4.27) | 96400.77 (76551.86-118996.51) | 6.55 (5.2-8.08) | 1.65 (1.55-1.75) |
| Eastern Europe | 30026.28 (29059.64-30871.95) | 13.26 (12.83-13.63) | 36123.16 (32262.85-40813.59) | 17.47 (15.6-19.74) | 1.18 (0.94-1.42) |
| Eastern Sub-Saharan Africa | 7127.13 (5992.08-8623.02) | 3.73 (3.14-4.52) | 21339.82 (18273.74-25024.79) | 5.01 (4.29-5.87) | 0.51 (0.35-0.66) |
| High-income Asia Pacific | 7832.52 (7473.62-8077.65) | 4.52 (4.31-4.66) | 20226.94 (16845.54-22165.87) | 10.91 (9.08-11.95) | 2.98 (2.91-3.06) |
| High-income North America | 54016.8 (50562.49-56029.53) | 19.2 (17.97-19.91) | 59590.13 (53305.71-63192.5) | 16.1 (14.4-17.07) | -0.55 (-0.64--0.46) |
| North Africa and Middle East | 6979.93 (6266.59-7950.62) | 2.06 (1.85-2.34) | 30136.32 (26534.46-34261.2) | 4.84 (4.26-5.5) | 2.74 (2.56-2.92) |
| Oceania | 322.53 (251.27-406.11) | 4.92 (3.84-6.2) | 957.16 (784.27-1180.78) | 6.87 (5.63-8.48) | 0.93 (0.84-1.01) |
| South Asia | 30192.93 (26762.33-34106.85) | 2.76 (2.45-3.12) | 108084.94 (94490.36-123378.98) | 5.85 (5.12-6.68) | 2.26 (2.15-2.36) |
| Southeast Asia | 20339.06 (17044.26-24645.88) | 4.37 (3.66-5.29) | 66275.22 (55066.12-80748.49) | 9.49 (7.89-11.56) | 2.58 (2.55-2.61) |
| Southern Latin America | 7166.52 (6753.57-7506.16) | 14.47 (13.63-15.15) | 10083.86 (9079.72-10944.89) | 14.9 (13.41-16.17) | 0.52 (0.39-0.64) |
| Southern Sub-Saharan Africa | 2742.95 (2280.55-3219.05) | 5.23 (4.35-6.14) | 8552.8 (7743.5-9446.33) | 10.65 (9.64-11.76) | 2.23 (2.02-2.44) |
| Tropical Latin America | 8534.53 (8181.05-8847.88) | 5.59 (5.36-5.8) | 24416.53 (22597.8-25922.05) | 10.73 (9.93-11.39) | 2.1 (2.05-2.14) |
| Western Europe | 91971.05 (86193.06-95305.53) | 23.93 (22.42-24.79) | 93876.99 (80682.91-101172.82) | 21.46 (18.45-23.13) | -0.14 (-0.23--0.05) |
| Western Sub-Saharan Africa | 7578.89 (6220.01-8955.84) | 3.92 (3.22-4.64) | 26247.86 (20505.85-33460.93) | 5.36 (4.19-6.83) | 0.67 (0.52-0.83) |
| Côte d'Ivoire | 234.7 (192.6-282.5) | 11.8 (9.7-14.2) | 417.7 (324.9-523.8) | 19.2 (14.9-24.1) | 0 (0-0) |
| Maldives | 4.2 (2.2-7.2) | 1.9 (1-3.2) | 14.4 (11-18.2) | 2.8 (2.1-3.5) | 1.23 (1.04-1.43) |
| Myanmar | 2400.6 (1653.3-3405.3) | 5.9 (4.1-8.4) | 5317.6 (4105.2-7115.7) | 9.4 (7.3-12.6) | 0 (0-0) |
| Papua New Guinea | 170.8 (117.3-242.6) | 4.2 (2.9-5.9) | 583.8 (421.9-800.2) | 5.6 (4-7.6) | 0 (0-0) |
| Philippines | 3215.9 (2864.7-3555.4) | 5.1 (4.5-5.6) | 11381.6 (8978.8-14172.8) | 10.1 (7.9-12.5) | 2.04 (1.96-2.12) |
| Sri Lanka | 629.4 (524.8-748.7) | 3.7 (3.1-4.4) | 1779.3 (1165.7-2396.9) | 8 (5.2-10.8) | 0 (0-0) |
| Samoa | 7.7 (5.8-9.9) | 4.6 (3.4-5.9) | 16.1 (11.8-21.4) | 7.5 (5.5-10) | 0 (0-0) |
| Romania | 2656.6 (2489.7-2861.3) | 11.4 (10.6-12.2) | 4135.6 (3604.5-4719.3) | 21.8 (19-24.9) | 2.18 (2.12-2.24) |
| Mongolia | 32.6 (25.4-41.3) | 1.5 (1.2-1.9) | 84.8 (65.8-104.5) | 2.5 (2-3.1) | 1.61 (1.52-1.71) |
| Serbia | 1644.1 (1243.7-2105.1) | 17.1 (12.9-21.9) | 2342.1 (1783.3-3005.1) | 26.3 (20-33.7) | 1.32 (1.24-1.4) |
| Montenegro | 79 (60.8-102.6) | 12.6 (9.7-16.4) | 136 (106.3-173.1) | 22 (17.2-28) | 1.7 (1.56-1.85) |
| Belgium | 2936.7 (2667.7-3125.4) | 29.4 (26.7-31.3) | 2586 (2159.7-2910.7) | 22.5 (18.8-25.4) | 0 (0-0) |
| Solomon Islands | 9.6 (6-13.8) | 2.8 (1.8-4.1) | 44.4 (31.4-60.8) | 6.5 (4.6-8.9) | 2.45 (2.31-2.59) |
| Central African Republic | 109 (78.3-148) | 4 (2.9-5.4) | 254.2 (176.3-352) | 4.6 (3.2-6.4) | 0.23 (0.11-0.36) |
| Andorra | 6.7 (4.7-9.7) | 12.3 (8.6-17.9) | 14 (9.8-18.9) | 16.3 (11.5-22.1) | 0 (0-0) |
| Cyprus | 101.2 (82.5-122.3) | 13 (10.6-15.7) | 212.4 (175.1-256.4) | 15.6 (12.9-18.9) | 0 (0-0) |
| Cuba | 998.1 (938.6-1057.1) | 9.2 (8.7-9.7) | 1845.4 (1584.5-2152.3) | 16.4 (14.1-19.1) | 0 (0-0) |
| Argentina | 5436.7 (5085.7-5719.7) | 16.4 (15.4-17.3) | 7373.7 (6598.3-8042.3) | 16.2 (14.5-17.7) | 0 (0-0) |
| Yemen | 130.7 (82.1-184.2) | 1 (0.6-1.4) | 664.4 (460.7-944.3) | 2 (1.4-2.8) | 0 (0-0) |
| Russian Federation | 17353.9 (16830.3-17797.1) | 11.5 (11.1-11.8) | 25303.9 (22722.2-27819.5) | 17.5 (15.7-19.2) | 0 (0-0) |
| Tonga | 9.9 (7.9-12.4) | 10 (8-12.6) | 16.3 (12.1-21.4) | 15.4 (11.3-20.2) | 0 (0-0) |
| Thailand | 2371.3 (1921.8-2825.7) | 4.2 (3.4-5) | 9093.5 (6812.9-11497.8) | 13.6 (10.2-17.2) | 0 (0-0) |
| Dominica | 9.5 (8-11.1) | 13.2 (11-15.3) | 13.7 (10.4-17.1) | 20.4 (15.6-25.5) | 0 (0-0) |
| Slovakia | 701.7 (601.2-826.8) | 13.3 (11.4-15.7) | 1043 (781.4-1325.2) | 19.2 (14.4-24.4) | 0 (0-0) |
| Turkmenistan | 156.9 (136.9-178.1) | 4.2 (3.7-4.8) | 303.9 (226.2-406) | 5.9 (4.4-7.9) | 0 (0-0) |
| Botswana | 53.6 (37.4-74.3) | 4.1 (2.8-5.6) | 183.9 (131.6-260.1) | 7.7 (5.5-10.9) | 2.22 (2-2.44) |
| Denmark | 1734.6 (1617.1-1834.1) | 33.7 (31.4-35.7) | 1324.8 (1150.7-1460.9) | 22.6 (19.7-25) | 0 (0-0) |
| Slovenia | 319.9 (293.8-348.3) | 16.2 (14.9-17.6) | 431.1 (355.9-514) | 20.8 (17.2-24.8) | 0 (0-0) |
| Coted'Ivoire | 367.3 (284.5-471.1) | 3 (2.3-3.9) | 1374.5 (1002.7-1846.8) | 4.9 (3.6-6.6) | 0 (0-0) |
| Kenya | 576.6 (417.1-781.3) | 2.5 (1.8-3.4) | 2789.2 (1968.7-3836.7) | 5.6 (3.9-7.7) | 2.31 (2.15-2.47) |
| Tajikistan | 218.3 (175.3-265.3) | 4.1 (3.3-4.9) | 376.1 (236.7-553.5) | 3.7 (2.3-5.4) | -0.6 (-0.77--0.44) |
| Brunei Darussalam | 10.8 (8-14.2) | 4.1 (3.1-5.5) | 38.5 (29.9-47.2) | 8.5 (6.6-10.5) | 2.05 (1.74-2.36) |
| Finland | 907.6 (852.7-965.4) | 18.1 (17-19.3) | 1036.8 (888.6-1149) | 18.7 (16.1-20.8) | 0 (0-0) |
| Japan | 6605.1 (6281-6812.7) | 5.2 (5-5.4) | 16826 (13898.1-18483.5) | 13.2 (10.9-14.5) | 3.17 (3.08-3.26) |
| Cambodia | 381.6 (243.6-578.8) | 3.7 (2.4-5.6) | 1511.4 (1087.7-2008.7) | 8.9 (6.4-11.8) | 2.46 (2.29-2.64) |
| Uzbekistan | 896.1 (797.3-993.4) | 4.3 (3.8-4.7) | 1846.7 (1537.5-2214.2) | 5.4 (4.5-6.5) | 0 (0-0) |
| Kuwait | 34.2 (30.7-37.5) | 2 (1.8-2.2) | 165.3 (140.3-190.9) | 3.6 (3-4.1) | 1.27 (0.97-1.56) |
| Madagascar | 473 (380.8-585.2) | 4 (3.2-4.9) | 1263.9 (892.2-1693.7) | 4.4 (3.1-5.9) | 0 (0-0) |
| Austria | 1871.2 (1726.9-2002.6) | 24.1 (22.2-25.8) | 1730.2 (1483.3-1925.5) | 19.3 (16.5-21.4) | -0.36 (-0.46--0.27) |
| Vanuatu | 4.6 (3.2-6.5) | 3 (2.1-4.3) | 20.2 (14.8-26.2) | 6.4 (4.7-8.4) | 0 (0-0) |
| Lesotho | 67.3 (47.7-93.3) | 4.4 (3.1-6.1) | 169.4 (107.3-246.9) | 9 (5.7-13.2) | 2.65 (2.38-2.92) |
| Guyana | 44.2 (38.7-50.3) | 5.7 (5-6.5) | 90.9 (68.8-118.1) | 11.9 (9-15.4) | 2.54 (2.29-2.78) |
| Indonesia | 8000.8 (5502.6-11346.6) | 4.3 (3-6.1) | 25997.7 (17206.8-37660.4) | 9.3 (6.2-13.5) | 0 (0-0) |
| Chile | 974.1 (908.2-1043.7) | 7.3 (6.8-7.9) | 1776.4 (1581.3-1953.9) | 9.4 (8.4-10.4) | 1.13 (1.07-1.2) |
| Lao People's Democratic Republic | 164.2 (96.8-264.1) | 3.9 (2.3-6.3) | 488.3 (345.4-676.1) | 6.6 (4.7-9.2) | 0 (0-0) |
| Sierra Leone | 108 (78-142.7) | 2.6 (1.9-3.4) | 315.3 (224.2-422.7) | 3.6 (2.5-4.8) | 0.66 (0.57-0.76) |
| Ukraine | 9738.1 (9064.6-10412.3) | 18.5 (17.2-19.8) | 7628.6 (5022.4-10942.9) | 17.7 (11.7-25.4) | 0 (0-0) |
| Bolivia (Plurinational State of) | 283.6 (185.8-409.4) | 4.4 (2.9-6.4) | 890.3 (588.3-1291) | 7.5 (5-10.9) | 0 (0-0) |
| Uruguay | 755.4 (708.1-799.4) | 24.1 (22.6-25.5) | 933.2 (824.4-1032.3) | 27.4 (24.2-30.3) | 0.48 (0.39-0.57) |
| Haiti | 394.1 (239.5-610.9) | 6.2 (3.8-9.6) | 1079.7 (667.2-1674.6) | 8.4 (5.2-13) | 1.04 (1-1.08) |
| Ecuador | 267.4 (251.4-283.7) | 2.7 (2.5-2.8) | 1107.9 (864.2-1398) | 6.1 (4.8-7.7) | 2.92 (2.79-3.05) |
| Jamaica | 189.7 (175.1-204.8) | 8 (7.4-8.7) | 463.3 (352.5-584.7) | 16.5 (12.6-20.9) | 0 (0-0) |
| Venezuela (Bolivarian Republic of) | 788.6 (745.7-834.8) | 4.2 (4-4.4) | 3240.8 (2478.8-4093.4) | 12.2 (9.3-15.4) | 3.04 (2.89-3.19) |
| American Samoa | 3.2 (2.6-3.8) | 6.6 (5.4-7.9) | 9.8 (7.8-12.1) | 19.8 (15.6-24.4) | 0 (0-0) |
| Iceland | 40.2 (36.7-43.6) | 15.8 (14.5-17.2) | 51.9 (44.4-58.2) | 14.8 (12.7-16.6) | 0.11 (-0.02-0.25) |
| Puerto Rico | 365.5 (339.1-388.7) | 10.1 (9.4-10.8) | 591.8 (486-694.4) | 18 (14.8-21.1) | 0 (0-0) |
| Ireland | 704.6 (661.2-749.9) | 19.6 (18.4-20.8) | 722.1 (628.5-806.6) | 14.6 (12.7-16.3) | -0.48 (-0.62--0.33) |
| Latvia | 417.8 (374.3-469.1) | 15.7 (14.1-17.6) | 422.1 (349.9-493.8) | 22.6 (18.7-26.4) | 0 (0-0) |
| Albania | 105.4 (82.7-132.3) | 3.2 (2.5-4) | 229.5 (161.3-307.7) | 8.6 (6-11.5) | 0 (0-0) |
| Lithuania | 471.2 (432.2-509.9) | 12.8 (11.8-13.9) | 567.1 (470-669.7) | 20.8 (17.2-24.5) | 0 (0-0) |
| Canada | 4822.1 (4433.2-5138.5) | 17.7 (16.3-18.9) | 6110.7 (5298.5-6771.2) | 16.3 (14.1-18.1) | 0 (0-0) |
| Netherlands | 3728.6 (3360.8-3990.6) | 25 (22.5-26.7) | 3777 (3305.1-4196.8) | 21.9 (19.2-24.4) | -0.17 (-0.33--0.02) |
| Poland | 5060.1 (4856-5228.1) | 13.3 (12.7-13.7) | 8615 (7566.1-9569.5) | 22.5 (19.8-25) | 1.64 (1.57-1.72) |
| Australia | 2613.5 (2436.2-2779.4) | 15.5 (14.5-16.5) | 3606.1 (3090.7-4098.8) | 14 (12-15.9) | -0.12 (-0.22--0.01) |
| Italy | 13040 (12147.2-13601.3) | 23 (21.4-23.9) | 14640.9 (12142.2-16198.8) | 24.5 (20.3-27.1) | 0 (0-0) |
| Brazil | 8381.6 (8032.2-8696.5) | 5.6 (5.4-5.9) | 23850.7 (22086.8-25292.9) | 10.8 (10-11.5) | 0 (0-0) |
| Lebanon | 221.9 (160.3-296) | 7.4 (5.4-9.9) | 674.6 (540.8-831.8) | 12.2 (9.8-15) | 1.78 (1.59-1.97) |
| Fiji | 66.7 (52.7-84.4) | 8.8 (6.9-11.1) | 156.1 (114.5-205.8) | 16.9 (12.4-22.3) | 0 (0-0) |
| Israel | 768.7 (712.9-822.7) | 15.5 (14.4-16.6) | 1270 (1073.4-1412.2) | 13.2 (11.2-14.7) | -0.41 (-0.61--0.2) |
| New Zealand | 673.3 (624.8-718.7) | 19.7 (18.3-21) | 761.7 (657.6-840.6) | 14.7 (12.7-16.3) | 0 (0-0) |
| Congo | 138.3 (81.7-220.2) | 5.8 (3.4-9.2) | 470.7 (284-740.9) | 8.7 (5.3-13.7) | 0 (0-0) |
| Saint Kitts and Nevis | 8.1 (7.5-8.9) | 19.6 (18-21.4) | 10.8 (8.9-12.8) | 18.4 (15.2-21.9) | 0 (0-0) |
| Bermuda | 12.7 (11.4-13.9) | 21.3 (19.3-23.4) | 15.1 (12.4-19) | 23.8 (19.5-29.9) | 0 (0-0) |
| Togo | 97.7 (79.2-122.8) | 2.7 (2.2-3.4) | 413.8 (296-568.3) | 4.9 (3.5-6.8) | 0 (0-0) |
| Dominican Republic | 239.6 (201.8-281.4) | 3.4 (2.8-3.9) | 755.5 (579.7-980.5) | 6.9 (5.3-8.9) | 0 (0-0) |
| Peru | 830.1 (686.1-985.9) | 3.8 (3.2-4.6) | 2119.5 (1537.2-2834.2) | 5.8 (4.2-7.8) | 0 (0-0) |
| Kiribati | 5 (3.9-6.4) | 6.8 (5.2-8.6) | 13.7 (10.3-18.6) | 11.3 (8.5-15.4) | 1.55 (1.48-1.62) |
| United States of America | 49187.6 (45894.2-50998.8) | 19.4 (18.1-20.1) | 53472.8 (47932.9-56790.7) | 16.1 (14.4-17.1) | 0 (0-0) |
| Norway | 855.4 (789.3-898.4) | 20.1 (18.6-21.2) | 735.7 (643.1-806.1) | 13.6 (11.9-14.9) | 0 (0-0) |
| Costa Rica | 118.4 (109.3-126.8) | 3.9 (3.6-4.2) | 492.8 (428.3-559.5) | 10.4 (9-11.8) | 0 (0-0) |
| Trinidad and Tobago | 119.4 (111.1-128.6) | 9.9 (9.2-10.7) | 257.5 (198.1-325.5) | 18.5 (14.2-23.4) | 0 (0-0) |
| Azerbaijan | 507.8 (424.6-577.4) | 6.9 (5.8-7.9) | 867.9 (651.1-1117.1) | 8.3 (6.2-10.6) | 0.49 (0.36-0.63) |
| Marshall Islands | 1.9 (1.4-2.7) | 4.2 (3-5.9) | 6 (3.6-9.3) | 10.6 (6.3-16.5) | 2.92 (2.84-3) |
| El Salvador | 132.9 (116-151.1) | 2.5 (2.2-2.8) | 422.2 (334.8-522) | 6.5 (5.2-8.1) | 3.12 (3.02-3.21) |
| Portugal | 1827.7 (1706.5-1947.1) | 18 (16.8-19.2) | 2103.4 (1819.2-2317.3) | 19.8 (17.1-21.8) | 0 (0-0) |
| Malawi | 299.4 (237.3-368.5) | 3.1 (2.4-3.8) | 902.1 (652.1-1191.8) | 4.6 (3.4-6.1) | 0 (0-0) |
| Namibia | 67.6 (55.9-82.5) | 4.8 (4-5.9) | 267.2 (174.9-370.6) | 11 (7.2-15.2) | 0 (0-0) |
| China | 41217.7 (33621.1-50194.4) | 3.5 (2.9-4.3) | 91483.8 (71738.6-113710.5) | 6.4 (5-8) | 1.58 (1.48-1.69) |
| Gambia | 11.7 (8.7-15.5) | 1.2 (0.9-1.6) | 55.8 (40.3-74.5) | 2.3 (1.7-3.1) | 0 (0-0) |
| Luxembourg | 99.7 (93.2-106.2) | 26.2 (24.4-27.9) | 104.1 (91.4-116.2) | 16.2 (14.2-18) | 0 (0-0) |
| Libya | 83.5 (66.1-106.6) | 2 (1.6-2.5) | 396.9 (293.6-545.7) | 5.8 (4.3-7.9) | 3.3 (3.19-3.42) |
| South Africa | 2166.8 (1733.3-2610.4) | 5.9 (4.7-7.1) | 6556 (5932.5-7261.3) | 11.5 (10.4-12.8) | 0 (0-0) |
| Ghana | 626.3 (483.9-794.9) | 4.2 (3.2-5.3) | 2037.5 (1525.3-2712.2) | 5.9 (4.5-7.9) | 1.25 (1.13-1.36) |
| Oman | 14.2 (10.3-19.3) | 0.7 (0.5-1) | 47.6 (36-61.5) | 1 (0.8-1.3) | 1.45 (1.21-1.69) |
| Georgia | 959.7 (867.2-1058.9) | 17.4 (15.7-19.2) | 948.3 (818.5-1088.1) | 26.3 (22.7-30.2) | 0 (0-0) |
| Qatar | 12.3 (9.8-15.4) | 2.8 (2.2-3.5) | 82.4 (59.6-112.6) | 2.8 (2-3.8) | 0 (0-0) |
| Timor-Leste | 16.2 (10.2-24.7) | 2.1 (1.3-3.2) | 60.4 (41.5-82.4) | 4.3 (3-5.9) | 2.24 (2.08-2.41) |
| Cook Islands | 2.4 (1.9-3) | 12.6 (9.8-16) | 5.1 (3.8-6.4) | 28.5 (21.7-36.2) | 2.23 (2-2.46) |
| Mauritius | 48.6 (45.4-52.3) | 4.4 (4.1-4.8) | 224.6 (203.5-239.2) | 17.7 (16-18.8) | 4.06 (3.74-4.39) |
| Kazakhstan | 1638.4 (1445.4-1828.5) | 10 (8.8-11.2) | 1380.2 (1145.6-1607) | 7.3 (6-8.5) | 0 (0-0) |
| Bhutan | 10.5 (7.3-13.7) | 1.7 (1.2-2.2) | 27.4 (19.4-37.2) | 3.6 (2.6-4.9) | 0 (0-0) |
| Burundi | 237.3 (169.3-333.8) | 4.3 (3-6) | 438.8 (320.1-600.2) | 3.3 (2.4-4.5) | 0 (0-0) |
| India | 22510.7 (19225.9-26349.5) | 2.6 (2.3-3.1) | 80817.2 (68415.7-95686.7) | 5.7 (4.8-6.8) | 0 (0-0) |
| Saudi Arabia | 208 (151-282.6) | 1.3 (1-1.8) | 1223.3 (867.3-1703.7) | 3.2 (2.3-4.5) | 0 (0-0) |
| Democratic Republic of the Congo | 1251.8 (876.9-1714.7) | 3.3 (2.3-4.5) | 3995 (2932.1-5514) | 4.4 (3.3-6.1) | 0 (0-0) |
| San Marino | 3.8 (2.9-4.8) | 16.1 (12.4-20.3) | 4.4 (2.6-6.3) | 13.3 (8.1-19.3) | 0 (0-0) |
| Morocco | 453.5 (353.5-582.8) | 1.8 (1.4-2.3) | 1768.1 (1190.5-2541.8) | 4.8 (3.2-6.8) | 0 (0-0) |
| Malta | 80.9 (74.7-87.6) | 21.8 (20.2-23.6) | 99.4 (84.1-113.4) | 22.5 (19-25.7) | 0 (0-0) |
| Greenland | 5.8 (4.7-7.3) | 10.5 (8.4-13.1) | 5.8 (4.3-7.5) | 10.3 (7.7-13.5) | 0 (0-0) |
| Nicaragua | 56.7 (48.3-66.1) | 1.5 (1.2-1.7) | 248.9 (193.8-312.1) | 3.7 (2.9-4.7) | 3.18 (3.04-3.32) |
| Panama | 91.1 (84.3-97.4) | 3.8 (3.5-4.1) | 332.2 (263.9-398.4) | 7.7 (6.1-9.3) | 2.27 (2.16-2.39) |
| Algeria | 452.3 (346.7-582.6) | 1.8 (1.4-2.3) | 1541.1 (1173.7-1982.3) | 3.5 (2.7-4.5) | 0 (0-0) |
| Comoros | 19.6 (14.2-26.5) | 4.2 (3.1-5.7) | 66.7 (49.8-89.5) | 9 (6.7-12) | 0 (0-0) |
| Djibouti | 14.9 (10.9-20) | 3.6 (2.6-4.8) | 82.3 (55-120.7) | 6.5 (4.4-9.6) | 0 (0-0) |
| Nepal | 399.2 (295.1-519.5) | 2.1 (1.5-2.7) | 1165.3 (833.1-1615.3) | 3.7 (2.7-5.2) | 1.46 (1.18-1.74) |
| Seychelles | 4.8 (4.2-5.6) | 6.6 (5.7-7.7) | 13.6 (11.5-15.8) | 12.9 (10.9-15) | 0 (0-0) |
| Somalia | 216.4 (147.4-299.4) | 2.7 (1.9-3.8) | 577.1 (390.2-800.7) | 2.7 (1.8-3.7) | 0 (0-0) |
| Equatorial Guinea | 17 (11.4-24.4) | 4 (2.7-5.8) | 82 (48.6-129.9) | 5.4 (3.2-8.6) | 0 (0-0) |
| Colombia | 1423.6 (1333-1513.6) | 4.4 (4.1-4.7) | 4234 (3541-5040.3) | 8.6 (7.2-10.3) | 0 (0-0) |
| Tokelau | 0.2 (0.1-0.3) | 11.4 (7.6-16.1) | 0.2 (0.2-0.3) | 17.6 (12.9-23.3) | 0 (0-0) |
| Mali | 318.6 (258.6-384.3) | 3.7 (3-4.4) | 827.8 (593.1-1138.4) | 3.4 (2.5-4.7) | 0 (0-0) |
| Grenada | 10.4 (9.4-11.4) | 11.9 (10.8-13.1) | 18.6 (16.3-21.1) | 18.2 (15.9-20.5) | 0 (0-0) |
| Czechia | 1948.5 (1757.4-2174.9) | 18.9 (17.1-21.1) | 1982.8 (1647.4-2330.6) | 18.6 (15.5-21.9) | 0.18 (0-0.35) |
| Mauritania | 78.7 (56.8-106.3) | 3.8 (2.8-5.2) | 209.8 (158.4-278.2) | 4.8 (3.6-6.3) | 0.52 (0.42-0.63) |
| Tuvalu | 0.9 (0.6-1.2) | 9 (5.9-13) | 1.5 (1.1-2.1) | 12.4 (9.1-16.8) | 1.34 (1.2-1.49) |
| Benin | 125.2 (102.2-150.7) | 2.6 (2.1-3.1) | 406.2 (298.6-538.9) | 3 (2.2-4) | 0 (0-0) |
| Hungary | 2152.6 (1916.4-2415) | 20.7 (18.4-23.2) | 2222.8 (1888.4-2575.6) | 23.2 (19.7-26.8) | 0.62 (0.46-0.77) |
| United Republic of Tanzania | 1051.4 (848.2-1285.6) | 4.1 (3.3-5) | 3300.5 (2440.9-4390.3) | 5.6 (4.2-7.5) | 0.84 (0.75-0.93) |
| Democratic People's Republic of Korea | 992.2 (656-1437.5) | 4.8 (3.2-7) | 2072.5 (1449.9-2765) | 7.9 (5.5-10.5) | 0 (0-0) |
| United Arab Emirates | 44.1 (32.5-58.7) | 2.4 (1.7-3.1) | 335.2 (241.6-448.9) | 3.5 (2.5-4.7) | 0 (0-0) |
| Niger | 131.2 (98.5-176.1) | 1.6 (1.2-2.2) | 443.4 (305.7-610.4) | 1.8 (1.2-2.4) | 0.1 (0.04-0.16) |
| Afghanistan | 335.9 (177.1-561.9) | 3.4 (1.8-5.7) | 941.4 (488.9-1679.5) | 3 (1.6-5.4) | -0.61 (-0.92--0.29) |
| Viet Nam | 1997.7 (1555.7-2586) | 2.9 (2.3-3.8) | 6400.6 (4842-8457.4) | 6.4 (4.8-8.4) | 2.29 (2.15-2.44) |
| Palestine | 87.8 (63.7-121.5) | 4.3 (3.1-5.9) | 300.7 (242.3-368.7) | 5.9 (4.7-7.2) | 0 (0-0) |
| Uganda | 660.7 (481.4-879) | 3.8 (2.8-5.1) | 2485.4 (1834.1-3320.4) | 5.7 (4.2-7.7) | 0 (0-0) |
| Monaco | 12.2 (8.8-16.2) | 40.2 (28.8-53.2) | 17.5 (13.3-23) | 46.2 (35.2-60.7) | 0.36 (0.29-0.43) |
| Burkina Faso | 459.4 (353.2-581.8) | 4.8 (3.7-6.1) | 1167.7 (844.9-1554.7) | 5.1 (3.7-6.8) | 0 (0-0) |
| United Kingdom | 17643.7 (16628.8-18138.9) | 30.8 (29-31.7) | 13914.5 (12358-14739.1) | 20.5 (18.2-21.7) | 0 (0-0) |
| Nigeria | 4197.3 (3176.4-5415.9) | 4.7 (3.5-6) | 15668.5 (10782.3-22256) | 6.8 (4.7-9.6) | 0 (0-0) |
| Barbados | 49.1 (44.8-53.1) | 19.4 (17.7-21) | 96.2 (77.9-118.8) | 32.2 (26.1-39.7) | 0 (0-0) |
| Cameroon | 371.4 (297.6-461) | 3.6 (2.9-4.4) | 1332.9 (954.8-1824.8) | 4.2 (3-5.7) | 0 (0-0) |
| Iraq | 536.6 (405.8-704.5) | 2.9 (2.2-3.8) | 2309.4 (1629.6-3101.8) | 5.6 (4-7.5) | 0 (0-0) |
| Sudan | 279.5 (177.3-423.5) | 1.4 (0.9-2.1) | 945.4 (582.4-1507.3) | 2.2 (1.3-3.5) | 0.97 (0.76-1.19) |
| Paraguay | 152.9 (123.9-185.2) | 3.8 (3.1-4.6) | 565.8 (419.6-755.3) | 7.9 (5.9-10.5) | 2.46 (2.35-2.56) |
| Guinea | 219.2 (164.3-275.4) | 3.7 (2.7-4.6) | 529.8 (376.9-737.9) | 3.9 (2.8-5.5) | 0.35 (0.28-0.43) |
| Angola | 301.1 (215.2-417.1) | 2.9 (2.1-4.1) | 1401.5 (952.1-1900.1) | 4.3 (2.9-5.8) | 0 (0-0) |
| Belize | 4.7 (4.3-5.2) | 2.5 (2.3-2.8) | 22.2 (19.5-25) | 5.2 (4.5-5.8) | 2.13 (1.96-2.3) |
| Jordan | 110.9 (84.6-142.7) | 3 (2.3-3.8) | 621.9 (442.1-835.2) | 5 (3.6-6.8) | 0 (0-0) |
| Nauru | 0.7 (0.4-1.2) | 7.3 (4.4-11.4) | 1.3 (0.8-2) | 12 (7.1-18.5) | 0 (0-0) |
| Niue | 0.4 (0.3-0.5) | 15.2 (11.8-19.6) | 0.4 (0.3-0.5) | 23.1 (17.5-29.8) | 0 (0-0) |
| Gabon | 66.1 (47.5-88.5) | 6.7 (4.8-9) | 158 (108.2-217.1) | 8.7 (6-12) | 0 (0-0) |
| Guinea-Bissau | 35.1 (24.3-50.9) | 3.5 (2.4-5.1) | 93.9 (65.2-128.5) | 4.6 (3.2-6.2) | 0 (0-0) |
| South Sudan | 180.5 (130-256.3) | 3.1 (2.2-4.4) | 371.6 (260.7-532.1) | 3.8 (2.7-5.5) | 0 (0-0) |
| Mozambique | 491.6 (405.9-598) | 3.7 (3-4.5) | 1446.8 (1014.9-1927.9) | 4.7 (3.3-6.2) | 0 (0-0) |
| Saint Lucia | 14.1 (13.1-15.2) | 10.3 (9.6-11.1) | 29.7 (24.8-35.7) | 16.7 (14-20.1) | 0 (0-0) |
| Eswatini | 28.7 (21.9-36.8) | 3.6 (2.7-4.6) | 90.5 (52.6-141) | 7.8 (4.6-12.2) | 0 (0-0) |
| Liberia | 66.5 (52.4-82.2) | 2.7 (2.1-3.3) | 196.8 (135.6-285.2) | 3.6 (2.5-5.2) | 0 (0-0) |
| Taiwan (Province of China) | 781.5 (738.5-823) | 3.8 (3.6-4) | 2844.4 (2564.5-3101.1) | 12 (10.8-13.1) | 0 (0-0) |
| Bosnia and Herzegovina | 319.5 (272.9-360.1) | 7.1 (6.1-8) | 577 (457.7-710.4) | 17.5 (13.9-21.5) | 0 (0-0) |
| Rwanda | 400.4 (283.9-560.5) | 5.6 (3.9-7.8) | 888.3 (624.4-1218.8) | 6.7 (4.7-9.2) | 0 (0-0) |
| Republic of Korea | 1020.3 (896.3-1212.3) | 2.3 (2-2.7) | 2879 (2297.4-3467.3) | 5.6 (4.5-6.7) | 0 (0-0) |
| Bangladesh | 1638.6 (1232.3-2218.1) | 1.5 (1.1-2) | 5764.8 (4383.2-7409.2) | 3.5 (2.7-4.5) | 0 (0-0) |
| Bahrain | 22.3 (18.7-26) | 4.4 (3.7-5.1) | 98.8 (76.6-127.8) | 6.5 (5-8.4) | 0 (0-0) |
| Singapore | 196.4 (185.1-208.6) | 6.4 (6.1-6.8) | 483.5 (436.4-523.7) | 8.4 (7.6-9.1) | 0 (0-0) |
| Armenia | 454.1 (420.8-485.9) | 13.3 (12.3-14.2) | 503 (440.5-583.5) | 16.8 (14.7-19.5) | 1.76 (1.42-2.1) |
| Belarus | 1247.8 (1135.7-1361.2) | 11.9 (10.9-13) | 1362.9 (1084.9-1697.2) | 14.6 (11.6-18.2) | 0 (0-0) |
| Estonia | 253.1 (233.4-273) | 16.1 (14.9-17.4) | 247.9 (200.5-295.7) | 18.9 (15.3-22.6) | 0 (0-0) |
| France | 12971.1 (12006.5-13780) | 22.5 (20.8-23.9) | 15358.1 (12964.9-17078) | 23.1 (19.5-25.7) | 0.23 (0.17-0.29) |
| Bulgaria | 1312.6 (1152.1-1496.4) | 15.1 (13.3-17.2) | 1758.5 (1440.2-2080.4) | 25.9 (21.2-30.7) | 0 (0-0) |
| Croatia | 831.2 (734.7-938.4) | 17.1 (15.1-19.3) | 957.5 (819.5-1106.1) | 22.8 (19.5-26.3) | 0 (0-0) |
| Antigua and Barbuda | 7.8 (7-8.7) | 12.9 (11.6-14.4) | 17.7 (16.5-19) | 19.7 (18.5-21.3) | 0 (0-0) |
| Guam | 6.6 (5.6-7.7) | 4.8 (4.1-5.6) | 14.2 (11.7-17.4) | 8.9 (7.3-11) | 0 (0-0) |
| Zimbabwe | 358.9 (276.8-462) | 3.5 (2.7-4.5) | 1285.7 (928.3-1719.7) | 8.2 (6-11) | 2.97 (2.53-3.41) |
| United States Virgin Islands | 14.3 (12-17) | 13.5 (11.3-16) | 18.4 (12.7-25.4) | 21.4 (14.8-29.6) | 1.78 (1.67-1.9) |
| Bahamas | 32.2 (29.5-35.2) | 12.5 (11.5-13.7) | 85.5 (69.5-105.3) | 22 (17.9-27.1) | 0 (0-0) |
| Germany | 20718.6 (19236.2-22034.9) | 25.9 (24.1-27.6) | 20860.9 (17921.4-23045.9) | 24.4 (21-27) | -0.04 (-0.11-0.03) |
| Malaysia | 1074.3 (909.7-1253.4) | 6.1 (5.1-7.1) | 3899.6 (3318.8-4535.3) | 12.3 (10.4-14.3) | 0 (0-0) |
| Spain | 6748.1 (6183.5-7233.4) | 17.4 (15.9-18.7) | 7213.6 (6084.4-8149.7) | 15.8 (13.4-17.9) | 0 (0-0) |
| Micronesia (Federated States of) | 6.7 (4.6-9.4) | 6.5 (4.5-9) | 13.4 (9.7-17.7) | 13.1 (9.4-17.3) | 0 (0-0) |
| Greece | 1998.3 (1863.4-2111.9) | 19.2 (17.9-20.3) | 2907.5 (2487.7-3187.8) | 28.6 (24.4-31.3) | 0 (0-0) |
| Saint Vincent and the Grenadines | 11.4 (10.3-12.3) | 10.4 (9.4-11.2) | 21.1 (18.4-24) | 18.5 (16.1-21.1) | 1.95 (1.8-2.1) |
| Suriname | 23.7 (20.1-27.5) | 6.1 (5.2-7.1) | 61.7 (47.2-78.7) | 10.7 (8.2-13.6) | 0 (0-0) |
| Sweden | 1669.1 (1535-1789.2) | 19.4 (17.9-20.8) | 1614 (1336.4-1853.6) | 15.6 (12.9-17.9) | 0 (0-0) |
| Syrian Arab Republic | 255.9 (195-319.2) | 2 (1.5-2.5) | 772.6 (561.5-1039.4) | 5.5 (4-7.4) | 0 (0-0) |
| Guatemala | 114.3 (107-120.9) | 1.4 (1.3-1.4) | 548.5 (460.2-639.6) | 3.5 (2.9-4.1) | 0 (0-0) |
| Tunisia | 245 (199.6-295.5) | 2.9 (2.4-3.5) | 782.5 (562.6-1075.3) | 6.6 (4.8-9.1) | 0 (0-0) |
| Honduras | 91.1 (64.9-119.2) | 1.9 (1.4-2.5) | 473.4 (341.1-651.5) | 4.7 (3.4-6.4) | 0 (0-0) |
| Turkey | 1118 (890.3-1383.2) | 1.9 (1.5-2.4) | 6370.8 (4987.5-7927.1) | 7.6 (6-9.5) | 4.74 (4.18-5.29) |
| Mexico | 2830.5 (2746.5-2909.1) | 3.3 (3.2-3.4) | 9498.1 (7993-11092.6) | 7.3 (6.2-8.6) | 2.23 (2.14-2.31) |
| Switzerland | 1426.6 (1313.4-1521.9) | 20.8 (19.1-22.2) | 1495.4 (1249.9-1685.1) | 16.8 (14-18.9) | -0.56 (-0.65--0.47) |
| Cabo Verde | 17.8 (14.5-21.5) | 5 (4.1-6.1) | 38.4 (29.7-48.5) | 6.9 (5.3-8.7) | 0.52 (0.2-0.84) |
| Zambia | 286.7 (208.7-394.9) | 3.6 (2.6-5) | 1396.1 (811.8-2168.9) | 7.2 (4.2-11.1) | 0 (0-0) |
| Chad | 141.9 (103-186.1) | 2.4 (1.7-3.1) | 394.8 (282.9-522.8) | 2.2 (1.6-2.9) | 0 (0-0) |
| Northern Mariana Islands | 2.5 (1.8-3.3) | 5.5 (3.9-7.3) | 7.3 (6-8.5) | 15 (12.4-17.5) | 0 (0-0) |
| Palau | 2.2 (1.7-2.8) | 14.2 (10.9-18.4) | 4.4 (3.4-5.6) | 24.2 (18.5-31) | 1.5 (1.36-1.64) |
| Sao Tome and Principe | 3.8 (3.1-4.6) | 3.1 (2.5-3.8) | 10.9 (8.2-14.2) | 5 (3.8-6.6) | 0 (0-0) |
| Pakistan | 5634.1 (4440.1-7033.7) | 5.1 (4-6.3) | 20310.3 (14669.2-26959.6) | 8.6 (6.2-11.4) | 1.97 (1.76-2.18) |
| Egypt | 1403 (1220.5-1672.9) | 2.5 (2.2-3) | 6000.4 (4761.1-7431.4) | 5.7 (4.5-7) | 2.75 (2.55-2.95) |
| Senegal | 201.8 (159.2-253.1) | 2.6 (2.1-3.3) | 729.8 (551.9-969.7) | 4.6 (3.5-6.1) | 0 (0-0) |
| Iran (Islamic Republic of) | 926.6 (796.7-1066.7) | 1.6 (1.4-1.9) | 4065.3 (3668.5-4501.8) | 4.8 (4.3-5.3) | 0 (0-0) |
| Eritrea | 137.9 (100.2-188.5) | 4.1 (2.9-5.5) | 437.2 (306.9-600.2) | 6.6 (4.7-9.1) | 1.49 (1.45-1.53) |
| Kyrgyzstan | 306 (276.3-339.1) | 6.9 (6.2-7.6) | 335.2 (274.1-399.4) | 4.9 (4-5.8) | 0 (0-0) |
| Ethiopia | 2075.6 (1382.7-3050) | 4.1 (2.7-6) | 4875.4 (3987.7-5981.5) | 4.5 (3.7-5.5) | -0.42 (-0.65--0.19) |
| Republic of Moldova | 544.4 (486.5-598.9) | 12.2 (10.9-13.5) | 590.6 (500.9-692.7) | 16.4 (13.9-19.3) | 1.1 (0.92-1.28) |
