# Supplementary material for: Global burden of breast cancer and attributable risk factors in 204 countries and territories, from 1990 to 2021: results from the Global Burden of Disease Study 2021
Source: Biomark Res. 2024 Aug 26;12:87. doi: 10.1186/s40364-024-00631-8 (PMC11346191; doi:10.1186/s40364-024-00631-8)
Supplement: Supplementary file 10 — Supplementary Material 10: Table S3. Disabdeathsility-adjusted life years from Breast Cancerin 204 Countries, Globally and Regionally. [file 40364_2024_631_MOESM10_ESM.docx]

| **Table S3: Disability-adjusted life years from Breast Cancer (1990-2021) in 204 Countries, Globally and Regionally** | | | | | |
| --- | --- | --- | --- | --- | --- |
| Location | 1990 | | 2021 | | **EAPC_95%CI** |
|  | **Num**ber(95%UI) | **ASR**(95%UI) | **Num**ber(95%UI) | **ASR**(95%UI) |  |
| Global | 11181358.25 (10581120.5-11817721.64) | 209.64 (198.39-221.57) | 20635718.18 (19358110.66-21993502.55) | 261.5 (245.31-278.7) | 0.63 (0.58-0.69) |
| High SDI | 4062051.4 (3895031.43-4214225.65) | 461.84 (442.85-479.14) | 4367549.74 (4024277.32-4651990.05) | 399.21 (367.83-425.21) | -0.56 (-0.59--0.52) |
| High-middle SDI | 2959509.02 (2789757.28-3120710.92) | 278.27 (262.31-293.43) | 4262981.58 (3900199.84-4726495.12) | 326.91 (299.09-362.45) | 0.38 (0.31-0.45) |
| Middle SDI | 2336382.79 (2137453.57-2577699.6) | 135.61 (124.06-149.61) | 6169416.01 (5577685.35-6827006.16) | 251.96 (227.8-278.82) | 1.97 (1.93-2) |
| Low-middle SDI | 1255193.8 (1120606.67-1429855.3) | 108.07 (96.49-123.11) | 4135798.31 (3730357.23-4539759.4) | 215.28 (194.18-236.31) | 2.27 (2.19-2.34) |
| Low SDI | 552836.53 (469209.39-649572.67) | 110.28 (93.6-129.58) | 1677080.08 (1468416.78-1891533.01) | 150.09 (131.42-169.28) | 0.91 (0.72-1.11) |
| Andean Latin America | 46873.02 (39800.74-54742.32) | 123.37 (104.76-144.08) | 129548.81 (102648.38-163266.33) | 195.89 (155.21-246.87) | 1.38 (1.28-1.48) |
| Australasia | 95124.12 (89946.34-100262.73) | 469.14 (443.6-494.48) | 113410.87 (101203.7-126349.83) | 366.3 (326.87-408.09) | -0.87 (-0.93--0.81) |
| Caribbean | 83317.9 (75861.48-92258.89) | 236.08 (214.95-261.41) | 166676.24 (139162.68-197643.25) | 351.2 (293.23-416.45) | 1.41 (1.35-1.47) |
| Central Asia | 172904.79 (163423.98-181885.08) | 249.46 (235.78-262.42) | 219258.84 (194880.89-246457.27) | 228.85 (203.41-257.24) | -0.04 (-0.11-0.03) |
| Central Europe | 521026.6 (499220.04-544350.19) | 416.51 (399.08-435.16) | 609777.79 (562489.8-659711.4) | 529.03 (488-572.35) | 0.67 (0.57-0.77) |
| Central Latin America | 195825.52 (190418.34-201652.09) | 119.11 (115.82-122.65) | 636255.21 (550996.42-717860.75) | 251.48 (217.78-283.74) | 2.33 (2.23-2.44) |
| Central Sub-Saharan Africa | 66507.45 (47322.74-90058.36) | 121 (86.1-163.85) | 225986.72 (167663.42-298098.33) | 165.04 (122.45-217.7) | 1.01 (0.78-1.25) |
| East Asia | 1560396.88 (1272690.44-1892837.4) | 128.17 (104.54-155.48) | 3187710.8 (2516807.75-4001378.22) | 216.44 (170.89-271.69) | 1.59 (1.5-1.69) |
| Eastern Europe | 944775.85 (913329.27-975124.97) | 417.14 (403.25-430.54) | 990432.65 (884266.81-1123187.17) | 479.03 (427.68-543.23) | -0.02 (-0.24-0.2) |
| Eastern Sub-Saharan Africa | 248726.26 (205694.57-303784.59) | 130.34 (107.79-159.2) | 749841.5 (627813.45-899054.35) | 175.98 (147.34-211) | 0.83 (0.61-1.04) |
| High-income Asia Pacific | 284460.91 (272677.56-297671.1) | 164.07 (157.27-171.69) | 542175.32 (483246.78-588128.45) | 292.36 (260.59-317.14) | 1.86 (1.71-2.02) |
| High-income North America | 1559200.03 (1484608.28-1631077.86) | 554.07 (527.56-579.61) | 1573540.33 (1465780.85-1678354.83) | 425.08 (395.97-453.4) | -0.96 (-1.03--0.9) |
| North Africa and Middle East | 258054.04 (228602.59-298393.82) | 76.08 (67.4-87.97) | 1083820.37 (944244.34-1245184.7) | 173.97 (151.56-199.87) | 3.08 (2.9-3.26) |
| Oceania | 12311.4 (9475.1-15563.78) | 187.96 (144.66-237.62) | 36209.01 (29094.12-46122.15) | 259.98 (208.9-331.16) | 1.1 (0.98-1.21) |
| South Asia | 1125384.27 (999518.35-1268172.82) | 102.92 (91.41-115.98) | 3781141.03 (3308591.5-4332368.78) | 204.77 (179.17-234.62) | 2.19 (2.03-2.35) |
| Southeast Asia | 739742.53 (616179.99-897565.11) | 158.91 (132.37-192.81) | 2255795.58 (1865830.05-2764870.76) | 323.04 (267.19-395.94) | 2.31 (2.25-2.38) |
| Southern Latin America | 205052.38 (194856.54-214368.59) | 413.93 (393.34-432.73) | 263672.63 (242740.73-283345.93) | 389.5 (358.58-418.57) | -0.14 (-0.21--0.06) |
| Southern Sub-Saharan Africa | 91354.75 (77461.71-105721.9) | 174.28 (147.78-201.69) | 271585.07 (242598.56-303051.15) | 338.2 (302.1-377.38) | 2.73 (2.44-3.02) |
| Tropical Latin America | 285349.89 (275269.17-295096.93) | 187.05 (180.44-193.44) | 759253.75 (713098.74-797995.16) | 333.7 (313.42-350.73) | 1.71 (1.65-1.76) |
| Western Europe | 2437316.88 (2336303.84-2525031.62) | 634.04 (607.76-656.85) | 2147815.2 (1938627.61-2312415.94) | 491.05 (443.23-528.69) | -0.88 (-0.93--0.84) |
| Western Sub-Saharan Africa | 247652.82 (201250.37-295486.51) | 128.21 (104.19-152.98) | 891810.46 (669024.99-1159840.08) | 182.07 (136.58-236.79) | 1.18 (1-1.36) |
| Côte d'Ivoire | 7824.2 (6417.1-9382.4) | 392.7 (322.1-470.9) | 11548.4 (8926.8-14624.4) | 361.4 (279.1-459.1) | 0 (0-0) |
| Maldives | 155.1 (77-272.1) | 69.8 (34.7-122.4) | 490.5 (365.7-638.8) | 108.5 (82.4-138.4) | -0.95 (-1.27--0.64) |
| Myanmar | 92799.2 (62159.5-134243.3) | 229.5 (153.7-332) | 186706 (139264.6-251573.7) | 336.3 (252.9-451.8) | -0.05 (-0.17-0.07) |
| Papua New Guinea | 6775.3 (4564.6-9664.3) | 165.1 (111.2-235.5) | 23480.7 (16719.9-32385.6) | 305.2 (221-416.4) | 0 (0-0) |
| Philippines | 114433.1 (101769.1-125751.5) | 181.6 (161.5-199.6) | 393980.9 (306468.7-495793.5) | 403.7 (316.4-504.6) | 0.92 (0.86-0.99) |
| Sri Lanka | 21929.8 (18305.1-26189.4) | 128 (106.9-152.9) | 53726.8 (34763.2-73543) | 199.4 (129.1-271.8) | 0.81 (0.64-0.97) |
| Samoa | 248.7 (183.8-322.1) | 147.2 (108.8-190.7) | 520.6 (377.2-698.3) | 324.9 (236-432.8) | 0.64 (0.57-0.71) |
| Romania | 84842.6 (79123.8-91639.9) | 362.9 (338.4-392) | 104227.5 (90649.6-117872.9) | 320.9 (279.3-361.9) | -0.03 (-0.14-0.09) |
| Mongolia | 1143.9 (888.3-1427.4) | 53 (41.2-66.2) | 3033.2 (2381.7-3717.6) | 101.9 (79.1-125) | -0.01 (-0.19-0.17) |
| Serbia | 49496.8 (37476.4-62675.1) | 514 (389.2-650.9) | 58777.7 (44548.2-75051.3) | 398.7 (302.4-508.7) | -0.58 (-0.75--0.42) |
| Montenegro | 2458.3 (1907.3-3177.1) | 392.6 (304.6-507.4) | 3627.4 (2849.8-4573.4) | 397.3 (311.6-498.4) | 0 (0-0) |
| Belgium | 76852.7 (71790.5-81383.6) | 770.2 (719.4-815.6) | 58650 (51955-64479.1) | 292 (263.3-318.8) | -2.16 (-2.31--2.01) |
| Solomon Islands | 357.2 (203.9-518) | 105.4 (60.1-152.8) | 1679.9 (1196.7-2335.1) | 352.3 (250.1-480.7) | 1.84 (1.73-1.96) |
| Central African Republic | 3894.6 (2728.6-5390.2) | 142.6 (99.9-197.4) | 9321.6 (6266.6-13197.4) | 304.5 (213.6-416.9) | 0.33 (0.27-0.39) |
| Andorra | 210.2 (147.3-305.5) | 386.7 (271-562) | 387.2 (268-536.7) | 262.2 (179.8-365.3) | -0.57 (-0.74--0.4) |
| Cyprus | 2919 (2430.1-3509.5) | 375.2 (312.3-451.1) | 5671.7 (4658.3-6892.6) | 297.4 (244-358.4) | -0.56 (-0.71--0.42) |
| Cuba | 30498 (28440.3-32508.3) | 281.2 (262.2-299.7) | 47806.6 (40482-56815.3) | 262.4 (222.2-310.6) | 0 (0-0) |
| Argentina | 154717.5 (145535-162693.9) | 467.3 (439.5-491.4) | 193925.4 (177486.8-209369.3) | 361.8 (331.2-391.2) | 0 (0-0) |
| Yemen | 4758.3 (2849.5-6813.7) | 34.9 (20.9-50) | 25236.6 (16954.8-36674.7) | 128.9 (89.7-184.2) | 1.83 (1.7-1.96) |
| Russian Federation | 551746.9 (536224.9-567334.1) | 365.4 (355.2-375.8) | 691770.6 (615179.7-765873.4) | 309.6 (274.2-342.8) | -0.45 (-0.72--0.18) |
| Tonga | 331.9 (263.6-422.3) | 335.7 (266.7-427.2) | 497.3 (358.9-662.6) | 588 (427.5-782) | 0.14 (0.07-0.21) |
| Thailand | 87496 (70739-104643.7) | 154.1 (124.6-184.3) | 287075.6 (214697.3-366179.2) | 283.7 (213.7-363.4) | 1.23 (1-1.46) |
| Dominica | 251.6 (214.9-289.1) | 347.4 (296.8-399.1) | 369.5 (279.2-466.3) | 451.2 (341.3-566.3) | 0.11 (0.03-0.19) |
| Slovakia | 20669.4 (18034.7-24022.9) | 391.2 (341.4-454.7) | 26473.4 (20440.4-33172.7) | 297.9 (232.7-369.8) | -0.66 (-0.75--0.57) |
| Turkmenistan | 5525 (4817.8-6277.1) | 149.4 (130.2-169.7) | 11018.8 (8171.7-14845.2) | 227.2 (169.1-304.6) | 0.21 (-0.23-0.65) |
| Botswana | 1819.2 (1223.9-2640.6) | 137.9 (92.8-200.2) | 6051.4 (4034.1-9038.3) | 334.2 (236.4-479) | 0 (0-0) |
| Denmark | 44745.3 (42272.4-47082.1) | 869.9 (821.8-915.3) | 28197.4 (25309.2-31150) | 269.3 (244.1-297) | -2.76 (-2.88--2.65) |
| Slovenia | 8842.4 (8111.9-9660.7) | 448 (411-489.5) | 8961.1 (7390.1-10769) | 221.8 (182.5-267.4) | -1.84 (-2.07--1.61) |
| Coted'Ivoire | 12986.2 (9843-16915.1) | 106.5 (80.7-138.7) | 48141.9 (34213.4-66487.5) | 320.9 (233.6-431.1) | 0 (0-0) |
| Kenya | 19697.9 (13949.6-26920.7) | 85.1 (60.3-116.3) | 97013.5 (66223.2-134641.2) | 328.6 (230.6-451.2) | 0 (0-0) |
| Tajikistan | 7592.8 (6079.8-9183.2) | 141.4 (113.2-171) | 14005.3 (8450.9-21176.9) | 180 (113.2-265.7) | -1.17 (-1.28--1.05) |
| Brunei Darussalam | 426.4 (313.4-560) | 164.5 (120.9-216) | 1433.1 (1106.4-1759.6) | 305 (236.8-373.6) | 0.92 (0.68-1.16) |
| Finland | 25316.9 (23815.2-26962.9) | 505.3 (475.4-538.2) | 23913.9 (21100.2-26695.4) | 233.1 (209.2-257.6) | -1.58 (-1.65--1.5) |
| Japan | 236308.1 (225811.5-245841.9) | 187.8 (179.5-195.4) | 427448.5 (378949.9-464798.8) | 180.4 (166.4-192.1) | 0.73 (0.56-0.9) |
| Cambodia | 13708.1 (8594.7-20848.1) | 133.4 (83.7-202.9) | 51430.4 (36711.2-69586) | 354.4 (253.9-474.9) | 1.16 (1.1-1.22) |
| Uzbekistan | 30767.8 (27783.9-33739.6) | 146.8 (132.6-161) | 67134.9 (55522.2-80476.3) | 205 (170.5-246) | -0.48 (-0.68--0.28) |
| Kuwait | 1310.5 (1169.3-1457) | 76.3 (68-84.8) | 6754.7 (5666-7962.2) | 141.2 (117.1-163.5) | -0.27 (-0.73-0.18) |
| Madagascar | 16666.3 (12965.2-21319.9) | 140 (108.9-179.1) | 47835.6 (33731.5-65381.6) | 295.9 (211.2-393.6) | 0.22 (0.02-0.42) |
| Austria | 47760.1 (44602.1-50842.7) | 614.8 (574.2-654.5) | 38264 (34050.8-42276.7) | 239.2 (216.3-262.8) | -1.95 (-2.02--1.87) |
| Vanuatu | 169.9 (113.8-244.1) | 111.6 (74.8-160.3) | 726.7 (518.9-961.6) | 329.8 (240.1-429.7) | 1.29 (1.17-1.41) |
| Lesotho | 2045.2 (1406.2-2889.3) | 133.4 (91.8-188.5) | 5415.1 (3376.5-7953.6) | 443.9 (280.7-646.6) | 3.04 (2.59-3.48) |
| Guyana | 1495.5 (1289.7-1729.3) | 191.8 (165.4-221.8) | 3039.5 (2251.2-4008.8) | 422.2 (314.8-554.3) | 1.22 (0.96-1.49) |
| Indonesia | 294162.8 (204108.1-415463.2) | 159 (110.3-224.6) | 909231.3 (610335.4-1311906.7) | 309.8 (208.6-445.4) | 0.82 (0.71-0.93) |
| Chile | 29458.1 (27569.5-31393.4) | 221.7 (207.5-236.3) | 47591.8 (43040.7-52111.1) | 194.6 (176.4-213.1) | -0.91 (-1--0.81) |
| Lao People's Democratic Republic | 5859.5 (3344.3-9562.5) | 140.5 (80.2-229.3) | 17425.4 (12257.7-23996.2) | 297.1 (209.7-409.1) | 0.8 (0.74-0.86) |
| Sierra Leone | 3528.4 (2421.2-4767.4) | 85 (58.3-114.8) | 11042.5 (7593.1-15009.8) | 226 (160.3-303.1) | 0 (0-0) |
| Ukraine | 300954.4 (279974.5-323822.2) | 570.9 (531.1-614.3) | 213682.1 (136483.5-315056.6) | 302.5 (190.2-453) | -1.79 (-2.03--1.55) |
| Bolivia (Plurinational State of) | 9635.3 (6262.1-13957.5) | 151 (98.1-218.7) | 28457.6 (18811.1-40802.7) | 280.5 (185.7-402.7) | 0 (0-0) |
| Uruguay | 20867.1 (19674.9-22023.8) | 664.7 (626.7-701.5) | 22140.9 (20096.3-23979.2) | 456.3 (417.5-492.3) | -0.89 (-0.97--0.81) |
| Haiti | 13905 (8250.6-21860.8) | 217.9 (129.3-342.6) | 38514.6 (23627.4-60450.5) | 411 (254.2-638) | 0.58 (0.5-0.66) |
| Ecuador | 9047.6 (8470.7-9626) | 90.7 (84.9-96.5) | 34030.2 (25920.8-43430.3) | 200.3 (152.9-255.2) | 1.2 (0.98-1.41) |
| Jamaica | 5337.2 (4954-5773.3) | 225.6 (209.4-244.1) | 13812.6 (10380.7-17683.7) | 448.2 (337-574.3) | 1.12 (0.81-1.43) |
| Venezuela (Bolivarian Republic of) | 27894.1 (26260-29574.1) | 148.3 (139.6-157.2) | 104482.4 (79091.7-134683.8) | 335.8 (254.6-432.7) | 0 (0-0) |
| American Samoa | 111.2 (90.9-136.5) | 229.2 (187.3-281.5) | 314.7 (245.4-390.5) | 609.9 (478.4-757.1) | 1.72 (1.61-1.84) |
| Iceland | 1122.2 (1030.4-1208.3) | 442 (405.9-475.9) | 1305.5 (1138-1467.6) | 253.1 (223.1-283.8) | -1.73 (-1.84--1.63) |
| Puerto Rico | 11339.9 (10531.6-12078.6) | 313.9 (291.5-334.4) | 14792 (12303.2-17405) | 264.4 (218.4-312.6) | -0.71 (-0.84--0.59) |
| Ireland | 19658.9 (18560.8-20974.3) | 545.8 (515.3-582.3) | 19244.6 (17125.4-21409.3) | 266.3 (239.1-296.3) | -2.05 (-2.15--1.95) |
| Latvia | 12588.9 (11135.2-14284.5) | 473.6 (418.9-537.4) | 9989.3 (8153-11847.3) | 295.1 (239.2-351) | -0.84 (-1.06--0.63) |
| Albania | 3419.4 (2679.3-4298.4) | 103.5 (81.1-130.1) | 6215.5 (4457.6-8277.6) | 162.9 (116.7-216.6) | 0.79 (0.59-1) |
| Lithuania | 14475.3 (13409-15593.3) | 394 (364.9-424.4) | 13805.7 (11519.1-16141.8) | 286 (239.7-333.1) | -0.55 (-0.77--0.33) |
| Canada | 135762.9 (126248.4-144795.9) | 498.2 (463.2-531.3) | 149682.4 (133578.9-165918.6) | 241.1 (217.6-266.9) | -1.98 (-2.05--1.91) |
| Netherlands | 99104.9 (92709.7-105696.8) | 664.2 (621.3-708.3) | 89475.8 (80925.7-98665.6) | 299.3 (274.6-327.2) | -2.05 (-2.18--1.92) |
| Poland | 148116.4 (143078.5-153143.4) | 388 (374.8-401.2) | 199631 (175028.4-222995.6) | 307.9 (270.1-344.2) | -0.57 (-0.68--0.46) |
| Australia | 75310.7 (71187.9-79819.9) | 446.7 (422.3-473.5) | 92252.8 (81488.3-102679.6) | 235.6 (210.6-262.3) | -1.84 (-1.9--1.78) |
| Italy | 363303.7 (345212.1-377607.4) | 639.6 (607.8-664.8) | 329328.6 (291495.5-357262.6) | 275.5 (251.1-296.3) | -1.73 (-1.79--1.66) |
| Brazil | 280324.5 (270207-290006.1) | 188.8 (181.9-195.3) | 741656.2 (698069.4-782334) | 288.1 (271-303.9) | -0.03 (-0.1-0.04) |
| Lebanon | 7058.8 (5026.1-9520.5) | 235.9 (168-318.2) | 19770.2 (15847.1-24146.7) | 334.8 (268.3-409.5) | 0.64 (0.41-0.86) |
| Fiji | 2513.3 (1974.6-3194.1) | 331.4 (260.4-421.2) | 5107.4 (3727.1-6810.9) | 595.2 (440.4-786.1) | 0.63 (0.49-0.77) |
| Israel | 22245.8 (20831.2-23758) | 448.4 (419.9-478.9) | 30656 (26927.7-34010.3) | 270 (240.6-297.7) | 0 (0-0) |
| New Zealand | 19813.4 (18513.9-21290.8) | 579.8 (541.8-623) | 21158.1 (19083.1-23163.7) | 290.5 (265-317.3) | -1.88 (-1.95--1.81) |
| Congo | 4756.4 (2751.6-7718.7) | 198 (114.6-321.4) | 17199.6 (10155.5-27460.6) | 458.6 (280.4-717.5) | 0.51 (0.39-0.64) |
| Saint Kitts and Nevis | 225.8 (206.5-246.6) | 544.6 (498-594.6) | 311.5 (253.2-377.3) | 420.5 (343.3-506) | -1.14 (-1.37--0.9) |
| Bermuda | 354.1 (316.2-395.5) | 596.3 (532.5-666.1) | 357.2 (296.4-448.9) | 304 (250.4-380.3) | -2.35 (-2.57--2.13) |
| Togo | 3553.9 (2818.4-4512.5) | 97.5 (77.3-123.7) | 14675.6 (10194-20135.8) | 289.2 (207-396.5) | 0.95 (0.86-1.03) |
| Dominican Republic | 8254.6 (6942.4-9696) | 115.4 (97.1-135.6) | 23353.1 (17764.1-30695.3) | 220.3 (167.8-289) | 0.83 (0.66-0.99) |
| Peru | 28190.1 (23024.8-33487.3) | 130.3 (106.4-154.8) | 67061 (48574.2-89411.1) | 189 (137.2-250.8) | -0.64 (-0.85--0.43) |
| Kiribati | 180.9 (140.6-230.3) | 243.2 (189-309.6) | 481.6 (355.6-656.5) | 540 (405.4-727.2) | 1.13 (1.06-1.2) |
| United States of America | 1423203.8 (1355187.7-1488967.3) | 560.1 (533.3-586) | 1423645.9 (1324855.6-1521530) | 277.4 (260.1-294.8) | -1.93 (-2--1.86) |
| Norway | 21388.2 (20257.9-22416.5) | 503.7 (477.1-527.9) | 17177.6 (15544.8-18587.3) | 194.6 (178.4-210) | -2.34 (-2.52--2.16) |
| Costa Rica | 3922.5 (3627.3-4235.9) | 129 (119.3-139.3) | 15215.4 (13342.3-17335.8) | 276.9 (243-315.5) | 1.1 (0.94-1.25) |
| Trinidad and Tobago | 3751.9 (3466.8-4072.1) | 311.4 (287.7-338) | 7703.8 (5851.2-9889.2) | 414.5 (315-534.5) | -0.08 (-0.25-0.1) |
| Azerbaijan | 17542.8 (14562.5-20125.3) | 239.4 (198.8-274.7) | 29616.1 (21917.5-38131.2) | 245.6 (183.1-313.2) | -0.8 (-0.91--0.69) |
| Marshall Islands | 69.4 (48.6-98.3) | 152.9 (107-216.4) | 224.6 (132.6-356.2) | 480.3 (289.8-744.8) | 1.13 (1.01-1.25) |
| El Salvador | 4543.7 (3918.3-5214.4) | 85.6 (73.9-98.3) | 13547.2 (10736.2-17267.3) | 223.1 (176.8-284.4) | 1.61 (1.5-1.72) |
| Portugal | 52509.8 (49467.3-56034.1) | 518 (488-552.8) | 49836.6 (44452.7-54124.8) | 255.8 (231.9-278.3) | -1.7 (-1.8--1.61) |
| Malawi | 10329.1 (7940.9-12854) | 105.3 (81-131.1) | 31516.2 (22450.7-41999.6) | 325.1 (235.2-432.3) | 1.29 (1.19-1.38) |
| Namibia | 2278.7 (1871.5-2812.6) | 162.3 (133.3-200.3) | 8937.7 (5679.5-12877.1) | 535.9 (346.9-752.2) | 2.16 (2.02-2.3) |
| China | 1495722 (1208227.3-1828360.2) | 127.1 (102.7-155.4) | 3029404.7 (2360641.2-3844035.9) | 146.3 (113.8-185.5) | -0.36 (-0.45--0.26) |
| Gambia | 396.5 (288.9-537.4) | 40.4 (29.4-54.8) | 1896 (1356.3-2587.9) | 155.2 (112.4-208.6) | 1.5 (1.28-1.72) |
| Luxembourg | 2657.1 (2494.7-2827.9) | 697.1 (654.5-741.9) | 2484.2 (2225.2-2761.4) | 247.2 (221.2-274) | -2.07 (-2.23--1.91) |
| Libya | 2893.4 (2262.2-3791.8) | 68.6 (53.7-90) | 14626.7 (10657.5-20609.4) | 209.3 (155.4-288.6) | 1.69 (1.47-1.91) |
| South Africa | 72651.3 (59670.3-86106.2) | 196.3 (161.2-232.6) | 202893.2 (181348-227019.6) | 393.5 (354.8-436.6) | 1.4 (1.16-1.63) |
| Ghana | 22861.8 (17513.4-29267.8) | 152.7 (117-195.5) | 71724.7 (52776.5-97129.7) | 330.7 (246.7-440) | 0.4 (0.33-0.47) |
| Oman | 491.5 (349.1-678.5) | 24.8 (17.6-34.2) | 1753 (1318.6-2319.7) | 61.6 (47.3-78.2) | 0.56 (0.17-0.94) |
| Georgia | 30994 (27722.9-34433.7) | 561.2 (502-623.5) | 25290.2 (21672.7-29331.6) | 467.7 (400.2-543.7) | 0.03 (-0.22-0.27) |
| Qatar | 508.9 (397.1-630.8) | 114.4 (89.3-141.8) | 3397.6 (2400.3-4710.6) | 216.9 (160.3-290.7) | -0.23 (-0.43--0.02) |
| Timor-Leste | 598.7 (372.7-921.9) | 76.6 (47.7-118) | 1999.6 (1364.4-2727.5) | 214.2 (145.8-292.1) | 1.29 (1.06-1.51) |
| Cook Islands | 77 (58.5-99.9) | 407.1 (309.3-528) | 135.5 (102-175.6) | 579.8 (432.7-753.3) | 0.31 (0.13-0.5) |
| Mauritius | 1625 (1513.7-1757.5) | 148.3 (138.1-160.4) | 6811.6 (6143.2-7307.2) | 381.7 (343.1-410.7) | 1.6 (1.28-1.92) |
| Kazakhstan | 53793 (47914-59975.9) | 328.1 (292.3-365.9) | 44284.5 (37044.7-51755.9) | 225.4 (188.8-263) | -1.32 (-1.65--0.98) |
| Bhutan | 388.7 (262.3-516.8) | 61.7 (41.6-82) | 917.5 (627.9-1273.6) | 133 (91.8-182.7) | 0.23 (0.11-0.35) |
| Burundi | 8049.6 (5641.7-11556.4) | 145 (101.6-208.1) | 15748.9 (11246.7-21862.5) | 238.2 (173.8-327.5) | -1.06 (-1.25--0.88) |
| India | 843324.6 (723417-983728.4) | 98.9 (84.8-115.3) | 2758796.7 (2342795.8-3257229.7) | 206.2 (175.1-243.9) | 1.26 (1.11-1.42) |
| Saudi Arabia | 8154.4 (5848.5-11239.6) | 51.4 (36.9-70.9) | 53438.6 (37359.1-74891.2) | 139.8 (103.4-189.2) | 1.38 (1.09-1.66) |
| Democratic Republic of the Congo | 44138.1 (30731.8-60414.9) | 115.7 (80.6-158.4) | 139923.9 (103412.3-192789.5) | 292.1 (215.3-402.6) | 0.86 (0.64-1.08) |
| San Marino | 96.4 (76.3-120.8) | 405.8 (321.3-508.7) | 106.6 (64.4-156.5) | 176.5 (104.3-262.6) | -0.95 (-1.22--0.69) |
| Morocco | 16346.8 (12358.1-21774.3) | 64.5 (48.7-85.9) | 63083.6 (41712.3-93314.2) | 163.9 (109.6-239.9) | 1.86 (1.76-1.95) |
| Malta | 2285.6 (2121.8-2469.5) | 616.8 (572.5-666.4) | 2391.8 (2119.4-2699.7) | 304.6 (271.3-340.6) | -2.07 (-2.18--1.97) |
| Greenland | 197.6 (155.6-250.1) | 355.7 (280-450.2) | 187.3 (135.9-249) | 254.2 (187.6-333.5) | -2.24 (-2.37--2.1) |
| Nicaragua | 2079.1 (1774.8-2404.1) | 53.5 (45.7-61.8) | 8541.3 (6615.7-10748.9) | 153.2 (119-192) | 1.39 (1.2-1.57) |
| Panama | 2981.3 (2756.5-3216.2) | 124.8 (115.4-134.6) | 10698.4 (8396.4-12982.8) | 242 (190.1-293.7) | 1.14 (1.03-1.25) |
| Algeria | 15897.8 (11996.1-20724.9) | 62.9 (47.4-82) | 53601.2 (40143.5-70802.3) | 128.9 (97.5-167.9) | 0.47 (0.38-0.56) |
| Comoros | 686.4 (485.5-934.8) | 148.4 (105-202.1) | 2200.3 (1635.6-2972.9) | 379.9 (283.1-507.2) | 0.65 (0.5-0.8) |
| Djibouti | 547.4 (388.5-741.7) | 132.1 (93.8-179.1) | 2951.7 (1917.5-4419.9) | 335.5 (226.5-486.4) | 0.48 (0.42-0.54) |
| Nepal | 14707.5 (10881.6-19407.4) | 75.5 (55.9-99.7) | 40334.2 (28781-55812.8) | 154 (110.4-212.4) | 0.79 (0.52-1.06) |
| Seychelles | 151.5 (129.6-175.1) | 207.8 (177.9-240.3) | 429.1 (362.9-500.9) | 338.3 (287.1-396.3) | 0.74 (0.44-1.05) |
| Somalia | 8106.4 (5440.6-11377.7) | 102.1 (68.5-143.3) | 21235.1 (13965.3-30171.8) | 244.6 (166.4-336.4) | 0.16 (0.11-0.2) |
| Equatorial Guinea | 593.8 (390.7-870.1) | 140.4 (92.4-205.8) | 2975 (1702.5-4809) | 415.7 (247-652.1) | 1.69 (1.59-1.8) |
| Colombia | 48545.2 (45347.6-51945.8) | 149.4 (139.6-159.9) | 134544.7 (111840.7-162240.4) | 244.7 (203.6-295.3) | 0.15 (-0.08-0.38) |
| Tokelau | 5.6 (3.6-8) | 349.2 (223.7-504.3) | 7.3 (5.4-9.7) | 513.9 (383.9-681.3) | 0.32 (0.26-0.38) |
| Mali | 11045.2 (9003.1-13390.7) | 127.5 (103.9-154.6) | 28998.6 (20330.2-40529.4) | 258.4 (183.4-354.2) | 0.29 (0.21-0.38) |
| Grenada | 289.9 (260.4-321.3) | 333.2 (299.3-369.3) | 548.9 (472.8-629.4) | 471.9 (407.9-537.5) | 0.45 (0.16-0.73) |
| Czechia | 51238.2 (46145.6-56986.5) | 497.7 (448.3-553.6) | 45754.6 (38353.5-53808.6) | 239.8 (200.5-282.1) | -1.89 (-2.06--1.72) |
| Mauritania | 2467.9 (1750.5-3343.4) | 120.1 (85.2-162.7) | 6878.4 (5164.5-9246.3) | 272.3 (206-364.5) | 0.45 (0.33-0.57) |
| Tuvalu | 29.6 (19.2-43.5) | 311.2 (202.4-457) | 48.8 (35-67.4) | 444.6 (320.2-611.1) | 0.28 (0.2-0.35) |
| Benin | 4136.6 (3323.6-5018.1) | 85.3 (68.5-103.5) | 13802.9 (9800.9-18705.8) | 209.7 (153.4-278.8) | 0.38 (0.29-0.47) |
| Hungary | 60417.9 (53753.7-67889.2) | 581.2 (517.1-653.1) | 53979.8 (46089.3-62982.2) | 315.7 (267.7-370.5) | 0 (0-0) |
| United Republic of Tanzania | 35993.4 (28537.4-44162.9) | 139.3 (110.4-170.9) | 113525.1 (82850-151629.3) | 345.1 (255-459.7) | 0.65 (0.58-0.72) |
| Democratic People's Republic of Korea | 35186.9 (22980.6-51903.2) | 170.9 (111.6-252.1) | 69130.5 (46575.2-94059.5) | 201.1 (137.3-273.3) | 0.45 (0.38-0.52) |
| United Arab Emirates | 1698 (1204.7-2323) | 90.8 (64.4-124.2) | 13218.9 (9609.5-17680.4) | 220.4 (161.4-288.6) | 0 (0-0) |
| Niger | 4715.3 (3454.9-6262.3) | 58.7 (43-78) | 15275.6 (10383.1-21376.5) | 147.9 (101.8-205.1) | 0.35 (0.26-0.44) |
| Afghanistan | 11903 (5896.1-20811.4) | 119.7 (59.3-209.3) | 37533.2 (18285.3-69699.4) | 244.7 (129.6-424.6) | 1.43 (1.35-1.51) |
| Viet Nam | 67434.2 (51474.4-87987.4) | 98.8 (75.4-129) | 212025.6 (159236.5-286747.9) | 191.1 (144-255.2) | 0.69 (0.65-0.73) |
| Palestine | 2994.7 (2115.9-4192.6) | 146.3 (103.4-204.8) | 10603.7 (8604.4-13014) | 331.6 (267.8-405.5) | 0.46 (0.35-0.58) |
| Uganda | 21500.3 (15428-29356.3) | 124.3 (89.2-169.8) | 86321.5 (61475.2-116772.6) | 448.6 (330.1-600.8) | 0.87 (0.57-1.16) |
| Monaco | 295.7 (218.7-391.8) | 972.2 (719-1288.2) | 412.2 (309.2-545.1) | 557.2 (408.8-749.5) | 0.2 (0.1-0.31) |
| Burkina Faso | 15818.1 (12062.3-20216.1) | 166 (126.6-212.2) | 40441.6 (27884.8-55503.9) | 342.6 (246.6-457.4) | 0.29 (0.17-0.4) |
| United Kingdom | 450529.5 (434340.4-464443.1) | 786.3 (758-810.6) | 319901.4 (295864.3-338483.6) | 286.3 (269.2-301.5) | -2.38 (-2.44--2.31) |
| Nigeria | 130571.9 (96350.9-171909.3) | 145 (107-190.9) | 522743.4 (346862.6-762662) | 453 (310.1-647.3) | 1.98 (1.76-2.2) |
| Barbados | 1325.1 (1214.6-1431.1) | 522.9 (479.3-564.7) | 2504.3 (2002.5-3118.8) | 538.3 (427.7-675.7) | 0.52 (0.33-0.71) |
| Cameroon | 12730.6 (10120-15741.2) | 122 (97-150.8) | 47109.9 (33289-65922.8) | 281.7 (201.4-387.9) | 0.53 (0.46-0.6) |
| Iraq | 19714.7 (14928.7-25893.2) | 107 (81.1-140.6) | 86961.2 (61387.3-117459.8) | 275.7 (195.3-367.2) | 0.89 (0.82-0.96) |
| Sudan | 10396 (6298-16421.2) | 51.9 (31.5-82) | 37457.5 (21813-60428.6) | 134.4 (82.3-213.9) | 1.35 (1.18-1.53) |
| Paraguay | 5025.4 (4041.3-6066) | 124.3 (100-150) | 17597.6 (13135.6-23396) | 278.1 (208.5-370.6) | 1.08 (0.89-1.27) |
| Guinea | 7184.6 (5391.6-9086.7) | 119.9 (89.9-151.6) | 18375.1 (12577.1-26183.7) | 263.5 (184.5-368.8) | 0.9 (0.87-0.93) |
| Angola | 11042.3 (7822-15621.3) | 107.5 (76.1-152) | 51271.5 (34998.6-69771.1) | 314.5 (216.3-423.5) | 1.4 (1.31-1.49) |
| Belize | 147.6 (133.8-161.1) | 79 (71.6-86.2) | 754.9 (661.9-858.3) | 212.2 (186.3-241.1) | 1.17 (0.89-1.45) |
| Jordan | 4155.2 (3139.3-5446.5) | 111.2 (84-145.8) | 22572.5 (15933.1-30510.7) | 233.8 (167-315.5) | 0.07 (-0.3-0.44) |
| Nauru | 28.9 (17-46) | 283.7 (166.6-450.6) | 47.9 (28.1-75.3) | 640.8 (381.9-994.5) | 1.17 (1.08-1.26) |
| Niue | 9.2 (6.9-11.9) | 397.6 (301.1-516.4) | 10.8 (8-14.3) | 534.5 (402.2-705.8) | 0.38 (0.29-0.47) |
| Gabon | 2082.1 (1477.3-2816.3) | 211.7 (150.2-286.3) | 5295.1 (3545.1-7468.2) | 418 (285.4-576.5) | 0.45 (0.33-0.57) |
| Guinea-Bissau | 1288.5 (885.7-1883.5) | 127.9 (87.9-187) | 3566.3 (2474.1-4930.9) | 338.5 (233.9-463.2) | 0.93 (0.91-0.95) |
| South Sudan | 5935.3 (4131.9-8402.9) | 101 (70.3-143) | 13234.7 (9149-19269.3) | 253 (177.7-361.4) | 0.63 (0.39-0.88) |
| Mozambique | 16727.5 (13533.6-20806.8) | 125.2 (101.3-155.7) | 50787.2 (34807.1-69750) | 347.4 (244.2-464.1) | 1.64 (1.5-1.77) |
| Saint Lucia | 428.3 (395.8-463.7) | 313.6 (289.8-339.6) | 861.7 (713.8-1049.8) | 362.3 (300.3-441.6) | -1.03 (-1.31--0.76) |
| Eswatini | 961.2 (722.5-1232.2) | 119.2 (89.6-152.8) | 3049.6 (1706.4-4837.9) | 442.2 (254.8-689.6) | 1.81 (1.37-2.26) |
| Liberia | 2165.3 (1651.9-2737.8) | 88 (67.1-111.3) | 7177.2 (4851-10687.9) | 235.4 (163.5-335.4) | 1.16 (1.02-1.29) |
| Taiwan (Province of China) | 29487.9 (27660.8-31221.7) | 144.6 (135.7-153.1) | 89175.6 (81447.7-96635.7) | 232.8 (212.9-251.6) | 1.23 (1.02-1.44) |
| Bosnia and Herzegovina | 10581.4 (9054.6-12011.1) | 235.2 (201.3-267) | 14668.4 (11358.5-18274.1) | 257.5 (197.2-322.9) | 0.72 (0.56-0.88) |
| Rwanda | 14279.7 (9857-20169.9) | 198.6 (137.1-280.6) | 30214 (20706.9-41758.8) | 381.4 (267.1-522.6) | -0.69 (-0.92--0.45) |
| Republic of Korea | 40557 (35722.4-48034.8) | 91.7 (80.7-108.6) | 98164.7 (79587.2-116771.3) | 118.5 (96.4-140.4) | 0.51 (0.43-0.6) |
| Bangladesh | 69479.8 (51785-94668.4) | 63.7 (47.5-86.8) | 235506.5 (175731.5-303371.2) | 148.1 (110.9-190.5) | 0.84 (0.69-1) |
| Bahrain | 825.2 (694.6-969.5) | 162.9 (137.2-191.4) | 3651.4 (2843.9-4731.2) | 287.4 (225.4-369.1) | -0.71 (-0.91--0.51) |
| Singapore | 7169.4 (6710.8-7658) | 235.3 (220.2-251.3) | 15128.9 (13912.8-16531.6) | 174.4 (160.1-190.4) | -1.04 (-1.21--0.86) |
| Armenia | 15462.7 (14427.5-16443.1) | 452 (421.8-480.7) | 13337.6 (11714.6-15279.2) | 315.3 (277.3-360.9) | -1.63 (-1.96--1.3) |
| Belarus | 39743.5 (35967.3-43886.3) | 380.5 (344.4-420.2) | 38587 (30134.1-48625.6) | 259.9 (201.2-330.2) | -1.56 (-1.84--1.28) |
| Estonia | 7364.5 (6758.1-8005.5) | 469.5 (430.9-510.4) | 5606.3 (4583.3-6639.2) | 239.9 (197.2-283.6) | -1.73 (-1.91--1.54) |
| France | 331631.6 (312392.7-351639.1) | 574 (540.7-608.7) | 348170.6 (305783.9-384297.7) | 301.6 (269.4-332) | -1.3 (-1.43--1.17) |
| Bulgaria | 41689.3 (37147.3-47311) | 480.3 (428-545.1) | 45217.6 (37047.1-54039.4) | 376.4 (308.6-450.5) | 0.3 (0.18-0.42) |
| Croatia | 23098.1 (20302.9-25889.9) | 475.1 (417.6-532.6) | 21820 (18665.4-25160.2) | 279.1 (234.9-319) | -1 (-1.18--0.83) |
| Antigua and Barbuda | 212.5 (192.1-235.9) | 352.8 (318.9-391.8) | 515.2 (479.1-556.2) | 462.5 (431.4-498.4) | 0.54 (0.34-0.73) |
| Guam | 218.8 (187.4-256.5) | 159.9 (137-187.5) | 473.8 (397-566.5) | 244.8 (204.7-289.9) | 0.54 (0.34-0.74) |
| Zimbabwe | 11599.2 (8943.4-15234) | 112.1 (86.5-147.3) | 45238.1 (32071.1-62053) | 505 (363.6-681.5) | 3.29 (2.53-4.05) |
| United States Virgin Islands | 468.5 (391.7-557) | 441.8 (369.3-525.1) | 490 (340.1-692.2) | 350.6 (243.4-494.9) | -0.68 (-0.82--0.55) |
| Bahamas | 1112.4 (1017.6-1218.7) | 433.3 (396.4-474.7) | 2775.1 (2221-3514.1) | 626.9 (503.8-788.6) | 0.3 (0.17-0.42) |
| Germany | 541608.7 (510901.2-575312.9) | 677.5 (639.1-719.7) | 472913.5 (424476.4-515359.8) | 294.2 (269-319.4) | -1.57 (-1.66--1.49) |
| Malaysia | 38319.5 (32265.5-45198.1) | 216.9 (182.6-255.8) | 131316.6 (110627.4-155133.1) | 417.9 (352.9-491.6) | 0.73 (0.6-0.87) |
| Spain | 191414.1 (178739.2-204114.8) | 493.6 (460.9-526.3) | 175311.5 (155267.9-193174.7) | 213.3 (193.2-234.2) | -2.08 (-2.15--2) |
| Micronesia (Federated States of) | 229.9 (153.7-319.8) | 222.2 (148.5-309.1) | 461.3 (327.5-621.3) | 526.6 (378.9-702.3) | 0.78 (0.72-0.84) |
| Greece | 56452.9 (53018.5-59631.7) | 543.4 (510.3-574) | 63052.3 (56959.9-68732.9) | 318.2 (292.9-344.8) | -1.03 (-1.16--0.9) |
| Saint Vincent and the Grenadines | 338.9 (303.5-370.4) | 309.5 (277.1-338.2) | 634.4 (547-732.4) | 456.8 (393.8-527.8) | -0.2 (-0.42-0.01) |
| Suriname | 759.8 (639.5-891.5) | 196.5 (165.4-230.5) | 1891.1 (1444.1-2416.8) | 290.2 (221.7-370) | 0.43 (0.3-0.56) |
| Sweden | 43947.3 (40773.1-47211.5) | 511.7 (474.8-549.8) | 35510.5 (29843.8-40850.3) | 194.7 (164.2-224.1) | -1.61 (-1.86--1.35) |
| Syrian Arab Republic | 9754 (7201.5-12456.9) | 76.7 (56.6-98) | 27922.9 (20234.5-37752.8) | 185.7 (136.8-249.2) | 0.59 (0.43-0.74) |
| Guatemala | 4097.8 (3825.6-4358.3) | 48.9 (45.6-52) | 18647.3 (15528.6-21800.2) | 151.8 (126.5-177.4) | 1.56 (1.3-1.82) |
| Tunisia | 8375.7 (6854.1-10129.1) | 100.3 (82.1-121.3) | 26088.5 (18416.8-35813.6) | 186.9 (132.4-255.5) | 0.6 (0.51-0.69) |
| Honduras | 3244.5 (2424.7-4108.6) | 68.9 (51.5-87.2) | 15618.8 (10948.3-21647) | 214.9 (153-296.6) | 1.58 (1.44-1.72) |
| Turkey | 39425.6 (31512.9-48523.1) | 68.6 (54.8-84.4) | 207685 (163346.4-257735.3) | 214.1 (168.3-265.3) | 3.81 (3.09-4.54) |
| Mexico | 98517.4 (95490-101193.5) | 115.4 (111.8-118.5) | 314959.8 (261636.4-372870.4) | 231.7 (193.1-273.7) | 0.36 (0.26-0.45) |
| Switzerland | 37256.9 (34678.8-39688.5) | 542.6 (505-578) | 33560.6 (29193.7-37020.4) | 207.7 (184.8-227.8) | -2.1 (-2.3--1.89) |
| Cabo Verde | 538.1 (431.3-662.8) | 152.1 (121.9-187.3) | 1092 (838.2-1393) | 220.8 (170.5-281.3) | -0.3 (-0.58--0.01) |
| Zambia | 10569.8 (7476.8-14667.4) | 133.2 (94.2-184.8) | 53708.5 (29184.6-86960.8) | 514 (301.9-792.1) | 2.11 (1.79-2.44) |
| Chad | 4672.9 (3333.6-6171.5) | 77.5 (55.3-102.4) | 14116.6 (9812.3-19076.1) | 184.8 (132-245.6) | 0.71 (0.68-0.75) |
| Northern Mariana Islands | 99.9 (69.4-135.6) | 221.4 (153.8-300.6) | 234.5 (191.5-275.5) | 404.3 (330.5-470.2) | 0.52 (0.39-0.66) |
| Palau | 69.3 (52.2-90.5) | 456.2 (343.8-595.5) | 129.9 (98.3-169.8) | 564.7 (433.8-730.3) | 0 (0-0) |
| Sao Tome and Principe | 116.6 (93.8-144.2) | 96.2 (77.3-118.9) | 377.2 (274.3-500) | 263.5 (197.4-345) | 1.24 (1.11-1.38) |
| Pakistan | 197483.7 (156600.9-243930.5) | 177.7 (140.9-219.5) | 745586.2 (529953.3-999586.6) | 457.5 (328.9-608.3) | 0.99 (0.77-1.21) |
| Egypt | 54808.4 (46771.6-65829.1) | 99.1 (84.5-119) | 212977 (166696.7-266182.8) | 268.9 (214.1-332.6) | 2.31 (1.97-2.66) |
| Senegal | 6866.1 (5316-8643.3) | 90 (69.7-113.2) | 24364.6 (18143.6-33232.6) | 261.3 (196.6-351) | 1.25 (1.12-1.38) |
| Iran (Islamic Republic of) | 36441.8 (31306.1-42093.9) | 63.8 (54.8-73.7) | 154475.4 (138491.8-172531.8) | 164.5 (147.6-183.6) | 1.76 (1.47-2.06) |
| Eritrea | 5164.4 (3738.4-7143.1) | 151.6 (109.8-209.7) | 15708 (10842.8-21822.5) | 417.2 (294-569.8) | 0.83 (0.73-0.92) |
| Kyrgyzstan | 10082.6 (8932.8-11354.9) | 225.9 (200.1-254.4) | 11538.3 (9359-14030.9) | 200.7 (164.1-242.4) | -1.69 (-1.85--1.52) |
| Ethiopia | 74295.2 (48027.8-111677.9) | 146.9 (95-220.8) | 167188.4 (134337.3-206546.9) | 292.5 (238.4-360.3) | -0.27 (-0.44--0.1) |
| Republic of Moldova | 17902.3 (15891.1-19900.8) | 402.5 (357.3-447.5) | 16991.7 (14388.2-20252.3) | 298.8 (252.7-356) | 0 (0-0) |
